# Glycomics and Glycoproteomics Reveal Distinct Oligomannose Carriers Across Bladder Cancer Stages

**DOI:** 10.3390/ijms26104891

**Published:** 2025-05-20

**Authors:** Marta Relvas-Santos, Dylan Ferreira, Andreia Brandão, Luis Pedro Afonso, Lúcio Lara Santos, André M. N. Silva, José Alexandre Ferreira

**Affiliations:** 1 Experimental Pathology and Therapeutics Group, Research Center of IPO Porto (CI-IPOP)/CI-IPOP@RISE (Health Research Network), Portuguese Oncology Institute of Porto (IPO Porto)/Porto Comprehensive Cancer Center Raquel Seruca (Porto.CCC Raquel Seruca), 4200-072 Porto, Portugal; marta.relvas.santos@ipoporto.min-saude.pt (M.R.-S.); luis.afonso@ipoporto.min-saude.pt (L.P.A.); lucio.santos@ipoporto.min-saude.pt (L.L.S.); 2 ICBAS—School of Medicine and Biomedical Sciences, University of Porto, 4050-513 Porto, Portugal; andre.silva@fc.up.pt; 3 LAQV-REQUIMTE, Department of Chemistry and Biochemistry, Faculty of Sciences, University of Porto, 4169-007 Porto, Portugal; 4 School of Medicine and Biomedical Sciences, Fernando Pessoa University, 4420-096 Gondomar, Portugal; 5 Cancer Genetics Group, Research Center of IPO Porto (CI-IPOP)/CI-IPOP@RISE (Health Research Network), Portuguese Oncology Institute of Porto (IPO Porto)/Porto Comprehensive Cancer Center Raquel Seruca (Porto.CCC Raquel Seruca), 4200-072 Porto, Portugal; andreia.aguiar.brandao@ipoporto.min-saude.pt; 6 Department of Pathology, Portuguese Oncology Institute of Porto (IPO Porto)/Porto Comprehensive Cancer Center Raquel Seruca (Porto.CCC Raquel Seruca), 4200-072 Porto, Portugal; 7 Department of Surgical Oncology, Portuguese Oncology Institute of Porto (IPO-Porto), 4200-072 Porto, Portugal; 8 GlycoMatters Biotech, 4500-162 Espinho, Portugal

**Keywords:** bladder cancer, *N*-glycomics, *N*-glycoproteins

## Abstract

Aberrant glycosylation is a hallmark of cancer, offering opportunities to enhance clinical decision-making and enable precise targeting of cancer cells. Nevertheless, alterations in the bladder urothelial carcinoma (BLCA) *N*-glycome remain poorly characterized. Here, we used in situ *N*-deglycosylation and mass spectrometry, revealing a marked enrichment of oligomannose-type *N*-glycans in non-invasive Ta tumors, which diminished with disease progression. A complementary analysis of The Cancer Genome Atlas (TCGA) transcriptomic data revealed downregulation of the key mannosidases in BLCA, suggesting a mechanistic basis for oligomannose accumulation, though this requires further validation. Then, targeted glycoproteomic profiling identified potential stage-specific carriers of oligomannoses. Exploratory functional annotation suggests stage-dependent differences among detected glycoproteins, ranging from metabolic regulation in Ta tumors to oxidative stress adaptation in muscle-invasive disease, highlighting glycosylation’s contribution to tumor progression. Furthermore, myeloperoxidase (MPO) was enriched in more aggressive stages. Spatial validation confirmed MPO overexpression in tumor-infiltrating immune cells and its correlation with oligomannose content. Importantly, high MPO expression combined with low mannosidase levels was linked to poor survival, suggesting biological relevance. This study suggests a dynamic, stage-specific *N*-glycome in BLCA and identifies oligomannose-bearing glycoproteins as exploratory leads for biomarker and therapeutic target discovery, providing a *N*-glycomic resource for further investigation towards glycan-based precision oncology.

## 1. Introduction

Bladder urothelial carcinoma (BLCA) is one of the most prevalent urological malignancies worldwide, with urothelial carcinoma accounting for most cases [1]. It is also among the costliest cancers to treat due to its high recurrence rates, the need for long-term surveillance, and the frequent requirement for invasive interventions, such as transurethral resection, intravesical therapies, and, in advanced cases, radical cystectomy with urinary diversion [2]. Non-muscle-invasive bladder cancer (NMIBC) often necessitates repeated treatments and lifelong monitoring, while a substantial subset of NMIBC cases progresses to muscle-invasive disease (MIBC), further complicating treatment [3]. MIBC and metastatic bladder cancer require multimodal approaches, including chemotherapy and immune checkpoint inhibitors, which are both costly and associated with significant side effects [4]. Given the high recurrence rates and unpredictable progression of the disease, there is an urgent need for more precise molecular strategies to improve early detection, predict disease progression, and enable targeted therapeutic interventions for both NMIBC and MIBC, ultimately reducing recurrence and improving patient outcomes.

The glycocalyx, a dense and intricately structured network of glycans and glycoproteins covering the cell surface, has emerged as a promising yet underexplored target for bladder cancer detection and therapy [5]. Aberrant protein glycosylation is a well-established hallmark of cancer, profoundly influencing tumorigenic processes such as invasion, metastasis, immune evasion, and therapy resistance [6]. Glycans play a pivotal role in apoptotic signaling, contributing to chemotherapy resistance, while also modulating immune checkpoint regulation and tumor cell recognition by cytotoxic lymphocytes [7]. These alterations shape the efficacy of immune checkpoint inhibitors and disrupt the organization of cell-surface receptors, impairing recognition by monoclonal antibody-based therapies [8,9]. Protein-associated glycans exhibit structural and functional diversity, falling into two major classes with distinct, yet synergistic, implications for tumor progression. *N*-glycans, covalently attached to the asparagine (Asn) residues of glycoproteins, undergo profound alterations in cancer, including increased oligomannosylation, aberrant core and outer-arm fucosylation, and hyperbranched complex-type structures [10,11]. These modifications influence cell adhesion dynamics, receptor clustering, and integrin signaling, thereby modulating epithelial-to-mesenchymal transition and metastatic potential [11,12]. *O*-glycans, which are linked to serine or threonine residues, represent the other main class and are frequently found in truncated or incomplete forms in most solid tumors. Notably, Tn and sialyl–Tn antigens, hallmark features of aggressive bladder tumors and circulating tumor cells, are rarely found in a healthy urothelium, highlighting their potential for precise cancer targeting [13,14]. These truncated *O*-glycans drive tumor invasion by modulating the functional role of key cell adhesion molecules, such as CD44 [15]. Furthermore, immature glycosylation patterns in BLCA cells have been shown to induce a tolerogenic phenotype in both innate and adaptive immune cells, facilitating immune evasion [16]. Given their pivotal role in tumor progression and immune evasion, cancer-associated glycan signatures are promising therapeutic targets. Accordingly, therapeutic antibodies and cancer glycovaccines targeting these immature glycans have been developed, offering a pathway to more precise and effective immunotherapies [17,18]. Additionally, advances in glycoproteomics enable the identification of cancer-specific glycan signatures and are driving precision oncology, thereby facilitating personalized strategies. For instance, we previously linked immature mucin-16 *O*-glycoproteoforms to resistance to cisplatin-based chemotherapy in MIBC, underscoring the relevance of glycoproteomic analysis for improving patient stratification [19].

Nevertheless, while glycosylation changes in solid tumors have been extensively studied, and significant progress has been made in elucidating *O*-glycome and glycoprotome in BLCA, the *N*-glycome remains largely uncharted, with its role in tumor progression and therapeutic targeting still poorly understood. Here, we employ an integrative glycomics and glycoproteomics approach to systematically profile the BLCA *N*-glycome across different disease stages, with a particular focus on potential glycoprotein carriers. By characterizing the tumor-specific glycosylation landscape, we aim to identify glycosidic candidates that could serve as the foundation for future biomarkers of disease progression and potential targets for precision therapies.

## 2. Results

Envisaging a rationale for glycan-based precision oncology in BLCA, we began by comprehensively characterizing the *N*-glycome of BLCA tumors across different disease stages as a foundation for deeper, targeted investigations into the *N*-glycoproteome, aiming to identify signatures associated with cancer aggressiveness.

### 2.1. BLCA Glycomics

**Figure 1:**
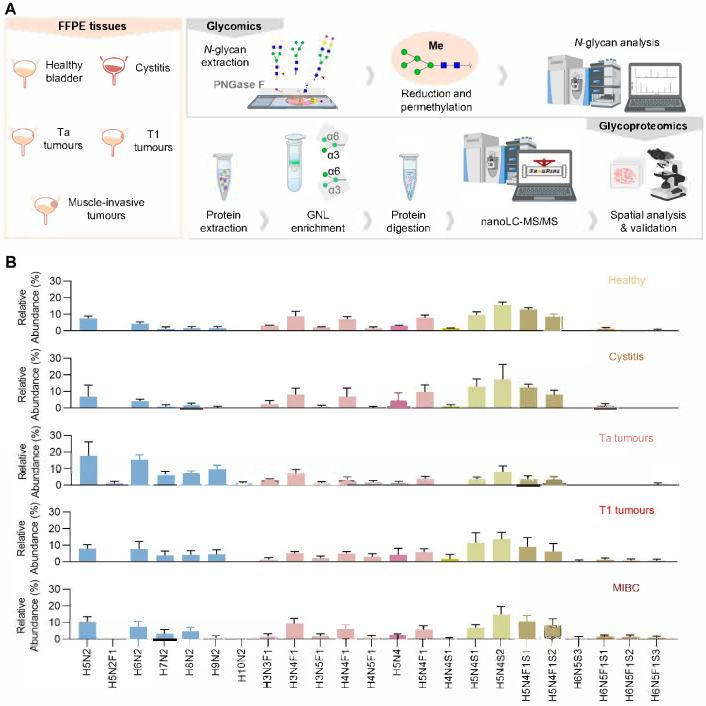

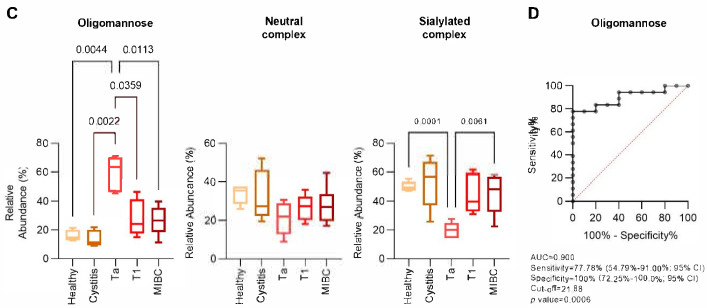
Glycomic profiling of bladder tissues across disease states. (**A**) Schematic overview of the analytical pipeline used to examine the pattern of *N*-glycans and *N*-glycoproteins across tumor stages. Permethylated and reduced *N*-glycans released from healthy controls (*n* = 5), cystitis biopsies (*n* = 5), non-muscle-invasive bladder cancer (NMIBC; Ta—*n* = 5 and T1 stages—*n* = 5), and muscle-invasive bladder cancer (MIBC; T2–T4; *n* = 8) tumor tissues were analysed by mass spectrometry. In parallel, *N*-glycoproteomics after Galanthus Nivalis Lectin (GNL) enrichment was employed to address the protein carriers of mannose-rich glycans (Ta—*n* = 4; T1—*n* = 4; MIBC—*n* = 4). Spatial analysis and validation of tumor tissues were also attained by immunohistochemistry. Blue square, *N*-acetylglucosamine (GlcNAc); green circle, mannose (Man); yellow circle, galactose (Gal); red triangle, fucose (Fuc); purple diamond, *N*-acetylneuraminic acid (Neu5Ac). (**B**) Relative abundance of individual *N*-glycan structures across the five study groups. Oligomannose structures are represented in blue, neutral complex-type glycans are represented in pink, and sialylated structures are represented in green–brown. Healthy and cystitis tissues display a predominance of complex-type glycans, while Ta tumor tissues show increased levels and a predominance of oligomannose structures. Moreover, T1 and MIBC tumors show a more balanced expression of oligomannose and complex *N*-glycans. Abbreviations: H—Hex, N—HexNAc, F—Fuc, S—Neu5Ac. (**C**) Relative abundance of three major *N*-glycan categories, oligomannose, neutral complex, and sialylated complex, across healthy and diseased bladders. Oligomannose *N*-glycans were significantly elevated in Ta tumor tissues compared to healthy and cystitis tissues, as well as T1 and MIBC tumors. Neutral complex *N*-glycans did not show significant differences across groups. Sialylated complex *N*-glycans were significantly decreased in Ta tumors compared to healthy controls and more aggressive tumors. (**D**) Receiver operating characteristic (ROC) curve evaluating the potential of oligomannose *N*-glycans as possible biomarkers to distinguish cancerous (Ta, T1, and MIBC tumors) from non-cancerous bladder tissues (healthy and cystitis). The area under the curve (AUC) of 0.900, with a sensitivity of 77.78% and specificity of 100%, suggests the strong discriminative power of oligomannose relative levels, quantified by this methodology, as a glycan-based biomarker. Tests used include one-way ANOVA with or without Welch correction. Additionally, Youden Index was used to define the optimal threshold cutoff value. For all analyses, statistical significance was set at *p* < 0.05.

We have characterized the *N*-glycome of healthy urothelium, cystitis (benign bladder inflammation), and tumors at different stages, namely Ta, T1, and muscle-invasive tumors (T2–T4). To achieve this, we employed an innovative strategy in which *N*-glycans were directly released through in situ *N*-deglycosylation using PNGase F on 10 µm tissue sections recovered from paraffin-embedded samples (Figure 1A). The released glycans were then permethylated to enhance ionization efficiency, improve signal intensity, and stabilize sialylated structures during mass spectrometry analysis [20]. This modification increases glycan hydrophobicity, enabling efficient separation on the conventional reverse-phase C18 columns commonly available in proteomics laboratories. Additionally, permethylation preserves the structural features essential for compositional and linkage analysis, facilitating structural elucidation [20].

The study focused on the most abundant glycan species in each case, which accounted for more than 90% of the total glycan content. Collectively, we identified over 20 ions that are consistent with *N*-glycan structures, most of which are expressed in both benign and malignant urothelial conditions (Figure 1B and Supplementary Table S1). The main ions included oligomannosidic structures (H5N2 and H6N2), fucosylated complex structures (H3N4F1 and H4N4F1), and sialylated complex *N*-glycans (H5N4S1, H5N4S2, H5N4F1S1, and H5N4F1S2). On the other hand, some low-abundance (<5% of total *N*-glycans) cancer-specific glycan signatures were identified. Namely, the low-abundance oligomannoses H5N2F1 (40% of Ta cases) and H10N2 (60% of Ta cases) were exclusive to Ta tumors. Additionally, the complex-type structures H6N5S3 (20% of T1 cases, 25% of MIBC cases) and H6N5F1S2 (40% of T1 cases, 63% of MIBC cases) were found in select cancer cases but were absent in the healthy or inflamed urothelium. Interestingly, when focusing on the most abundant glycans, no significant differences were observed between healthy and inflammatory conditions, except for the oligomannose structure H5N2, for which a significant decrease in expression was observed in cystitis biopsies in relation to the healthy tissues (Supplementary Figure S1). However, cancer cases showed an enrichment of oligomannoses, with a striking increase relative to other *N*-glycans in Ta tumors (Figure 1C). This was particularly evident for H6N2, H7N2, H8N2, H9N2, and H10N2 (Supplementary Figure S1). As the disease progressed, this effect was significantly attenuated, accompanied by an apparent reversion toward the glycome profiles observed in healthy and inflammatory conditions (Figure 1B,C). Further investigating this pattern, a receiver operating characteristic (ROC) analysis revealed that the relative abundances of oligomannoses could effectively distinguish cancer from healthy urothelium and inflammation (AUC = 0.90; sensitivity = 78%; specificity = 100%; *p* = 0.0006) (Figure 1D and Supplementary Figure S2). Since our glycomics provides a relative assessment of glycans, we sought to explore the oligomannose levels in cancer samples using the Galanthus Nivalis Lectin (GNL), which primarily targets the terminal α1-3-mannose and α1-6-mannose structures that are abundantly present in oligomannosidic glycans [21,22,23]. GNL lectin binds to glycans on both cancer and immune cells in BLCA and is predominantly detected in the cytoplasm, with no clear distinction at the cell membrane (Supplementary Figure S3). This suggests that these glycans are primarily associated with glycoproteins undergoing *N*-glycan processing, a finding that warrants further validation. We found a trend of increased oligomannose levels in Ta tumors compared to T1 tumors (Supplementary Figure S3). Interestingly, in MIBC, we identified two distinct groups: one expressing higher levels of oligomannoses, suggesting that, despite showing lower relative abundances in relation to less-aggressive tumor stages, a subset of MIBC tumors may retain or even enhance specific oligomannose-rich glycophenotypes. This glycomic heterogeneity in MIBC may reflect the existence of distinct tumor subtypes, potentially characterized by divergent immune microenvironments, metabolic adaptations, or glycan-mediated signaling pathways. Such oligomannose-rich phenotypes may represent a glycan-driven subset of MIBC with a unique biological behavior and therapeutic vulnerabilities.

### 2.2. Oligomannose-Related Glycogenes in BLCA

**Figure 2:**
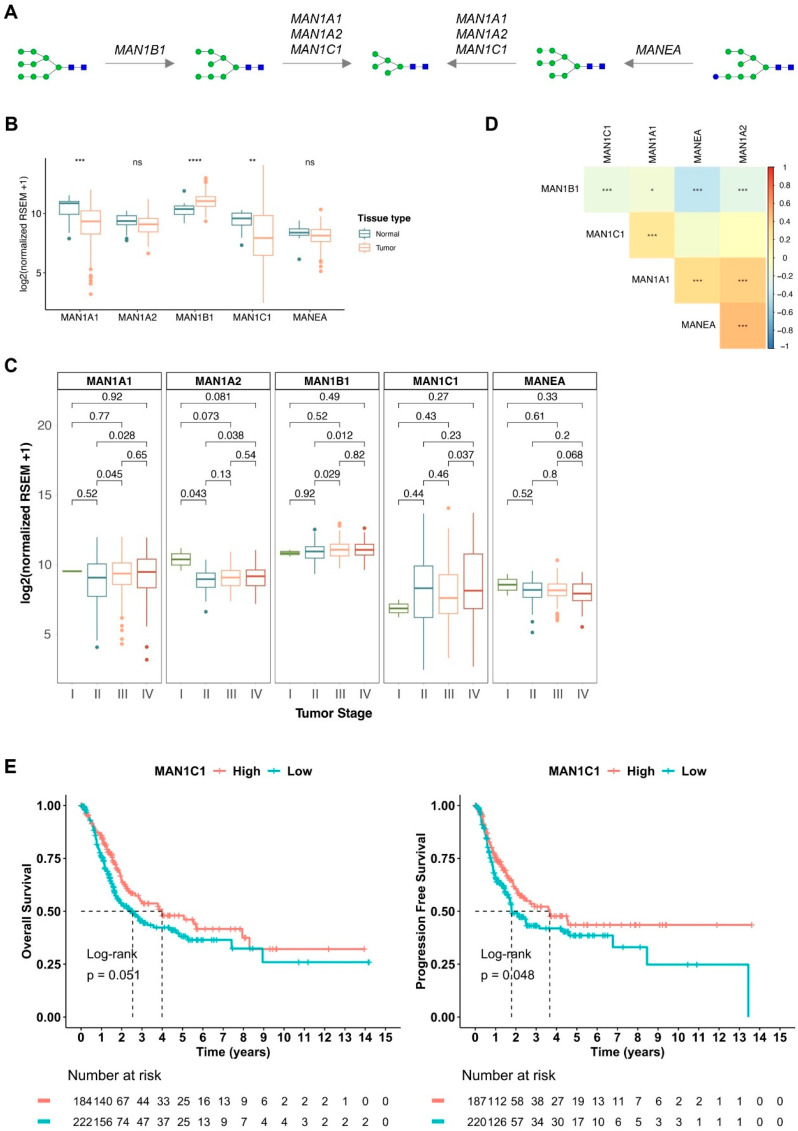
Analysis of mannosidases expression in bladder cancer within the The Cancer Genome Atlas (TCGA) dataset and its relationship to high oligomannose levels associated with the disease and a worse prognosis. (**A**) Schematic representation of oligomannose *N*-glycans biosynthesis pathways. Endoplasmic reticulum (ER) mannosidase 1 (*MAN1B1*) produces Man_8_GlcNAc_2_ *N*-glycan that is further processed in the Golgi by different mannosidases, namely those codified by *MAN1A1*, *MAN1A2*, and *MAN1C1* that remove terminal α1-2 Man, giving origin to smaller oligomannose structures, such as the Man_5_GlcNAc_2_ *N*-glycan. Alternatively, Glc_1_Man_8_GlcNAc_2_ *N*-glycans may reach the Golgi. In this case, a Man_8_GlcNAc_2_ isomer is obtained by the action of Golgi endo-α-mannosidase codified by *MANEA*. Finally, further processing of Man_5_GlcNAc_2_ in the Golgi produces complex *N*-glycans. Blue square, *N*-acetylglucosamine (GlcNAc); green circle, mannose (Man); blue circle, glucose (Glc). (**B**) Expression of mannosidases in bladder tumor and healthy tissues. *MAN1A1* and *MAN1C1* transcript levels were significantly decreased in tumors in relation to healthy bladder. Contrastingly, the expression of *MAN1B1* transcripts was significantly increased in bladder cancer. (**C**) Expression of mannosidases across bladder tumor stages (stage I—*n* = 2; stage II—*n* = 130; stage III—*n* = 139; stage IV—*n* = 134). Transcript levels of the majority of these mannosidases seem not to change with disease progression. Nevertheless, *MAN1A2* was found downregulated in more advanced bladder tumors, and *MAN1B1* levels increased with disease progression. (**D**) Correlogram of mannosidases expression, showing significant positive correlations between *MAN1A1*, *MAN1A2*, *MAN1C1,* and *MANEA* and a negative correlation between *MAN1B1* and all other mannosidases. (**E**) Disease overall survival and progression-free survival curves in relation to *MAN1C1* expression. Low expression of *MAN1C1* transcripts associated with decreased overall survival and progression-free survival. The log-rank test was employed to compare overall survival curves, and univariate analysis was used to evaluate individual prognostic factors. Tests used include nonparametric Wilcoxon test for B and C analyses and Spearman’s correlation for D. For all analyses; statistical significance was set at * *p* < 0.05, ** *p* < 0.01, *** *p* < 0.001, and **** *p* < 0.0001.

We further explored the expressions of several mannosidase-encoding glycogenes, including *MAN1A1*, *MAN1B1*, *MAN1A2*, *MAN1C1*, and *MANEA*, which encode the enzymes responsible for oligomannose trimming during *N*-glycan processing in the endoplasmic reticulum–Golgi, generating Man_5_GlcNAc_2_, the key precursor for complex *N*-glycans (Figure 2A). Due to limited tissue availability, we were unable to directly assess the expression of these mannosidases in our cancer samples. Therefore, we leveraged publicly available transcriptomic data from TCGA, encompassing a healthy urothelium and over 400 bladder cancer cases across different disease stages. We found that *MAN1A1* and *MAN1C1* were significantly downregulated in cancer tissues compared to the healthy urothelium (Figure 2B). Although not statistically significant, *MAN1A2* and *MANEA* also exhibited a decreasing trend, with *MAN1A2* showing a progressive decline with disease progression (Figure 2B,C). On the other hand, MAN1B1 exhibited the opposite behavior, showing increased expression in cancer compared to healthy tissues, with the expression levels rising as the disease progressed. Additionally, we identified a moderate-to-strong positive correlation between *MAN1A1*, *MAN1A2*, *MAN1C1*, and *MANEA*, suggesting co-regulation or functional association in *N*-glycan processing (Figure 2D). Collectively, these findings suggest a reduced capacity to trim high-mannose structures, potentially leading to the accumulation of oligomannoses in BLCA, as suggested by glycomics. Contrastingly, *MAN1B1* showed a negative correlation with all the other mannosidases, further supporting its distinct expression pattern and denoting a different glycophenotype within the tumor microenvironment. The use of the traditional biosynthetic pathway by tumor cells instead of MANEA could also be a cancer-associated mechanism, leading to an increase in Man_8_GlcNAc_2_ levels, as observed in all tumors (Supplementary Figure S1). Finally, when evaluating the potential association between low mannosidase expression—presumed to contribute to the accumulation of oligomannoses—and patient prognosis, only low expression of *MAN1C1* was linked to a trend toward reduced overall survival (OS log-rank *p* = 0.051, DFS log-rank *p* = 0.048) (Figure 2E). Furthermore, in the univariate analysis, *MAN1C1* high expression showed a trend association with better overall survival (*MAN1C1* high: 0.74; *p* = 0.052), as well as decreased disease-free survival, with a hazard ratio also of 0.74 (*p* = 0.049). These results indicate that altered *N*-glycan processing may have functional implications in BLCA progression and patient prognosis, warranting further investigation.

### 2.3. Targeted Glycoproteomics

To advance precise cancer targeting, we further explored the oligomannose-associated glycoproteome. We extracted proteins from formalin-fixed paraffin-embedded (FFPE) tumor tissues previously analyzed for glycomics. Glycoproteins were enriched using GNL and analyzed by nanoLC-HCD-MS/MS.

**Figure 3:**
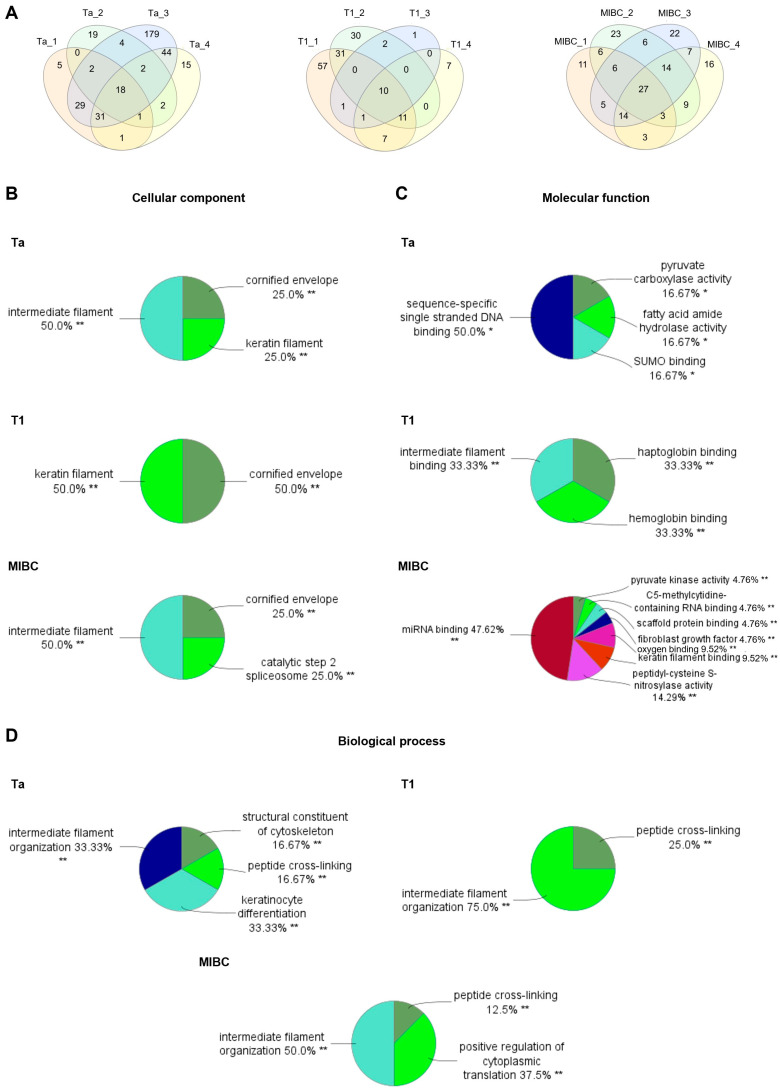
Protein identification and analysis of gene ontology (GO) term annotation of identified proteins across tumor stages. (**A**) Venn diagrams showing the distribution of protein identifications across biological replicates. (**B**) Cell component GO term analysis highlighting the existence of proteins associated with intermediate filaments and cornified envelope in all tumors, independent of tumor stage or grade. (**C**) GO term analysis of molecular functions, revealing that diverse sets of proteins were enriched by GNL in all groups. Moreover, the protein carriers of GNL ligands seem to be distinct and change with disease evolution. (**D**) Analysis of biological functions’ GO term annotation, showing that GNL enrichment selected proteins involved in intermediate filament organization and peptide cross-linking in all tumor stages. Statistical significance was set at * *p* < 0.05, ** *p* < 0.01.

We identified 352 proteins in Ta tumors, including seven with glycopeptide spectral matches (glycoPSMs), confirming glycosylation. In T1 tumors, 158 proteins were detected, six of which exhibited glycoPSMs. In MIBC, 172 proteins were identified, including three with glycoPSMs (Figure 3A and Supplementary Tables S2 and S3). The relatively low number of glycoPSMs observed across tumor stages can be attributed to the lack of a dedicated glycopeptide enrichment step in our workflow. Despite this, the presence of glycoPSMs provides direct evidence of protein glycosylation within these samples.

Gene ontology (GO) enrichment analysis for subcellular localization showed consistent protein localization across tumor stages, with an enrichment in cornified envelope proteins, indicating a shift towards a squamous phenotype (Figure 3B). Additionally, a GO analysis revealed that Ta tumors exhibited enrichment in both keratin filaments and other intermediate filaments, suggesting a balance between epithelial integrity and structural reinforcement. T1 tumors were predominantly enriched for keratin filaments, maintaining an epithelial phenotype, while MIBC tumors showed enrichment in non-keratin intermediate filaments, indicating a shift away from epithelial characteristics, potentially facilitating tumor invasion. Advanced disease exhibited enrichment in components of the catalytic step 2 spliceosome, suggesting a higher prevalence of alternative splicing events generating MIBC-specific isoforms.

A molecular function analysis across tumor stages revealed distinct differences, suggesting a progressive shift in cellular activity during tumor progression (Figure 3C). In Ta tumors, commonly expressed glycoproteins were primarily associated with metabolic and regulatory activities, including pyruvate carboxylase activity, fatty acid amide hydrolase activity, SUMO binding, and sequence-specific single-stranded DNA binding. These findings suggest a reliance on metabolic plasticity and DNA regulatory mechanisms in early-stage tumors. In contrast, T1 tumors exhibited an enrichment of the glycoproteins involved in hemoglobin and haptoglobin binding, as well as intermediate filament binding, indicating a transition towards oxygen transport and cytoskeletal remodeling. This shift may reflect early tumor adaptation to microenvironmental stressors, including hypoxia and increased mechanical strain. In MIBC samples, glycoproteins predominantly exhibited regulatory and structural functions, such as miRNA binding, C5-methylcytidine-containing RNA binding, keratin filament binding, scaffold protein binding, oxygen binding, peptidyl-cysteine S-nitrosylase activity, and fibroblast growth factor binding. These findings suggest that advanced tumors increasingly engage in post-transcriptional regulation, oxidative stress adaptation, and extracellular matrix remodeling, all of which are critical for tumor invasion and progression. Complementary GO term analysis of biological functions further supports these trends. Across all tumor stages, glycoproteins carrying oligomannoses were primarily linked to intermediate filament organization and peptide cross-linking, reinforcing the role of cytoskeletal dynamics in tumor development (Figure 3D). The observed enrichment in keratin filaments in Ta and T1 tumors and intermediate filaments in MIBC tumors aligns with cytoskeletal remodeling patterns, further supporting the progressive structural adaptations seen in tumor progression. Ta tumors were also uniquely associated with keratinocyte differentiation and cytoskeletal structural components, suggesting an early reliance on epithelial integrity. Conversely, MIBC tumors exhibited enrichment in the positive regulation of cytoplasmic translation, reflecting an increased capacity for protein synthesis, which may contribute to rapid tumor growth and adaptation. Overall, our findings highlight tumor-stage-specific glycoproteomes, where oligomannose-modified glycoproteins drive structural integrity, cytoskeletal remodeling, and tumor progression. Early-stage tumors are primarily enriched for the glycoproteins involved in metabolic and DNA-binding processes, whereas intermediate-stage tumors are enriched for glycoproteins driving cytoskeletal remodeling and oxygen transport. Invasive tumors are enriched for glycoproteins involved in post-translational regulation and oxidative stress adaptation, suggesting an active role in tumor plasticity, invasion, and progression. The enrichment of keratin and intermediate filament-associated glycoproteins across stages reinforces the notion that glycosylation influences cytoskeletal remodeling and cell motility. These changes may facilitate epithelial-to-mesenchymal transition, a hallmark of aggressive bladder cancer [24].

**Figure 4:**
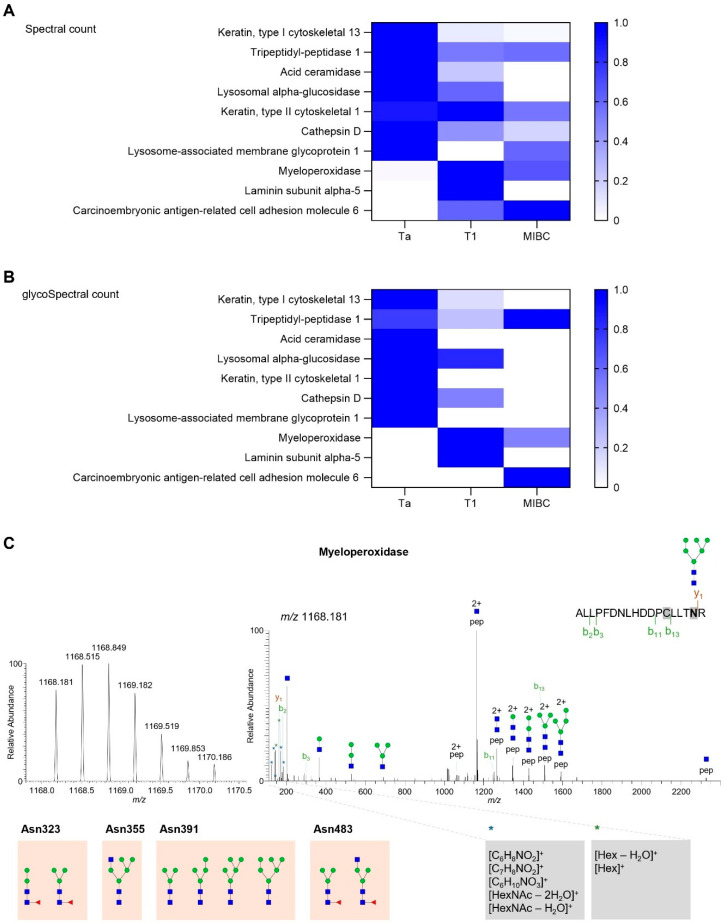
*N*-glycosylated myeloperoxidase was identified exclusively on T1 and MIBC tumors. (**A**,**B**) Heatmaps showing the distribution of proteins identified with confirmed glycosites (glycoPSM) across tumor stages. The color key indicates the normalized razor spectral (**A**) and glycospectral (**B**) counts per detected protein across tumor stages. GNL favored the enrichment of proteins with higher expression in early-stage tumors (Ta). However, while some of the identified proteins (e.g., tripeptidyl-peptidase 1) were identified in all disease groups, others seem to be exclusive to early-stage tumors (e.g., acid ceramidase and lysosomal alpha-glucosidase). Contrastingly, myeloperoxidase and carcinoembryonic antigen-related cell adhesion molecule 6 were more associated with disease progression and aggressiveness. (**C**) MS and MS/MS spectra for a myeloperoxidase glycopeptide covering Asn391 occupied with an oligomannose *N*-glycan. MS/MS spectrum shows b/y peptide fragmentation, Y-type ions constituted by the peptide and a portion of glycan structure, and glycan oxonium ions. Namely, product ions from HexNAc and Hex fragmentation identified in the MS/MS spectrum appear with an asterisk and are highlighted in grey boxes. Orange boxes highlight the myeloperoxidase glycosites identified in this study and the corresponding glycan compositions. Blue square, *N*-acetylglucosamine (GlcNAc); green circle, mannose (Man); red triangle, fucose (Fuc).

We further utilized MSFragger to annotate glycopeptides from GNL-enriched glycoproteins. We confidently identified ten glycoproteins with glycopeptides consistent with the presence of GNL ligands. Notably, acid ceramidase and lysosomal alpha-glucosidase were exclusive to Ta and T1 tumors, suggesting NMIBC-specific signatures (Figure 4A,B and Supplementary Table S4). Acid ceramidase, for instance, is implicated in sphingolipid metabolism and has been associated with chemoresistance and tumor survival mechanisms in various cancers [25,26,27]. Its stage-specific glycosylation in early BLCA may enhance its stability, therefore contributing to its subcellular localization and influencing tumor cell viability. Moreover, Keratin type I cytoskeletal 13, Cathepsin D, and Keratin type II cytoskeletal 1 were predominantly detected in Ta and T1 tumors, presenting lower expression in MIBC when detected. Also, the presence of glycosylation on these proteins was only detected in Ta and/or T1 tumors. In contrast, myeloperoxidase (MPO) and carcinoembryonic antigen-related cell adhesion molecule 6 (CEACAM6) were primarily detected in T1 and MIBC, indicating a potential connection with high-grade disease and progression. Namely, CEACAM6 is known to promote cell adhesion, invasion, and resistance to anoikis, supporting its potential role in disease progression [28]. Additionally, laminin subunit alpha-5 was uniquely identified in T1 tumors, suggesting a role in early tumor invasion and basement membrane remodeling. These findings suggest that glycosylation status may modulate the oncogenic functions of these carriers. Based on both peptide-spectra matches (PSMs) and glycoPSMs, MPO emerged as the most abundant glycoprotein, particularly in more aggressive stages of bladder cancer (T1 and MIBC). In MPO, oligomannose structures were exclusively detected at Asn391, including Man_5_GlcNAc_2_ (H5N2) and Man_6_GlcNAc_2_ (H6N2), as supported by the annotated product ion spectrum shown in Figure 4C. Additionally, we identified paucimannose-type glycans at multiple glycosylation sites: Asn323 (Man_2_GlcNAc_2_Fuc_1_; Man_3_GlcNAc_2_Fuc_1_), Asn483 (Man_3_GlcNAc_2_Fuc_1_), and Asn391 (Man_3_GlcNAc_2_; Man_4_GlcNAc_2_) (Supplementary Table S3). Although these structures represent low-abundance *N*-glycans in bladder cancer (<10% of total glycan content in our bladder samples, and therefore not quantified among the most abundant ions), they are notable for their high affinity to GNL, potentially due to exposed mannose residues, and a deeper investigation into their biological relevance and biosynthetic origins is needed. Furthermore, we detected hybrid *N*-glycans, including GlcNAc_1_Man_5_GlcNAc_2_ (H5N3) at Asn355 and GlcNAc_1_Man_3_GlcNAc_2_Fuc_1_ (H3N3F1) at Asn483, highlighting the presence of glycan heterogeneity across different glycosylation sites. This suggests the coexistence of distinct glycosylation types in MPO (oligomannose, paucimannose, and hybrid, as well as core-fucosylated structures) and some degree of glycosite occupancy heterogeneity, which warrants further investigation in larger cohorts. Such diversity may reflect localized differences in glycan processing or accessibility during biosynthesis and could have functional implications in the context of tumor progression, which warrants future investigations. Importantly, hyper-truncated or oligomannosylated glycoforms of MPO have been shown to alter their enzymatic activity and interactions with immune modulators such as ceruloplasmin [29]. In the context of bladder cancer, this atypical glycosylation may modulate redox signaling or contribute to immune escape through an engagement of mannose-recognizing lectins, such as DC-SIGN or the macrophage mannose receptor [30,31].

In contrast, acid ceramidase was predominantly expressed in Ta tumors and also confirmed to carry oligomannoses, as demonstrated by the MS/MS spectrum confirming *N*-glycosylation of Asn259 with Man_6_GlcNAc_2_ (Supplementary Figure S4). Overall, despite its explorative nature, this dataset provides proof of concept that BLCA harbors a stage-dependent oligomannosidic-associated glycoproteome.

### 2.4. Myeloperoxidase Expression in BLCA

**Figure 5:**
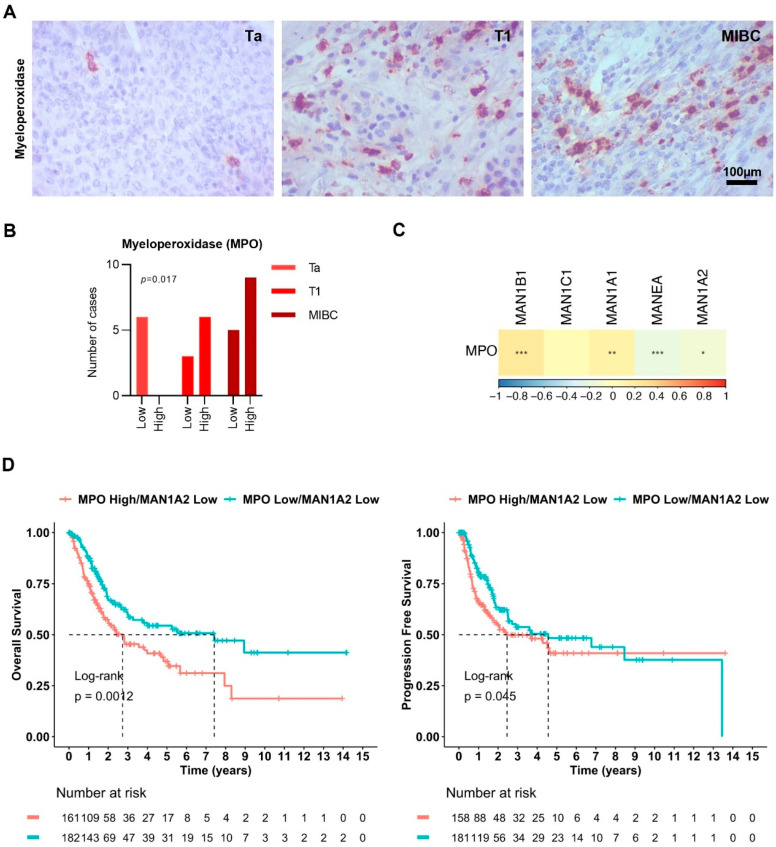
Immunohistochemistry cross-validation demonstrates the association of myeloperoxidase (MPO) carrying oligomannose *N*-glycans with more aggressive tumors. (**A**) Myeloperoxidase expression was mainly observed in the cytoplasm of immune cells. (**B**) Myeloperoxidase is highly expressed in most aggressive tumors (T1 high-grade and MIBC tumors), presenting only low expression in a small group of Ta tumors. (**C**) TCGA dataset analysis of the correlation between *MPO* transcript expression and the expression of mannosidases. *MPO* is positively correlated with *MAN1A1* and *MAN1B1*, while an inverse correlation was observed between *MPO* expression and *MAN1A2* and *MANEA*. (**D**) Disease overall survival and progression-free survival curves for patients with low *MAN1A2* expression levels as a function of *MPO* expression. Tumors exhibiting high *MPO* transcript levels and low *MAN1A2* transcript levels presented statistically significant lower overall survival and progression-free survival compared with tumors presenting low expression of *MPO* and *MAN1A2* transcripts. The log-rank test was employed to compare survival curves, and univariate analysis was employed to evaluate individual prognostic factors. Tests used include Chi-square test for analysis presented in B and Spearman’s correlation for C. For all analyses, statistical significance was set at * *p* < 0.05, ** *p* < 0.01, and *** *p* < 0.001.

We then sought to validate the association of MPO with tumor aggressiveness suggested by glycoproteomics. Using immunohistochemistry, an orthogonal, spatially resolved validation, we confirmed that MPO is overexpressed in advanced tumors (T1 and MIBC; Figure 5A,B), consistent with previous observations from mass spectrometry. Notably, its expression was primarily localized to the immune component of the tumor, reinforcing its role in the tumor microenvironment. MPO staining was observed in the cytoplasm, consistent with its well-characterized subcellular localization in azurophilic granules of granulocytes and other immune cells [32]. This aligns with its function as a lysosomal enzyme involved in antimicrobial activity and oxidative stress regulation, which warrants confirmation in BLCA. Importantly, MPO co-localized with GNL staining in the same tissue areas, suggesting the presence of oligomannose-type glycans, consistent with previous glycoproteomics findings. Collectively, these findings reinforced the presence of MPO-carrying oligomannoses in advanced disease.

To explore this further, we interrogated TCGA transcriptomic data and found that *MPO* significantly correlates with *MAN1B1*. Contrastingly, *MPO* showed significant inverse correlation with *MAN1A2* and *MANEA* expressions, alongside a positive correlation with *MAN1A1* (Figure 5C). MANEA, localized in the cis/medial Golgi and ER–Golgi intermediate compartment, provides an alternative pathway for trimming mannose residues on ER-escaped glycoproteins that are incompletely processed [33]. The reduced expression of this enzyme likely demonstrates that the accumulation of oligomannose structures on MPO in bladder cancer derives from complete processing in the ER, without the participation of the alternative pathway involving MANEA. Interestingly, the Golgi-resident enzymes encoded by *MAN1A1* and *MAN1A2*, particularly important for trimming α1,2-linked mannose residues on the same steps of early *N*-glycan processing [34], showed different expression correlations with *MPO* (positive and negative correlation, respectively). This is likely related to the presence of different degrees of mannose trimming on observed oligomannose *N*-glycans, carrying from nine to five Man. A future in-depth investigation will be necessary to better understand the impact of mannosidases in each step of *N*-glycan biosynthesis and, consequently, in the glycoprofile of bladder tumors. Clinically, this altered glycosylation landscape may have prognostic implications. We found that *MPO*-overexpressing tumors with low *MAN1A2* expression exhibited significantly reduced overall survival (*p* = 0.0012; hazard ratio—HR for MPO^high^/MAN1A2^low^: 1.73, *p* = 0.001) and progression-free survival (*p* = 0.045; HR = 1.42, *p* = 0.046) compared to tumors with low *MPO* expression (Figure 5D). A similar association was observed for *MANEA*, where low expression was linked to worse overall survival outcomes (Supplementary Figure S5). Collectively, these findings support a model in which *MPO* overexpression in advanced BLCA is linked to impaired *N*-glycan maturation and immune infiltration, with potential consequences for glycoprotein function and tumor progression, warranting in-depth investigations. The combined analysis of MPO and mannosidase expression profiles could serve as a stratification tool to identify patients with poor prognosis, offering a basis for the development of glyco-biomarker panels. Such profiles may aid in refining risk models, guiding treatment decisions, and informing future studies on therapeutic targeting of glycosylation pathways.

## 3. Discussion

It has long been established that the urothelium glycome undergoes significant remodeling during cancer progression, with early studies linking the loss of ABO blood group determinants in secretor individuals to poorer prognosis [35]. Since then, we have provided comprehensive evidence that the glycome of more aggressive tumors becomes enriched in immature *O*-glycans, such as the Tn and STn antigens, which, due to their cancer-specific nature, hold potential for patient stratification and therapeutic development [14,36]. However, the *N*-glycome remains poorly characterized and largely unexplored.

Our study systematically investigates the *N*-glycomic landscape of BLCA across multiple disease states, from a normal urothelium and cystitis to non-invasive (Ta), early invasive (T1), and MIBC. The primary focus was placed on the most prominently expressed glycosylation signatures, as these are more likely to exert dominant biological effects or serve as potential drivers of phenotype. While lower-abundance glycans accounted for approximately 10% of the glycome, they were excluded from the core analysis due to their limited representation. However, their potential functional relevance, particularly in modulating subtle or context-dependent processes, should not be overlooked and warrants future investigation. Nevertheless, unlike prior glycomic analyses that predominantly address more aggressive tumors or tumor-adjacent normal tissues, our approach captures dynamic glycosylation shifts across disease progression, allowing us to pinpoint key transition points [37,38]. This is particularly relevant for the clinical management of NMIBC, where accurate risk stratification remains a major challenge. Incorporating cystitis samples, we also address a significant limitation of prior research. Chronic inflammation is a well-established driver of bladder carcinogenesis [39], yet the glycosylation pattern of bladder inflammation remains, to our knowledge, mostly unknown. Interestingly, we could not identify major relevant alterations in the *N*-glycome of inflamed urothelial tissues compared to healthy urothelium. This contrasts with previous studies in other inflammatory contexts, where glycosylation changes are frequently observed. Namely, shifts in serum glycosylation have been well-documented in conditions such as rheumatoid arthritis [40], inflammatory bowel disease [41], and chronic infections [42]. Moreover, the increased expression of glycosyltransferases, involved in the synthesis of sialyl Lewis terminal epitopes, branching, and/or in outer-arm/core fucosylation of *N*-glycans, has been correlated with elevated inflammatory cytokines, particularly IL-6, namely in liver inflammation and cancer [43]. Moreover, the desialylation of glycoproteins, including IgG, has been linked to pro-inflammatory immune responses, exacerbating chronic inflammatory states [44]. These systemic alterations suggest a strong link between inflammation and glycosylation, yet our findings indicate that such changes may not be universally applicable across all tissues. Our findings suggest that the urothelium exhibits a tissue-specific glycosylation response to inflammation, potentially governed by local microenvironmental cues and distinct epithelial immune dynamics. It is also possible that the inflammatory microenvironment of the urothelium induces changes that are either too subtle to be captured by our current analytical approach or are more prominent in specific glycoproteins rather than at the global *N*-glycome level. Thus, future studies incorporating spatial glycoproteomics or single-cell profiling may be necessary to uncover these fine layers of glyco-immune modulation. Additionally, the nature and chronic inflammation could influence the extent of glycosylation remodeling, with acute versus chronic inflammation exerting distinct effects. Experimental models of inflammation could further delineate these mechanisms. Future studies should also explore specific glycoprotein targets, focusing on potential alterations in key glycan structures that could contribute to immune modulation within the urothelial microenvironment.

Nevertheless, our analysis enables a refined distinction between healthy and inflammation-driven glycomic remodeling and cancer-specific glycosylation alterations, enhancing the specificity of glycan-based biomarkers. A central finding of this study is the identification of a progressive shift in oligomannosidic *N*-glycans during disease progression. Ta tumors displayed a striking enrichment in the oligomannoses compared to normal and inflammatory tissues, a phenomenon that became attenuated in later stages, with MIBC tumors displaying heterogeneous oligomannose expression. This trend appears to reflect impaired *N*-glycan processing, potentially linked to enzymatic trimming deficits in the ER and Golgi apparatus. However, this proposed mechanism remains to be experimentally confirmed. ROC analysis confirmed that the relative abundance of oligomannoses could distinguish BLCA from normal and inflamed urothelia with high sensitivity and specificity, warranting validation in larger cohorts to assess their potential as diagnostic biomarkers. Notably, oligomannose enrichment correlated with the transcriptomic downregulation of α1,2-mannosidases (e.g., *MAN1A1*, *MAN1C1*), supporting a possible role for ER/Golgi maturation bottlenecks. However, direct causality remains to be established. These findings are particularly relevant in the context of disease management, as they may represent an early glycosylation abnormality linked to initial tumorigenic transformation. Clinically, the ability to distinguish early-stage cancer lesions from the reactive urothelium or inflammatory conditions such as cystitis is critical, as both can present with similar histopathological and symptomatic features [3,45]. The presence of oligomannose-enriched glycoproteins may, therefore, serve as a molecular discriminator in equivocal cases, aiding in diagnosis and reducing the risk of misclassification. Furthermore, as these glycans appear in early-stage disease, they raise an opportunity for bladder cancer detection screening programs, which currently do not exist. Nevertheless, further investigation is warranted for future diagnostic development. Further studies may also inform on the potential of addressing this glycan signature during cystoscopy through the use of glycan-specific imaging agents. This approach may improve the detection of flat or subtle Ta lesions that are often missed with standard white-light visualization [3]. The integration of glycan-targeted tools into cystoscopic evaluation could enhance diagnostic sensitivity, facilitate more accurate real-time assessments, and ultimately contribute to better clinical decision-making in early bladder cancer. Considering these preliminary observations, future studies should aim to validate these findings in larger patient cohorts to ensure robustness and clinical relevance. Particular attention should also be given to carcinoma in situ (CIS), a flat, high-grade lesion that presents a significant diagnostic and management challenge in bladder cancer. As CIS is often difficult to detect with conventional imaging and histopathological evaluation [3], investigating whether oligomannose signatures or related glycosylation patterns are also present in CIS in future studies could provide critical insights and potentially expand the utility of glycan-based markers in clinical practice. These findings also suggest potential for non-invasive detection through urine-based assays. Such approaches could aid in early diagnosis and surveillance, potentially reducing the need for invasive cystoscopy. Notably, to complement the glycomics data with spatial and semi-quantitative information, we employed GNL lectin binding, which selectively recognizes terminal mannose residues characteristic of oligomannose-type *N*-glycans [21,22,23]. Using this approach, we observed decreased GNL binding in T1 compared to Ta tumors. In MIBC, however, the GNL staining patterns were more heterogeneous: while a reduction in mannose-rich *N*-glycan signal was notable in many cases, a distinct subset of tumors retained or even enhanced oligomannose-rich glycophenotypes. This intra-stage heterogeneity may reflect divergent tumor subtypes or metabolic rewiring and is compatible with the notion that Golgi dysfunction influences glycan maturation, although this remains to be validated in functional models.

Consistent with our observation of elevated oligomannosylation in Ta-stage bladder tumors, immature oligomannose structures are a common glycosylation feature in several cancer types. These under-processed glycans have been identified in cancer cell lines from at least nine tumor types, including bladder [46], breast [47], and colorectal [48], and confirmed in tumor tissues from the skin [49], colorectal [50], and lung [51]. In contrast, cancers such as gastric [52], hepatocellular [53], kidney [54], pancreatic [55], and thyroid [56] show unchanged or reduced oligomannosylation, highlighting the tissue-specific nature of glycan remodeling. Still, our findings support the idea that oligomannose enrichment, while not universal, could represent a relevant and potentially functional hallmark of early bladder cancer, possibly with implications for tumor biology and immune interactions that merit further investigation. Additionally, increased expression of oligomannoses in cancer has been linked to reduced expression or activity of α1,2-mannosidases, such as *MAN1A1*, *MAN1A2*, and *MAN1C1* [10]. Our findings reinforce this association by showing that the coordinated downregulation of multiple mannosidases is a prominent feature of bladder cancer and is associated with tumor aggressiveness. Transcriptomic correlation analysis further showed that low *MAN1C1* expression trends with poorer survival, suggesting functional consequences of impaired glycan trimming. Taken together, these observations support a mechanistic framework in which ER and Golgi dysfunction may underlie oligomannose accumulation in BLCA. However, functional demonstration of this pathway is still needed. Also, although the present study does not directly examine the functional role of oligomannoses in bladder cancer, prior research demonstrates that these structures can be recognized by innate immune receptors such as mannose-binding lectins (e.g., DC-SIGN and the macrophage mannose receptor) [57,58,59], raising the possibility that oligomannosylation modulates tumor–immune interactions, either enhancing immunosurveillance or promoting immune evasion, depending on the context. In some settings, elevated oligomannose levels have also been linked to increased tumor invasiveness, altered endocytosis, and loss of epithelial polarity [60,61,62]. As with other glycosylation patterns, the biological effects of oligomannosylation are likely context-dependent and vary across cancer types.

To extend these findings, we explored the oligomannose-associated glycoproteome using GNL enrichment and glycoproteomics. Our analysis identified distinct glycoproteins carrying oligomannoses across tumor stages, suggesting stage-specific glycosylation patterns. Notably, MPO and CEACAM6 were predominantly detected in T1 and MIBC tumors, implicating their involvement in high-grade disease and potential progression. In contrast, acid ceramidase was exclusively glycosylated in Ta tumors, highlighting its potential role in early-stage disease. These findings underscore the utility of glycoproteomics in identifying stage-dependent glycosylation signatures that could improve bladder cancer classification and treatment strategies. A functional enrichment analysis further revealed that glycoproteins carrying oligomannoses exhibit distinct cellular functions at different tumor stages. Ta tumors were enriched for metabolic and DNA-binding glycoproteins, suggesting a reliance on metabolic plasticity and transcriptional regulation in early-stage disease. In contrast, T1 tumors displayed enrichment for proteins linked to cytoskeletal remodeling. MIBC tumors, on the other hand, were predominantly enriched with the glycoproteins involved in post-transcriptional regulation, oxidative stress adaptation, and extracellular matrix remodeling, all of which are critical for tumor invasion and progression. These patterns suggest a shift in glycoproteomic function accompanying tumor evolution, potentially influenced by the persistence of oligomannosylation. Our findings also highlight the potential role of MPO in bladder cancer, as its expression correlated with oligomannose-rich glycosylation and was predominantly localized to the immune component of the tumor microenvironment. This implies that MPO glycosylation could shape local immune responses or redox balance, though its functional relevance remains to be defined and should be addressed in future mechanistic work. Interestingly, our observation of MPO as a major oligomannose- and paucimannose-bearing glycoprotein in advanced BLCA stages aligns with recent insights from neutrophil glycobiology. Specifically, studies profiling the neutrophil glycoproteome have shown that MPO harbors organelle-specific, site-selective *N*-glycans, particularly truncated and paucimannosidic structures, primarily located in the azurophilic granules formed during early granulopoiesis [29,32]. Furthermore, a functional analysis revealed that hyper-truncated glycans at MPO glycosites can modulate their enzymatic function and interactions with immune regulators such as ceruloplasmin [29,63]. These insights are consistent with our findings and support a plausible role for MPO glycosylation in immune modulation, though direct demonstration in the context of bladder cancer is still required. Furthermore, additional transcriptomic analysis addressing the expression of α-mannosidases II, such as *MAN2A1* or *MAN2A2*, as well as Golgi hexosaminidase, which are involved in the synthesis of paucimannoses, may help to elucidate the mechanism underlying the expression of these small *N*-glycans.

Despite the exploratory and descriptive nature of the work, this study establishes a foundational framework towards glycan-based precision oncology, suggesting new avenues for mechanistic investigation and diagnostic innovation in bladder cancer. Several limitations must be acknowledged, including the relatively small cohort size, possible artifacts introduced by FFPE preservation, and the limited depth of glycoproteomic coverage. While transcriptomic data lent contextual support, dedicated functional studies are essential to substantiate the proposed mechanisms. Strengthening these findings will require larger, well-annotated patient cohorts, additional orthogonal validation, and complementary in vitro or in vivo models. Moving forward, research should prioritize spatial and single-cell glycoproteomic profiling, as well as single-cell RNA seq, to investigate the intra-tumoral heterogeneity and the potential immunomodulatory roles of oligomannosylated proteins, such as MPO and CEACAM6, and develop non-invasive, urine-based glycan biomarkers to support disease detection and surveillance. Together, these efforts will be central to translating oligomannose-linked signatures into clinically meaningful tools.

## 4. Materials and Methods

### 4.1. Bladder Tissues

This study regarding biomarker discovery was performed retrospectively in a patient sample set of formalin-fixed paraffin-embedded (FFPE) bladder tumors obtained from archived paraffin blocks at the IPO-Porto, Portugal. Patients were admitted and treated at IPO-Porto between 2000 and 2016. All procedures were performed under the patient’s informed consent and after approval of the IPO-Porto ethics committee (reference glycoBodies 86/017).

### 4.2. N-Glycan Release and Processing

*N*-glycans were extracted and processed as described previously [36]. Briefly, FFPE tissues (10 µm sections) were deparaffinized and rehydrated by immersing the FFPE tissues for 7 min in xylene twice, 5 min in 100% ethanol twice, 5 min in 96% ethanol twice, 5 min in 70% ethanol, and 5 min in water. Then, the FFPE tissues were subjected to heat-induced antigen retrieval for 20 min, using pre-warmed 1:100 citrate-based antigen unmasking solution (Vector Laboratories, Newark, CA, USA) and denatured and reduced by incubation with a 150 µL denaturation mix (145 µL of 8 M GuHCl and 5 µL of 200 mM dithiothreitol) at 60 °C for 30 min. After that, *N*-glycans were enzymatically released on a tissue slide with PNGase F from *Elizabethkingia miricola* (1 U/10 µg protein at 37 °C overnight; Promega, V4831, Madison, WI, USA). The glycosylamine form of released *N*-glycans was hydrolyzed on glass tubes by incubation for 1 h at RT, with 25 µL of 100 mM ammonium acetate at pH 5, followed by reduction with 20 µL of 1 M NaBH_4_ in 50 mM KOH (3 h at 50 °C). The reaction was quenched by adding glacial acetic acid. Before analysis by reverse phase nanoLC-ESI-MS/MS, *N*-glycan samples were desalted using a cation-exchange resin (AG 50W-X8; Bio-Rad, Hercules, CA, USA), and reduced *N*-glycans were permethylated. Specifically, dried *N*-glycans were resuspended in 500 µL dimethyl sulfoxide, followed by incubation with sodium hydroxide for 30 min under stirring conditions. Methylation of all hydroxyl, amine, and carboxyl groups occurred by incubation with 300 µL methyl iodide for 30 min. A second addition of 100 µL methyl iodide may be considered. Permethylated *N*-glycans were extracted by chloroform–water separation and dried in a rotational vacuum concentrator.

### 4.3. N-Glycan Mass Spectra Acquisition

*N*-glycan data acquisition was performed on a 3000 Ultimate nano-LC (Thermo Fisher Scientific, Waltham, MA, USA) coupled to a QExactive mass spectrometer (Thermo Fisher Scientific, Waltham, MA, USA) equipped with a nano-electrospray ion source (EASY-Spray source; Thermo Fisher Scientific, Waltham, MA, USA). Eluent A was aqueous formic acid (0.2%), and eluent B was formic acid (0.2%) in acetonitrile. The glycan samples (20 µL injection volume equivalent to 10% of *N*-glycans detached from each tissue section) were loaded on a trapping column (C18 PepMap 100, 5 μm particle size; Thermo Fisher Scientific, Waltham, MA, USA) and washed with an isocratic flux of 90% eluent A and 10% eluent B at a flow rate of 30 μL/min. After 3 min, the flux was redirected to the analytical column (EASY-Spray C18 PepMap, 100 Å, 150 mm × 75 μm ID and 3 μm particle size) at a flow rate of 0.3 μL/min. The column temperature was set at 35 °C. Permethylated glycan separation occurred using a multistep linear gradient to obtain 10% eluent B at 10 min, 38% eluent B at 20 min, 50% eluent B at 55 min, and 90% eluent B at 65 min. The column was maintained at 90% eluent B for 10 min before re-equilibration at 10% eluent B. The mass spectrometer was operated in the positive ion mode, with an *m*/*z* range from 500 to 4000 with 120k resolution (Full MS), a spray voltage of 1.9 kV, and a transfer capillary temperature of 275 °C. Tandem MS (MS/MS) data were acquired using a data-dependent method with dynamic exclusion of 5.0 s at a 30k resolution. The top 15 most intense ions were selected for higher energy collisional dissociation (HCD) using 30% normalized collision energy (nce) and an isolation window of 4.0 *m*/*z*. Re-analysis of some samples was also performed with an nce of 10% to enable appropriate *N*-glycoprofiling, since an nce equal to 30 does not provide sufficient information.

### 4.4. N-Glycan Data Analysis

Data were recorded with Thermo Scientific Xcalibur software version 3.0 (Thermo Fischer Scientific, Waltham, MA, USA), considering Gaussian smoothing, mass tolerance of 10 ppm, 4 decimals for mass precision, and default baseline subtraction. The most abundant ions based on their intensity in the average spectrum were investigated, and those with matching known *N*-glycan structures were considered. Moreover, corresponding ions with other charges were also investigated and quantified. This generated a final list that was searched for all samples. Only permethylated reduced *N*-glycan structures containing Gal, Man, GlcNAc, Fuc, and/or Neu5Ac were considered. Chromatography retention times, monoisotopic *m*/*z* values, and MS/MS assisted *N*-glycans assignment validation. Moreover, all assigned [M + H]^+^ are reported in GlycoMod and GlyConnect. The relative abundance resulted from the sum of the extracted ion chromatogram areas for each glycan structure, considering ions with charge z = 1, z = 2, and z = 3, when applicable, in relation to the sum of chromatographic areas of all identified glycans. For relative quantification, isomeric/isobaric *N*-glycan structures were not distinguished. GlycoWorkBench v2.1 (San Jose, CA, USA) [64] was used to generate glycan cartoons. The data analysis regarding *N*-glycan retention time and characterization (ms2 annotation) is available in Supplementary File S1.

### 4.5. N-Glycoprotein Enrichment and Processing

Proteins were extracted from FFPE tissues (10 µm sections) using the QProteome FFPE tissue kit (Qiagen, Hilden, Germany). Specifically, tissue sections were incubated with 500 µL heptane for 1 h at RT, followed by the addition of methanol and centrifugation for 2 min at 9000× *g* to remove the paraffin. This step was performed twice. After solvent evaporation, the samples were incubated with 188 µL EBX Plus buffer with 12 µL β-mercaptoethanol on ice for 5 min, followed by incubation at 100 °C for 20 min and then at 80 °C for 2 h. Proteins were extracted by 15 min centrifugation at 14,000× *g*, followed by acetone precipitation, and 200 µg of total protein extracts were resuspended in GNL buffer (10 mM HEPES, pH 7.5, 0.15 M NaCl, 0.1 mM CaCl_2_) and loaded on agarose-bound GNL for enrichment (Vector Laboratories, Newark, CA, USA). After washing the column 10 times with GNL buffer, the glycoproteins were eluted with 3 × 100 µL 3% acetic acid and dried in a rotational vacuum concentrator. GNL-enriched glycoproteins were resuspended in 50 mM ammonium bicarbonate and incubated at 80 °C for 10 min. Subsequently, disulfide bonds were reduced with 5 mM dithiothreitol (Sigma-Aldrich, St. Louis, MO, USA) at 60 °C for 30 min, and cysteine residues were alkylated with 10 mM iodoacetamide (Sigma-Aldrich, St. Louis, MO, USA) for 30 min in the dark. Glycoprotein digestion occurred overnight at 37 °C with trypsin (Promega, Madison, WI, USA), followed by treatment with trifluoroacetic acid and solution clearing by centrifugation.

### 4.6. N-Glycoprotein Mass Spectra Acquisition

Dried samples were resuspended in 30 µL of an aqueous solution containing 2% acetonitrile 0.2% formic acid, and 10 µL were injected. The mass spectrometry analysis was performed by nanoLC-MS/MS using a Vanquish neoUHPLC nano-LC coupled to a QExactive Plus mass spectrometer (Thermo Fisher Scientific, Waltham, MA, USA). Glycopeptides were separated in the analytical column (EASY-Spray C18 PepMap, 100 Å, 150 mm × 75 μm ID, and 3 μm particle size) at a flow rate of 0.25 μL/min, using a linear gradient of 12–46% eluent B over 50 min. Column wash and re-equilibration were warranted before the following injection. The column temperature was set at 35 °C. The mass spectrometer was operated in the positive ion mode, with an *m*/*z* range from 300 to 2000, a spray voltage of 1.9 kV, and a transfer capillary temperature of 275 °C. Q-Exactive Plus settings were full-scan resolution 140k, automatic gain control (AGC) of 3 × 10^6^, and a maximum injection time of 200 ms. The top 15 peaks were selected for HCD fragmentation, using the following settings: fragment scan resolution 17,500, fragment scan fixed first mass at 110 *m*/*z*, AGC target of 1 × 10^5^, maximum injection time 100 ms, and isolation window 4.0 *m*/*z*. The data-dependent parameters were as follows: minimum AGC target 7 × 10^3^, exclusion of charge unassigned, 1, and >8, peptide match preferred, exclude isotopes on, and dynamic exclusion of 30 s. Two MS runs were performed, differing in the nce applied: nce of 20% and stepped nce of 35 with 2 steps.

### 4.7. N-Glycoprotein Data Analysis

*N*-glycoprotein identification was performed using the MSFragger version 4.1 (Ann Arbor, MI, USA) within FragPipe interface v22.0 (Ann Arbor, MI, USA) and the default workflow glyco-N-HCD. Specifically, HCD-MS raw data were searched against the reviewed UniProt KB human proteins (20,412 entries; accessed on November 2024), including contaminants. Trypsin was defined as the enzyme in protein digestion, enabling 3 missed cleavages, a peptide length between 7 and 50 amino acids, and a peptide mass range of 400–5000. For peak matching, a precursor mass tolerance of 20 ppm and a fragment mass tolerance of 20 ppm were considered, enabling mass calibration and parameter optimization. Methionine oxidation (+15.9949 Da) was considered a variable modification, and carbamidomethylcysteine (+57.02146 Da) was considered in fixed modifications. Meanwhile, the labile modification search mode was set to no nglycan. Moreover, the maximum fragment charge was increased to 4. PSM validation, with PeptideProphet, protein inference, and an FDR filter equal to 0.01, was enabled for validation. Finally, PTM-Shepherd was enabled with the default settings for glyco search. The large *N*-glycan database containing 708 entries was enabled, and a glycan FDR of 0.01 was considered. Curated protein results only include human proteins with a protein probability greater than or equal to 0.9, containing at least 2 total peptides and 1 unique peptide. In parallel, the curated PSMs list contains PSMs from human proteins with a probability greater than or equal to 0.9, while for glyco PSMs (gPSMs), the following criteria were also considered: a positive glycan score and a glycan q value less than or equal to 0.01. Examples of glycoPSMs for myeloperoxidase, considering the different glycosites and glycan compositions, as well as myeloperoxidase’s PSMs for all samples, were included in Supplementary File S1.

### 4.8. N-Glycan Spatial Analysis

For the spatial evaluation of GNL ligands expression, FFPE tissue sections of 3 mm were deparaffinized and rehydrated by immersion in xylene (2 × 7 min), 100% ethanol (2 × 5 min), 96% ethanol (2 × 5 min), 70% ethanol (5 min), and water (5 min). Then, tissues were subjected to heat-induced antigen retrieval by incubation in pre-warmed 1:100 citrate-based antigen unmasking solution (Vector Laboratories, Newark, CA, USA) for 20 min. To block endogenous peroxidases, tissue sections were then exposed to 3–4% hydrogen peroxide for 5 min (Leica, Wetzlar, Germany). After incubation with 5% BSA in phosphate-buffered saline (PBS) for 1 h at RT, GNL ligands were detected using 2.5 µg/mL biotinylated GNL (overnight at 4 °C; B-1245-2; Vector Laboratories, Newark, CA, USA), followed by incubation, 30 min at RT, with the Vectastain ABC kit (Vector Laboratories, Newark, CA, USA) and 3, 3′-diaminobenzidine for 5 min. Additionally, the nuclei were stained with hematoxylin. A semi-quantitative analysis of the slides was performed, evaluating the percentage of positive cells (extension) and the intensity of the chromogenic signal.

### 4.9. N-Glycoprotein Spatial Analysis

The evaluation of myeloperoxidase expression in FFPE tissues by immunohistochemistry was performed similarly to that described for the GNL ligands. Briefly, FFPE tissue sections of 3 mm were deparaffinized and rehydrated by immersion in xylene (2 × 7 min), 100% ethanol (2 × 5 min), 96% ethanol (2 × 5 min), 70% ethanol (5 min), and water (5 min). Then, the tissues were subjected to heat-induced antigen retrieval by incubation in 1 mM ethylenediaminetetraacetic acid, pH 9, for 20 min. To block endogenous peroxidases, tissue sections were then exposed to 3–4% hydrogen peroxide for 5 min (Leica, Leica Microsystem). After incubation with 5% BSA in PBS for 1 h at RT, myeloperoxidase was detected using 1:500 anti-myeloperoxidase (1 h at RT; ab208670; Abcam, Cambridge, UK). This was followed by incubation, 30 min at RT, with the Vectastain ABC kit (Vector Laboratories, Newark, CA, USA) and 3, 3′-diaminobenzidine for 5 min. Additionally, the nuclei were stained with hematoxylin. A semi-quantitative analysis of slides was performed, evaluating the level of positive staining (low vs. high) for myeloperoxidase.

### 4.10. Mannosidases and Myeloperoxidase Transcript Analysis

*MANEA*, *MAN1A1*, *MAN1A2, MAN1B1*, and *MAN1C1*, as well as *MPO,* were examined using gene expression data from 407 TCGA bladder carcinoma (TCGA-BLCA) tumor tissues and 19 matched normal adjacent tissues retrieved from the UCSC Xena platform (http://xena.ucsc.edu/ (accessed on 1 March 2025)). In addition to gene expression profiles, associated clinicopathological features, including patient tumor stage and histological subtype, overall survival (OS), and progression-free survival (PFS) data, were also obtained.

Statistical comparisons between sample tissue types and tumor stages were conducted using the nonparametric Wilcoxon test. Correlation analysis was performed using Spearman’s correlation and visualized by correlograms using the “corrplot” R package. Survival analyses, including Kaplan–Meier estimations and log-rank tests, were conducted to evaluate differences in OS and PFS using the “survival” and “survminer” R packages. The optimal expression cut-off value for each gene was determined using the surv_cutpoint function for both the OS and PFS outcomes. A *p*-value < 0.05 was considered statistically significant. All statistical analyses were performed using R software (version 4.4.2).

### 4.11. Experimental Design and Statistical Rationale

Investigation of the glycome profile was performed on 5 healthy bladder tissues, 5 cystitis, 5 Ta low-grade tumors, 5 T1 high-grade tumors, and 8 muscle-invasive tumors (T2-T4). *N*-glycoprotein biomarker discovery was performed on the same Ta (*n* = 4) and T1 (*n* = 4) tumors, and on 4 muscle-invasive bladder tumors. All samples were processed and analyzed individually as biological replicates. Each tumor sample corresponds to a different patient. Spatial analysis by immunohistochemistry was performed in 29 cases. Considering the number of biological replicates, no technical replicates were performed. Tumor staging was assigned using clinical and pathological data. Parametric and non-parametric statistical tests, including one-way ANOVA with or without Welch correction and the Kruskal–Wallis test, were employed to compare the glycan expression levels across groups (healthy, cystitis, Ta, T1, and MIBC). Prior to analysis, outliers were identified and eliminated using the ROUT method, and data normality was assessed using the Shapiro–Wilk test. Regarding the immunohistochemistry results, a one-way ANOVA with Kruskal-Wallis’ test and a chi-square test were used to evaluate the statistical relationships between GNL or myeloperoxidase expression and disease stage, respectively. The clinical value was assessed using receiver operating characteristic (ROC) curves and maximizing the Youden Index (sensitivity + specificity—1) for the optimal threshold cutoff value. The relative abundances of oligomannose, neutral complex, and sialylated complex types, were used, considering two groups (non-cancerous—healthy and cystitis; cancerous tissues—Ta, T1, MIBC tumors) and biological replicates as described above. The statistical analysis was conducted using GraphPad Prism 9.5 (GraphPad Software, Boston, MA, USA).

## Data Availability

The data generated in this study are included in the main article or the Supplementary Materials. The mass spectrometry glycoproteomics data have been deposited in the ProteomeXchange Consortium via the PRIDE [65] partner repository with the dataset identifier PXD063671. The mass spectrometry glycomics data have been deposited in the GlycoPOST repository [66] with the ID GPST000585.

## Supplementary Materials

The following supporting information can be downloaded at https://www.mdpi.com/article/10.3390/ijms26104891/s1.

## References

[1] Bray F., Laversanne M., Sung H., Ferlay J., Siegel R.L., Soerjomataram I., Jemal A. (2024). Global cancer statistics 2022: GLOBOCAN estimates of incidence and mortality worldwide for 36 cancers in 185 countries. CA Cancer J. Clin..

[2] Scilipoti P., Moschini M., Li R., Lerner S.P., Black P.C., Necchi A., Roupret M., Shariat S.F., Gupta S., Morgans A.K. (2024). The Financial Burden of Localized and Metastatic Bladder Cancer. Eur. Urol..

[3] Babjuk M., Burger M., Capoun O., Cohen D., Comperat E.M., Dominguez Escrig J.L., Gontero P., Liedberg F., Masson-Lecomte A., Mostafid A.H. (2022). European Association of Urology Guidelines on Non-muscle-invasive Bladder Cancer (Ta, T1, and Carcinoma in Situ). Eur. Urol..

[4] Alfred Witjes J., Max Bruins H., Carrion A., Cathomas R., Comperat E., Efstathiou J.A., Fietkau R., Gakis G., Lorch A., Martini A. (2024). European Association of Urology Guidelines on Muscle-invasive and Metastatic Bladder Cancer: Summary of the 2023 Guidelines. Eur. Urol..

[5] Ferreira J.A., Relvas-Santos M., Peixoto A., Silva A.M.N., Lara Santos L. (2021). Glycoproteogenomics: Setting the Course for Next-generation Cancer Neoantigen Discovery for Cancer Vaccines. Genom. Proteom. Bioinform..

[6] Peixoto A., Relvas-Santos M., Azevedo R., Santos L.L., Ferreira J.A. (2019). Protein Glycosylation and Tumor Microenvironment Alterations Driving Cancer Hallmarks. Front. Oncol..

[7] RodrIguez E., Schetters S.T.T., van Kooyk Y. (2018). The tumour glyco-code as a novel immune checkpoint for immunotherapy. Nat. Rev. Immunol..

[8] Stanczak M.A., Rodrigues Mantuano N., Kirchhammer N., Sanin D.E., Jacob F., Coelho R., Everest-Dass A.V., Wang J., Trefny M.P., Monaco G. (2022). Targeting cancer glycosylation repolarizes tumor-associated macrophages allowing effective immune checkpoint blockade. Sci. Transl. Med..

[9] Diniz F., Coelho P., Duarte H.O., Sarmento B., Reis C.A., Gomes J. (2022). Glycans as Targets for Drug Delivery in Cancer. Cancers.

[10] Chatterjee S., Ugonotti J., Lee L.Y., Everest-Dass A., Kawahara R., Thaysen-Andersen M. (2021). Trends in oligomannosylation and alpha1,2-mannosidase expression in human cancers. Oncotarget.

[11] Pinho S.S., Reis C.A. (2015). Glycosylation in cancer: Mechanisms and clinical implications. Nat. Rev. Cancer.

[12] Pucci M., Malagolini N., Dall’Olio F. (2021). Glycobiology of the Epithelial to Mesenchymal Transition. Biomedicines.

[13] Ferreira J.A., Videira P.A., Lima L., Pereira S., Silva M., Carrascal M., Severino P.F., Fernandes E., Almeida A., Costa C. (2013). Overexpression of tumour-associated carbohydrate antigen sialyl-Tn in advanced bladder tumours. Mol. Oncol..

[14] Lima L., Neves M., Oliveira M.I., Dieguez L., Freitas R., Azevedo R., Gaiteiro C., Soares J., Ferreira D., Peixoto A. (2017). Sialyl-Tn identifies muscle-invasive bladder cancer basal and luminal subtypes facing decreased survival, being expressed by circulating tumor cells and metastases. Urol. Oncol..

[15] Gaiteiro C., Soares J., Relvas-Santos M., Peixoto A., Ferreira D., Paulo P., Brandao A., Fernandes E., Azevedo R., Palmeira C. (2022). Glycoproteogenomics characterizes the CD44 splicing code associated with bladder cancer invasion. Theranostics.

[16] Carrascal M.A., Severino P.F., Guadalupe Cabral M., Silva M., Ferreira J.A., Calais F., Quinto H., Pen C., Ligeiro D., Santos L.L. (2014). Sialyl Tn-expressing bladder cancer cells induce a tolerogenic phenotype in innate and adaptive immune cells. Mol. Oncol..

[17] Loureiro L.R., Sousa D.P., Ferreira D., Chai W., Lima L., Pereira C., Lopes C.B., Correia V.G., Silva L.M., Li C. (2018). Novel monoclonal antibody L2A5 specifically targeting sialyl-Tn and short glycans terminated by alpha-2-6 sialic acids. Sci. Rep..

[18] Freitas R., Miranda A., Ferreira D., Relvas-Santos M., Castro F., Ferreira E., Gaiteiro C., Soares J., Cotton S., Goncalves M. (2024). A multivalent CD44 glycoconjugate vaccine candidate for cancer immunotherapy. J. Control. Release.

[19] Cotton S., Azevedo R., Gaiteiro C., Ferreira D., Lima L., Peixoto A., Fernandes E., Neves M., Neves D., Amaro T. (2017). Targeted O-glycoproteomics explored increased sialylation and identified MUC16 as a poor prognosis biomarker in advanced-stage bladder tumours. Mol. Oncol..

[20] Zhou S., Dong X., Veillon L., Huang Y., Mechref Y. (2017). LC-MS/MS analysis of permethylated N-glycans facilitating isomeric characterization. Anal. Bioanal. Chem..

[21] Bojar D., Meche L., Meng G., Eng W., Smith D.F., Cummings R.D., Mahal L.K. (2022). A Useful Guide to Lectin Binding: Machine-Learning Directed Annotation of 57 Unique Lectin Specificities. ACS Chem. Biol..

[22] Almeida P., Alves I., Fernandes A., Lima C., Freitas R., Braga I., Correia J., Jeronimo C., Pinho S.S. (2025). Mannose glycans as key players in trained immunity: A novel anti-tumoral catalyst. Biochim. Biophys. Acta Gen. Subj..

[23] Narimatsu Y., Joshi H.J., Nason R., Van Coillie J., Karlsson R., Sun L., Ye Z., Chen Y.H., Schjoldager K.T., Steentoft C. (2019). An Atlas of Human Glycosylation Pathways Enables Display of the Human Glycome by Gene Engineered Cells. Mol. Cell.

[24] Huang Y., Hong W., Wei X. (2022). The molecular mechanisms and therapeutic strategies of EMT in tumor progression and metastasis. J. Hematol. Oncol..

[25] Tan S.F., Dunton W., Liu X., Fox T.E., Morad S.A.F., Desai D., Doi K., Conaway M.R., Amin S., Claxton D.F. (2019). Acid ceramidase promotes drug resistance in acute myeloid leukemia through NF-kappaB-dependent P-glycoprotein upregulation. J. Lipid Res..

[26] Lai M., Realini N., La Ferla M., Passalacqua I., Matteoli G., Ganesan A., Pistello M., Mazzanti C.M., Piomelli D. (2017). Complete Acid Ceramidase ablation prevents cancer-initiating cell formation in melanoma cells. Sci. Rep..

[27] Saad A.F., Meacham W.D., Bai A., Anelli V., Elojeimy S., Mahdy A.E., Turner L.S., Cheng J., Bielawska A., Bielawski J. (2007). The functional effects of acid ceramidase overexpression in prostate cancer progression and resistance to chemotherapy. Cancer Biol. Ther..

[28] Blumenthal R.D., Hansen H.J., Goldenberg D.M. (2005). Inhibition of adhesion, invasion, and metastasis by antibodies targeting CEACAM6 (NCA-90) and CEACAM5 (Carcinoembryonic Antigen). Cancer Res..

[29] Tjondro H.C., Ugonotti J., Kawahara R., Chatterjee S., Loke I., Chen S., Soltermann F., Hinneburg H., Parker B.L., Venkatakrishnan V. (2021). Hyper-truncated Asn355- and Asn391-glycans modulate the activity of neutrophil granule myeloperoxidase. J. Biol. Chem..

[30] Feinberg H., Castelli R., Drickamer K., Seeberger P.H., Weis W.I. (2007). Multiple modes of binding enhance the affinity of DC-SIGN for high mannose N-linked glycans found on viral glycoproteins. J. Biol. Chem..

[31] Stavenhagen K., Laan L.C., Gao C., Mehta A.Y., Heimburg-Molinaro J., Glickman J.N., van Die I., Cummings R.D. (2021). Tumor cells express pauci- and oligomannosidic N-glycans in glycoproteins recognized by the mannose receptor (CD206). Cell Mol. Life Sci..

[32] Kawahara R., Ugonotti J., Chatterjee S., Tjondro H.C., Loke I., Parker B.L., Venkatakrishnan V., Dieckmann R., Sumer-Bayraktar Z., Karlsson-Bengtsson A. (2023). Glycoproteome remodeling and organelle-specific N-glycosylation accompany neutrophil granulopoiesis. Proc. Natl. Acad. Sci. USA.

[33] Sobala L.F., Fernandes P.Z., Hakki Z., Thompson A.J., Howe J.D., Hill M., Zitzmann N., Davies S., Stamataki Z., Butters T.D. (2020). Structure of human endo-alpha-1,2-mannosidase (MANEA), an antiviral host-glycosylation target. Proc. Natl. Acad. Sci. USA.

[34] Stanley P., Moremen K.W., Lewis N.E., Taniguchi N., Aebi M., Varki A., Cummings R.D., Esko J.D., Stanley P., Hart G.W., Aebi M., Mohnen D., Kinoshita T., Packer N.H., Prestegard J.H. (2022). N-Glycans. Essentials of Glycobiology.

[35] Sheinfeld J., Reuter V.E., Fair W.R., Cordon-Cardo C. (1992). Expression of blood group antigens in bladder cancer: Current concepts. Semin. Surg. Oncol..

[36] Peixoto A., Ferreira D., Miranda A., Relvas-Santos M., Freitas R., Veth T.S., Brandao A., Ferreira E., Paulo P., Cardoso M. (2025). Multilevel plasticity and altered glycosylation drive aggressiveness in hypoxic and glucose-deprived bladder cancer cells. iScience.

[37] Li Y., Fu B., Wang M., Chen W., Fan J., Li Y., Liu X., Wang J., Zhang Z., Lu H. (2025). Urinary extracellular vesicle N-glycomics identifies diagnostic glycosignatures for bladder cancer. Nat. Commun..

[38] Wallace E.N., West C.A., McDowell C.T., Lu X., Bruner E., Mehta A.S., Aoki-Kinoshita K.F., Angel P.M., Drake R.R. (2024). An N-glycome tissue atlas of 15 human normal and cancer tissue types determined by MALDI-imaging mass spectrometry. Sci. Rep..

[39] Leibovici D., Grossman H.B., Dinney C.P., Millikan R.E., Lerner S., Wang Y., Gu J., Dong Q., Wu X. (2005). Polymorphisms in inflammation genes and bladder cancer: From initiation to recurrence, progression, and survival. J. Clin. Oncol..

[40] Kissel T., Toes R.E.M., Huizinga T.W.J., Wuhrer M. (2023). Glycobiology of rheumatic diseases. Nat. Rev. Rheumatol..

[41] Gaifem J., Rodrigues C.S., Petralia F., Alves I., Leite-Gomes E., Cavadas B., Dias A.M., Moreira-Barbosa C., Reves J., Laird R.M. (2024). A unique serum IgG glycosylation signature predicts development of Crohn’s disease and is associated with pathogenic antibodies to mannose glycan. Nat. Immunol..

[42] Irvine E.B., Alter G. (2020). Understanding the role of antibody glycosylation through the lens of severe viral and bacterial diseases. Glycobiology.

[43] Peracaula R., Sarrats A., Rudd P.M. (2010). Liver proteins as sensor of human malignancies and inflammation. Proteom. Clin. Appl..

[44] Ohmi Y., Ise W., Harazono A., Takakura D., Fukuyama H., Baba Y., Narazaki M., Shoda H., Takahashi N., Ohkawa Y. (2016). Sialylation converts arthritogenic IgG into inhibitors of collagen-induced arthritis. Nat. Commun..

[45] Comperat E., Varinot J., Moroch J., Eymerit-Morin C., Brimo F. (2018). A practical guide to bladder cancer pathology. Nat. Rev. Urol..

[46] Yang G., Tan Z., Lu W., Guo J., Yu H., Yu J., Sun C., Qi X., Li Z., Guan F. (2015). Quantitative glycome analysis of N-glycan patterns in bladder cancer vs normal bladder cells using an integrated strategy. J. Proteome Res..

[47] Liu X., Nie H., Zhang Y., Yao Y., Maitikabili A., Qu Y., Shi S., Chen C., Li Y. (2013). Cell surface-specific N-glycan profiling in breast cancer. PLoS ONE.

[48] Sethi M.K., Thaysen-Andersen M., Smith J.T., Baker M.S., Packer N.H., Hancock W.S., Fanayan S. (2014). Comparative N-glycan profiling of colorectal cancer cell lines reveals unique bisecting GlcNAc and alpha-2,3-linked sialic acid determinants are associated with membrane proteins of the more metastatic/aggressive cell lines. J. Proteome Res..

[49] Moginger U., Grunewald S., Hennig R., Kuo C.W., Schirmeister F., Voth H., Rapp E., Khoo K.H., Seeberger P.H., Simon J.C. (2018). Alterations of the Human Skin N- and O-Glycome in Basal Cell Carcinoma and Squamous Cell Carcinoma. Front. Oncol..

[50] Boyaval F., van Zeijl R., Dalebout H., Holst S., van Pelt G., Farina-Sarasqueta A., Mesker W., Tollenaar R., Morreau H., Wuhrer M. (2021). N-Glycomic Signature of Stage II Colorectal Cancer and Its Association With the Tumor Microenvironment. Mol. Cell Proteom..

[51] Ruhaak L.R., Taylor S.L., Stroble C., Nguyen U.T., Parker E.A., Song T., Lebrilla C.B., Rom W.N., Pass H., Kim K. (2015). Differential N-Glycosylation Patterns in Lung Adenocarcinoma Tissue. J. Proteome Res..

[52] Ozcan S., Barkauskas D.A., Renee Ruhaak L., Torres J., Cooke C.L., An H.J., Hua S., Williams C.C., Dimapasoc L.M., Han Kim J. (2014). Serum glycan signatures of gastric cancer. Cancer Prev. Res..

[53] West C.A., Wang M., Herrera H., Liang H., Black A., Angel P.M., Drake R.R., Mehta A.S. (2018). N-Linked Glycan Branching and Fucosylation Are Increased Directly in Hcc Tissue as Determined through in situ Glycan Imaging. J. Proteome Res..

[54] Drake R.R., McDowell C., West C., David F., Powers T.W., Nowling T., Bruner E., Mehta A.S., Angel P.M., Marlow L.A. (2020). Defining the human kidney N-glycome in normal and cancer tissues using MALDI imaging mass spectrometry. J. Mass Spectrom..

[55] McDowell C.T., Klamer Z., Hall J., West C.A., Wisniewski L., Powers T.W., Angel P.M., Mehta A.S., Lewin D.N., Haab B.B. (2021). Imaging Mass Spectrometry and Lectin Analysis of N-Linked Glycans in Carbohydrate Antigen-Defined Pancreatic Cancer Tissues. Mol. Cell Proteom..

[56] Broekhuis J.M., Lu D., Aryal R.P., Matsumoto Y., Pepi L.E., Chaves N., Gomez-Mayorga J.L., James B.C., Cummings R.D. (2024). Thyroid Carcinoma Glycoproteins Express Altered N-Glycans with 3-O-Sulfated Galactose Residues. Biomolecules.

[57] Gao C., Stavenhagen K., Eckmair B., McKitrick T.R., Mehta A.Y., Matsumoto Y., McQuillan A.M., Hanes M.S., Eris D., Baker K.J. (2021). Differential recognition of oligomannose isomers by glycan-binding proteins involved in innate and adaptive immunity. Sci. Adv..

[58] Cummings R.D. (2022). The mannose receptor ligands and the macrophage glycome. Curr. Opin. Struct. Biol..

[59] Park D.D., Phoomak C., Xu G., Olney L.P., Tran K.A., Park S.S., Haigh N.E., Luxardi G., Lert-Itthiporn W., Shimoda M. (2020). Metastasis of cholangiocarcinoma is promoted by extended high-mannose glycans. Proc. Natl. Acad. Sci. USA.

[60] Stevenson F.K., Forconi F. (2024). The essential microenvironmental role of oligomannoses specifically inserted into the antigen-binding sites of lymphoma cells. Blood.

[61] Boyaval F., Dalebout H., Van Zeijl R., Wang W., Farina-Sarasqueta A., Lageveen-Kammeijer G.S.M., Boonstra J.J., McDonnell L.A., Wuhrer M., Morreau H. (2022). High-Mannose N-Glycans as Malignant Progression Markers in Early-Stage Colorectal Cancer. Cancers.

[62] Tan Z., Lu W., Li X., Yang G., Guo J., Yu H., Li Z., Guan F. (2014). Altered N-Glycan expression profile in epithelial-to-mesenchymal transition of NMuMG cells revealed by an integrated strategy using mass spectrometry and glycogene and lectin microarray analysis. J. Proteome Res..

[63] Wang J., Li J.N., Cui Z., Zhao M.H. (2018). Deglycosylation influences the oxidation activity and antigenicity of myeloperoxidase. Nephrology.

[64] Ceroni A., Maass K., Geyer H., Geyer R., Dell A., Haslam S.M. (2008). GlycoWorkbench: A tool for the computer-assisted annotation of mass spectra of glycans. J. Proteome Res..

[65] Perez-Riverol Y., Bandla C., Kundu D.J., Kamatchinathan S., Bai J., Hewapathirana S., John N.S., Prakash A., Walzer M., Wang S. (2025). The PRIDE database at 20 years: 2025 update. Nucleic Acids Res..

[66] Watanabe Y., Aoki-Kinoshita K.F., Ishihama Y., Okuda S. (2021). GlycoPOST realizes FAIR principles for glycomics mass spectrometry data. Nucleic Acids Res..

