# Supplementary material for: Glycomics and Glycoproteomics Reveal Distinct Oligomannose Carriers Across Bladder Cancer Stages
Source: Int J Mol Sci. 2025 May 20;26(10):4891. doi: 10.3390/ijms26104891 (PMC12112682; doi:10.3390/ijms26104891)

Supplementary file S1

for

Glycomics and Glycoproteomics Reveal Distinct Oligomannose Carriers Across Bladder Cancer Stages

Marta Relvas-Santos[1,2,3], Dylan Ferreira[1,2,4], Andreia Brandão[5], Luis Pedro Afonso[1,6], Lúcio Lara Santos[1,4,7,8], André M N Silva[2,3,8], José Alexandre Ferreira[1,2,8]

[1]Experimental Pathology and Therapeutics Group, Research Center of IPO Porto (CI-IPOP)/CI-IPOP@RISE (Health Research Network), Portuguese Oncology Institute of Porto (IPO Porto)/Porto Comprehensive Cancer Center Raquel Seruca (Porto.CCC), R. Dr. António Bernardino de Almeida, 4200-072 Porto, Portugal; [2]ICBAS - School of Medicine and Biomedical Sciences, University of Porto, Rua Jorge Viterbo Ferreira 228, 4050-513 Porto, Portugal; [3]LAQV-REQUIMTE, Department of Chemistry and Biochemistry, Faculty of Sciences, University of Porto, Rua do Campo Alegre s/n 4169-007 Porto, Portugal; [4]School of Medicine and Biomedical Sciences, Fernando Pessoa University, Avenida Fernando Pessoa, 4420-096 Gondomar, Portugal; [5]Cancer Genetics Group, Research Center of IPO Porto (CI-IPOP)/CI-IPOP@RISE (Health Research Network), Portuguese Oncology Institute of Porto (IPO Porto)/Porto Comprehensive Cancer Center Raquel Seruca (Porto.CCC), R. Dr. António Bernardino de Almeida, 4200-072 Porto, Portugal; [6]Department of Pathology, Portuguese Oncology Institute of Porto (IPO Porto)/Porto Comprehensive Cancer Center Raquel Seruca (Porto.CCC), R. Dr. António Bernardino de Almeida, 4200-072 Porto, Portugal; [7]Department of Surgical Oncology, Portuguese Oncology Institute of Porto (IPO-Porto), R. Dr. António Bernardino de Almeida, 4200-072 Porto, Portugal; [8]GlycoMatters Biotech, 4500-162 Espinho, Portugal

Corresponding author

José Alexandre Ferreira

Experimental Pathology and Therapeutics Group, Research Centre, Portuguese Oncology Institute of Porto,

R. Dr. António Bernardino de Almeida 4200-072 Porto, Portugal; Tel. +351 225084000 (ext. 5111).

Email: jose.a.ferreira@ipoporto.min-saude.pt

Annotation key

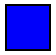 *N*-acetylglucosamine (GlcNAc)

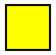 *N*-acetylgalactosamine (GalNAc)

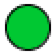 Mannose (Man)

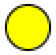 Galactose (Gal)

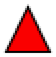 Fucose (Fuc)

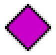 N-acetylneuraminic acid (NeuAc)

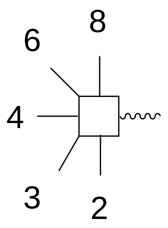

not defined

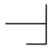 exclusion of the glycosidic linkage’s oxygen in the fragment ion

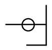 inclusion of the glycosidic linkage’s oxygen in the fragment ion

Abbreviations

#: scan number or sample number

RT: retention time

NL: normalized level

Ta: bladder tumor Ta stage low-grade

MI: muscle-invasive bladder tumour

Fragmentation of permethylated B ions

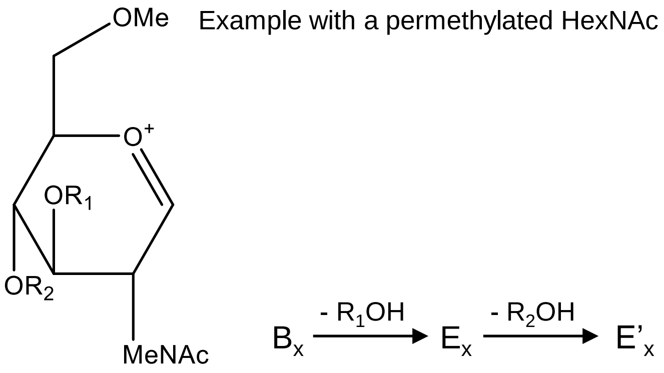

The supplementary file S2 contains:

- *N*-glycan base peak chromatograms for all samples analysed by mass spectrometry;
- Ions (*m/z* values) shown in each chromatogram;
- Library of identified glycan structures, presenting:
    - example of full ms showing the isotopic distribution of the identified ions associated to each glycan structure
    - example of HCD ms2 for each structure manually annotated;
- Library of glycoproteins identified in bladder tumour tissues, presenting:
    - Example of annotated glycoPSM for myeloperoxidase, considering all samples in which glycoproteins were identified and the different annotated glycan compositions
    - Example of annotated PSM myeloperoxidase, considering all samples in which glycoproteins were identified

***N*-glycan base peak chromatogram for healthy bladder samples**

The most abundant ions (z=1, z=2, or z=3) for each glycan structure are represented.

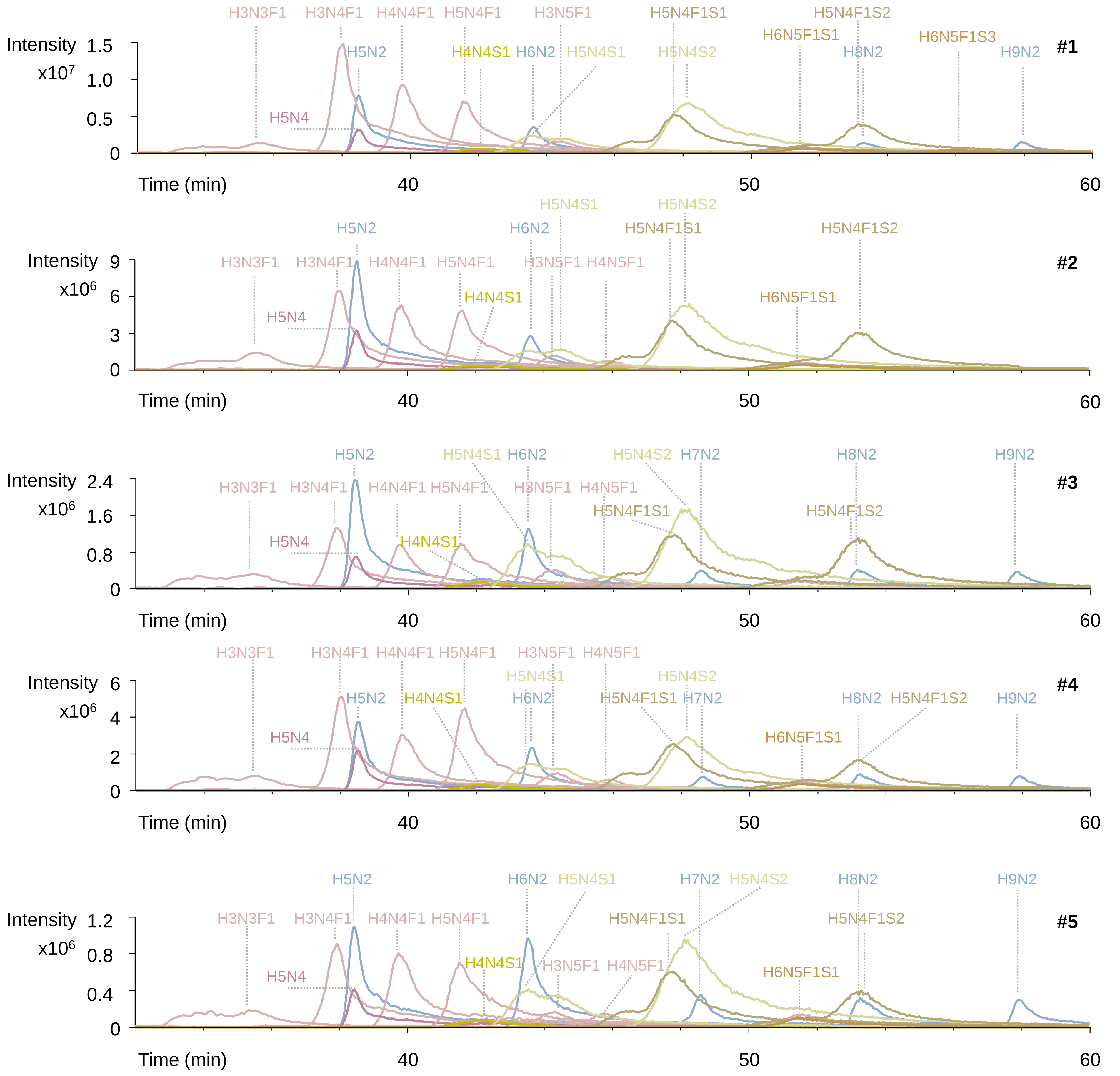

***N*-glycan base peak chromatogram for cystitis samples**

The most abundant ions (z=1, z=2, or z=3) for each glycan structure are represented.

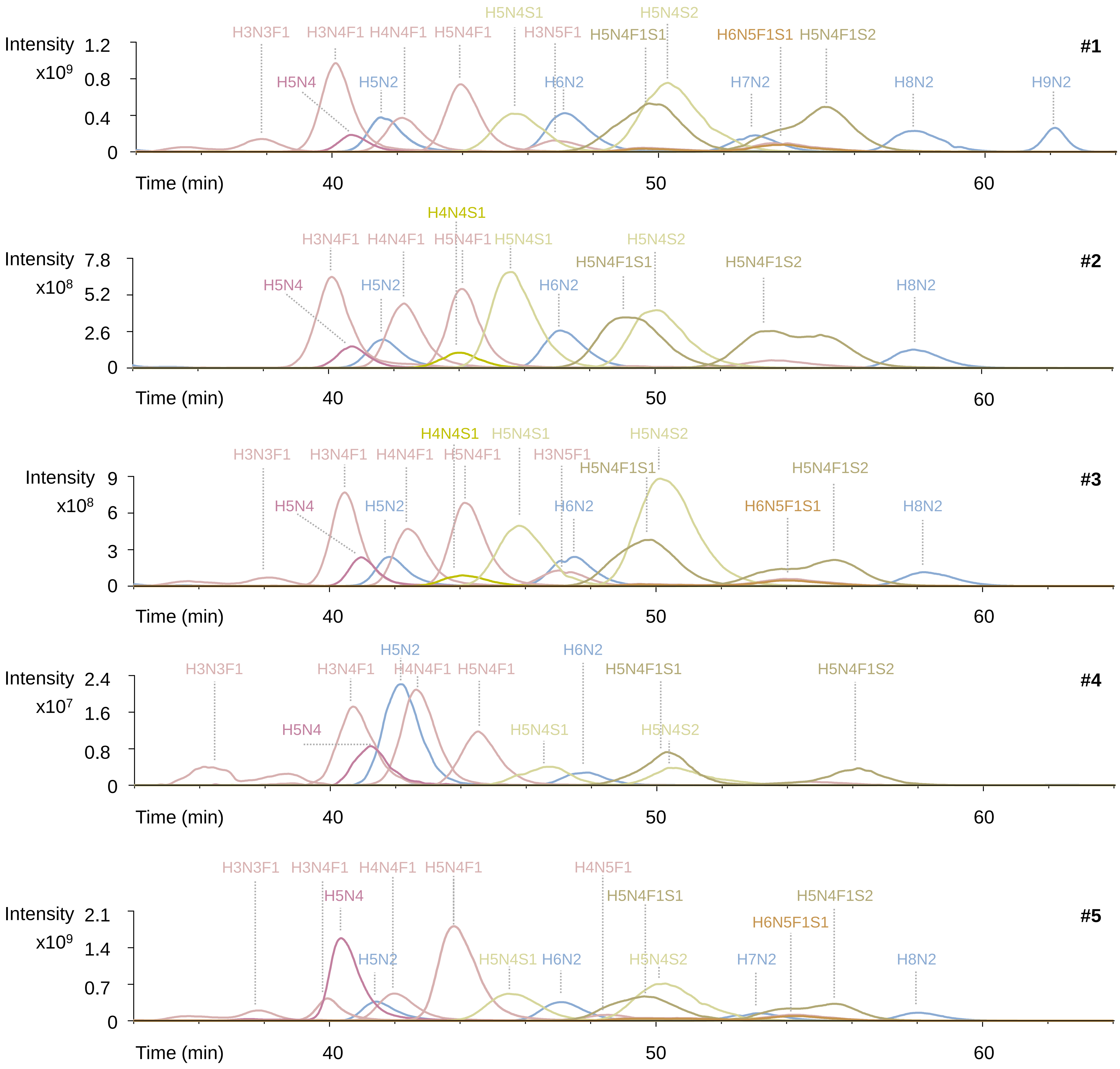

_N_-glycan base peak chromatogram for tumour Ta samples

The most abundant ions (z=1, z=2, or z=3) for each glycan structure are represented.

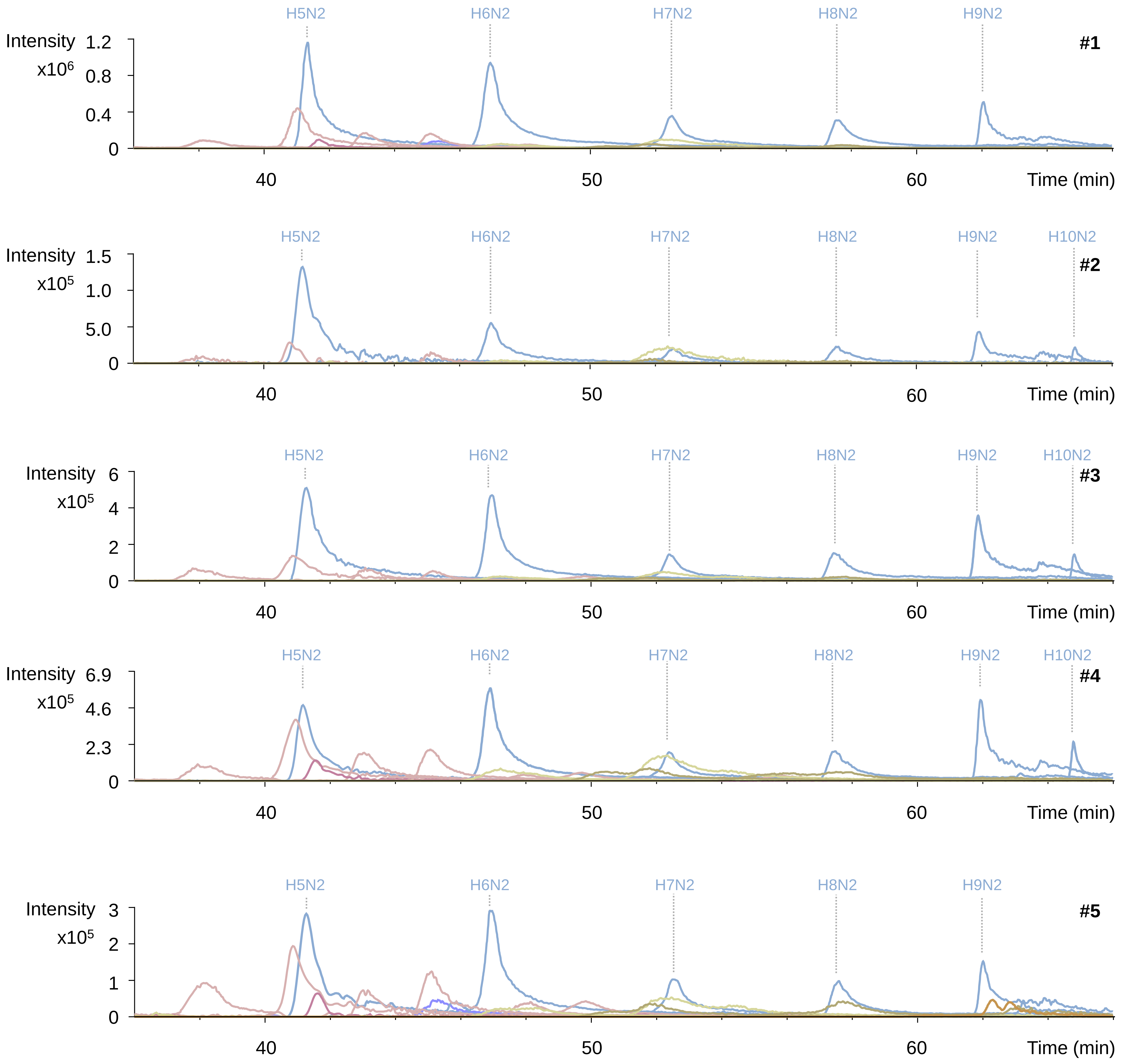

***N*-glycan base peak chromatogram for tumour Ta samples (zoom in low abundant species)**

The most abundant ions (z=1, z=2, or z=3) for each glycan structure are represented.

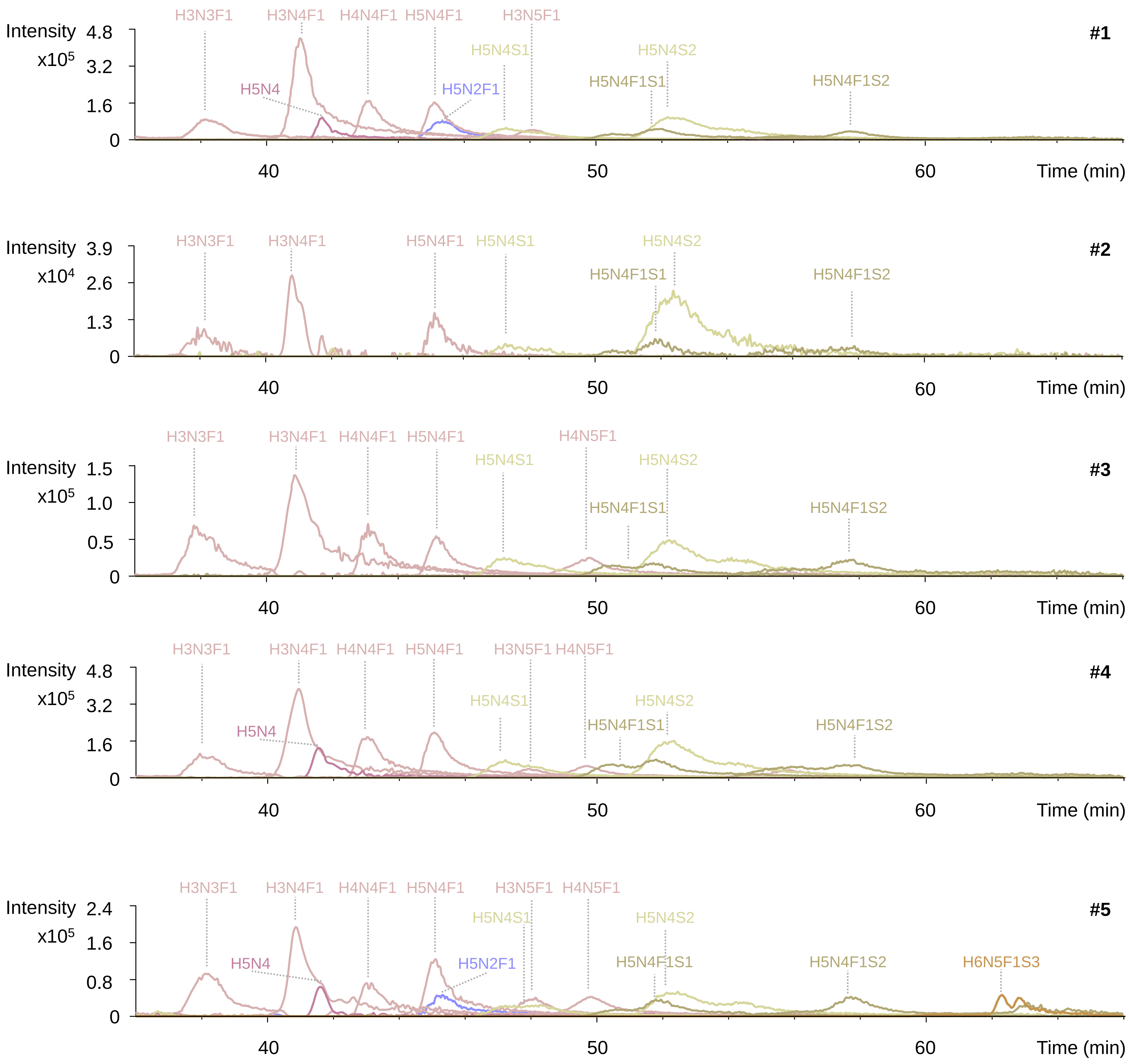

***N*-glycan base peak chromatogram for tumour T1 samples**

The most abundant ions (z=1, z=2, or z=3) for each glycan structure are represented.

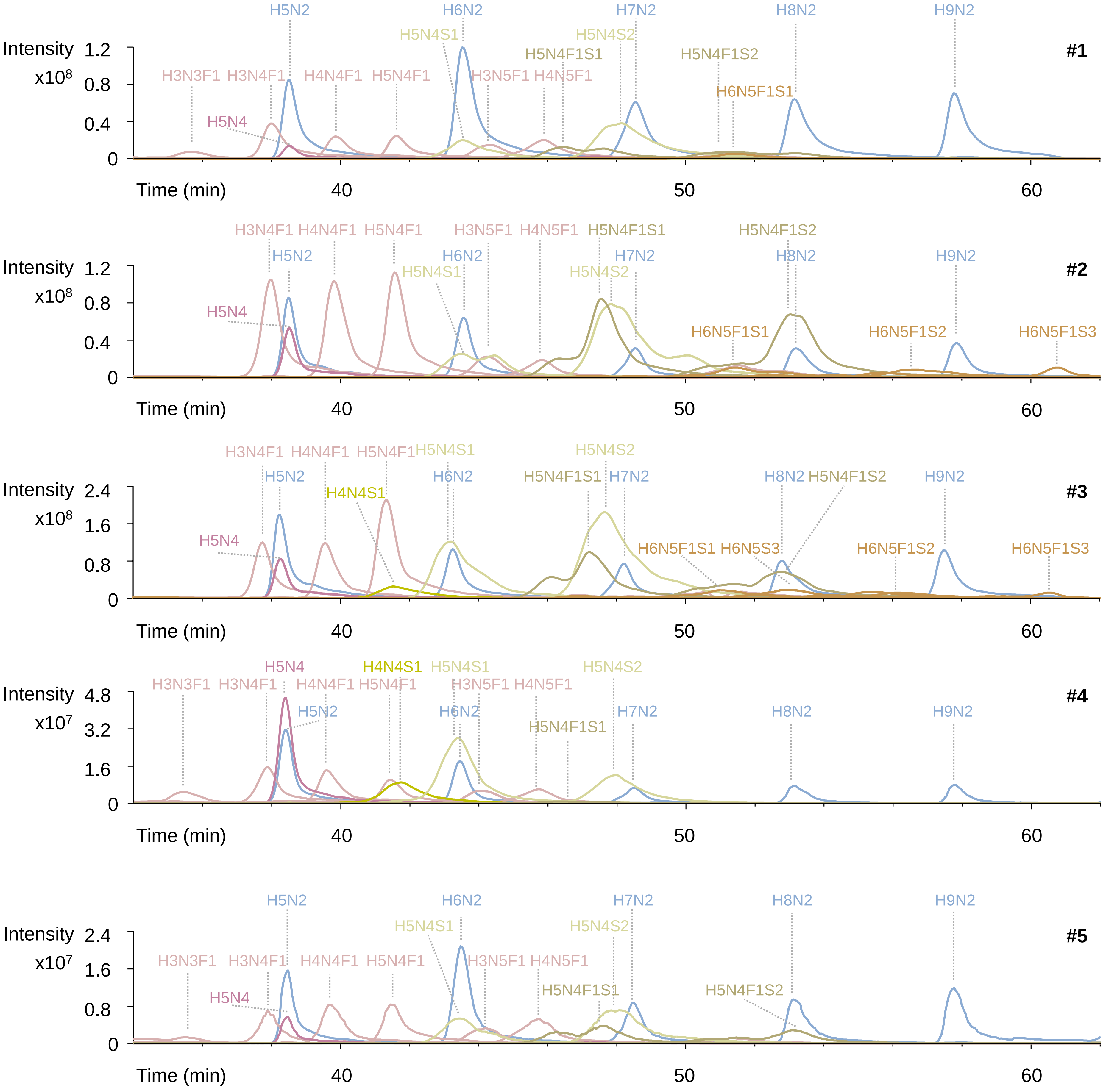

***N*-glycan base peak chromatogram for muscle-invasive tumour samples**

The most abundant ions (z=1, z=2, or z=3) for each glycan structure are represented.

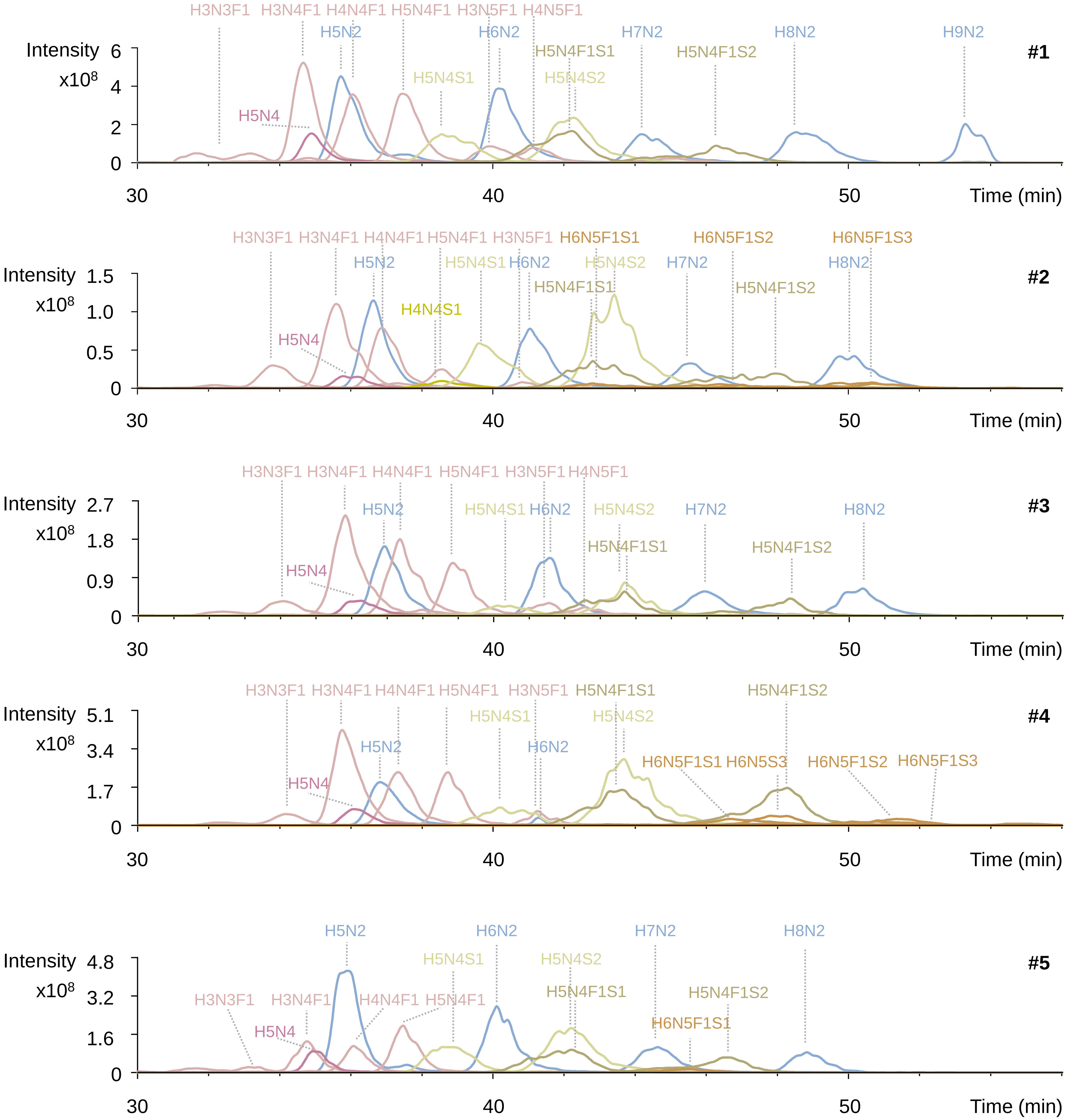

***N*-glycan base peak chromatogram for muscle-invasive tumour samples**

The most abundant ions (z=1, z=2, or z=3) for each glycan structure are represented.

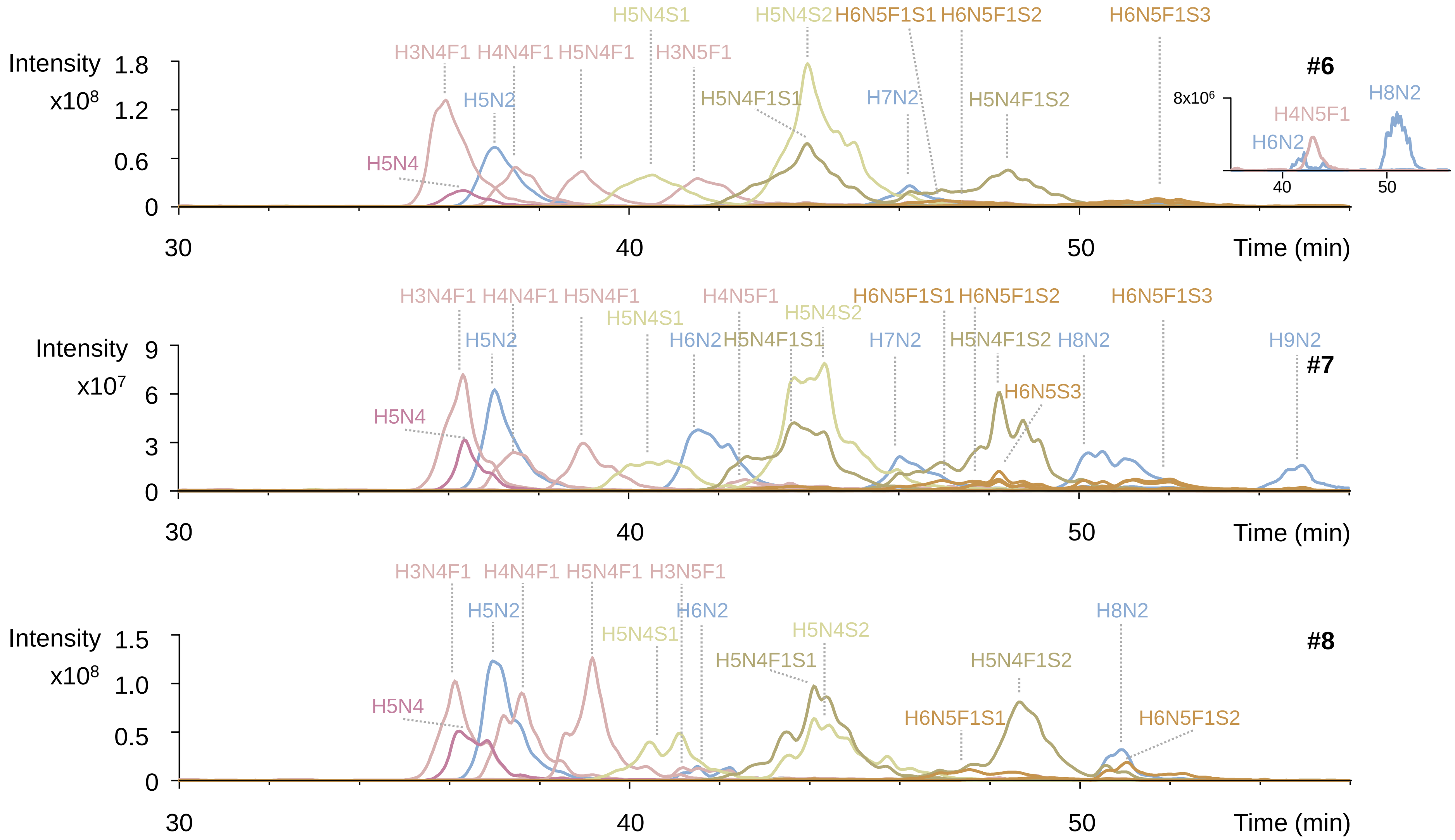

Ions (*m/z* values) shown in all chromatograms

Healthy bladder	#1	#2	#3	#4	#5
	787.4196 (z=2)	787.4196 (z=2)	787.4196 (z=2)	787.4196 (z=2)	787.4196 (z=2)
	889.4695 (z=2)	889.4695 (z=2)	889.4695 (z=2)	889.4695 (z=2)	889.4695 (z=2)
	1093.5693 (z=2)	792.9276 (z=2)	991.5194 (z=2)	991.5194 (z=2)	991.5194 (z=2)
	1195.6192 (z=2)	915.4908 (z=2)	1093.5693 (z=2)	1093.5693 (z=2)	1093.5693 (z=2)
	792.9276 (z=2)	1038.0539 (z=2)	1195.6192 (z=2)	1195.6192 (z=2)	1195.6192 (z=2)
	915.4908 (z=2)	1017.5407 (z=2)	792.9276 (z=2)	792.9276 (z=2)	792.9276 (z=2)
	1038.0539 (z=2)	1140.1038 (z=2)	915.4908 (z=2)	915.4908 (z=2)	915.4908 (z=2)
	1017.5407 (z=2)	1032.5459 (z=2)	1038.0539 (z=2)	1038.0539 (z=2)	1038.0539 (z=2)
	1032.5459 (z=2)	1119.5905 (z=2)	1017.5407 (z=2)	1017.5407 (z=2)	1017.5407 (z=2)
	1119.5905 (z=2)	1111.0829 (z=2)	1140.1038 (z=2)	1140.1038 (z=2)	1140.1038 (z=2)
	1111.0829 (z=2)	809.0909 (z=3)	1032.5459 (z=2)	1032.5459 (z=2)	1032.5459 (z=2)
	809.0909 (z=3)	929.4822 (z=3)	1119.5905 (z=2)	1119.5905 (z=2)	1119.5905 (z=2)
	929.4822 (z=3)	867.1207 (z=3)	1111.0829 (z=2)	1111.0829 (z=2)	1111.0829 (z=2)
	867.1207 (z=3)	987.5119 (z=3)	809.0909 (z=3)	809.0909 (z=3)	809.0909 (z=3)
	987.5119 (z=3)	1016.8627 (z=3)	929.4822 (z=3)	929.4822 (z=3)	929.4822 (z=3)
	1016.8627 (z=3)		867.1207 (z=3)	867.1207 (z=3)	867.1207 (z=3)
	1257.6452 (z=3)		987.5119 (z=3)	987.5119 (z=3)	987.5119 (z=3)
				1016.8627 (z=3)	1016.8627 (z=3)

Cystitis	#1	#2	#3	#4	#5
	787.4196 (z=2)	787.4196 (z=2)	787.4196 (z=2)	787.4196 (z=2)	787.4196 (z=2)
	889.4695 (z=2)	889.4695 (z=2)	889.4695 (z=2)	889.4695 (z=2)	889.4695 (z=2)
	991.5194 (z=2)	1093.5693 (z=2)	1093.5693 (z=2)	792.9276 (z=2)	991.5194 (z=2)
	1093.5693 (z=2)	915.4908 (z=2)	792.9276 (z=2)	915.4908 (z=2)	1093.5693 (z=2)
	1195.6192 (z=2)	1017.5407 (z=2)	915.4908 (z=2)	1017.5407 (z=2)	792.9276 (z=2)
	792.9276 (z=2)	1032.5459 (z=2)	1038.0539 (z=2)	1032.5459 (z=2)	915.4908 (z=2)
	915.4908 (z=2)	1119.5905 (z=2)	1017.5407 (z=2)	1119.5905 (z=2)	1017.5407 (z=2)
	1038.0539 (z=2)	1111.0829 (z=2)	1032.5459 (z=2)	809.0909 (z=3)	1140.1038 (z=2)
	1017.5407 (z=2)	1213.1328 (z=2)	1119.5905 (z=2)	929.4822 (z=3)	1032.5459 (z=2)
	1032.5459 (z=2)	1393.7196 (z=2)	1111.0829 (z=2)	867.1207 (z=3)	1119.5905 (z=2)
	1119.5905 (z=2)	867.1207 (z=3)	809.0909 (z=3)	987.5119 (z=3)	1213.1328 (z=2)
	1213.1328 (z=2)	987.5119 (z=3)	929.4822 (z=3)		929.4822 (z=3)
	929.4822 (z=3)		867.1207 (z=3)		867.1207 (z=3)
	867.1207 (z=3)		987.5119 (z=3)		987.5119 (z=3)
	987.5119 (z=3)		1016.8627 (z=3)		1016.8627 (z=3)
	1016.8627 (z=3)				

Ions (*m/z* values) shown in all chromatograms

Tumours Ta	#1	#2	#3	#4	#5
	787.4196 (z=2)	787.4196 (z=2)	787.4196 (z=2)	787.4196 (z=2)	787.4196 (z=2)
	874.4642 (z=2)	889.4695 (z=2)	889.4695 (z=2)	889.4695 (z=2)	874.4642 (z=2)
	889.4695 (z=2)	991.5194 (z=2)	991.5194 (z=2)	991.5194 (z=2)	889.4695 (z=2)
	991.5194 (z=2)	1093.5693 (z=2)	1093.5693 (z=2)	1093.5693 (z=2)	991.5194 (z=2)
	1093.5693 (z=2)	1195.6192 (z=2)	1195.6192 (z=2)	1195.6192 (z=2)	1093.5693 (z=2)
	1195.6192 (z=2)	1297.6690 (z=2)	1297.6690 (z=2)	1297.6690 (z=2)	1195.6192 (z=2)
	792.9276 (z=2)	792.9276 (z=2)	792.9276 (z=2)	792.9276 (z=2)	792.9276 (z=2)
	915.4908 (z=2)	915.4908 (z=2)	915.4908 (z=2)	915.4908 (z=2)	915.4908 (z=2)
	1038.0539 (z=2)	1119.5905 (z=2)	1017.5407 (z=2)	1038.0539 (z=2)	1038.0539 (z=2)
	1017.5407 (z=2)	809.0909 (z=3)	1140.1038 (z=2)	1017.5407 (z=2)	1017.5407 (z=2)
	1032.5459 (z=2)	929.4822 (z=3)	1119.5905 (z=2)	1140.1038 (z=2)	1140.1038 (z=2)
	1119.5905 (z=2)	867.1207 (z=3)	809.0909 (z=3)	1032.5459 (z=2)	1032.5459 (z=2)
	809.0909 (z=3)	987.5119 (z=3)	929.4822 (z=3)	1119.5905 (z=2)	1119.5905 (z=2)
	929.4822 (z=3)		867.1207 (z=3)	809.0909 (z=3)	809.0909 (z=3)
	867.1207 (z=3)		987.5119 (z=3)	929.4822 (z=3)	929.4822 (z=3)
	987.5119 (z=3)			867.1207 (z=3)	867.1207 (z=3)
				987.5119 (z=3)	987.5119 (z=3)
					1257.6452 (z=3)

Tumours T1	#1	#2	#3	#4	#5
	787.4196 (z=2)	787.4196 (z=2)	787.4196 (z=2)	787.4196 (z=2)	787.4196 (z=2)
	889.4695 (z=2)	889.4695 (z=2)	889.4695 (z=2)	889.4695 (z=2)	889.4695 (z=2)
	991.5194 (z=2)	991.5194 (z=2)	991.5194 (z=2)	991.5194 (z=2)	991.5194 (z=2)
	1093.5693 (z=2)	1093.5693 (z=2)	1093.5693 (z=2)	1093.5693 (z=2)	1093.5693 (z=2)
	1195.6192 (z=2)	1195.6192 (z=2)	1195.6192 (z=2)	1195.6192 (z=2)	1195.6192 (z=2)
	792.9276 (z=2)	915.4908 (z=2)	915.4908 (z=2)	792.9276 (z=2)	792.9276 (z=2)
	915.4908 (z=2)	1038.0539 (z=2)	1017.5407 (z=2)	915.4908 (z=2)	915.4908 (z=2)
	1038.0539 (z=2)	1017.5407 (z=2)	1032.5459 (z=2)	1038.0539 (z=2)	1038.0539 (z=2)
	1017.5407 (z=2)	1140.1038 (z=2)	1119.5905 (z=2)	1017.5407 (z=2)	1017.5407 (z=2)
	1140.1038 (z=2)	1032.5459 (z=2)	1111.0829 (z=2)	1140.1038 (z=2)	1140.1038 (z=2)
	1032.5459 (z=2)	1119.5905 (z=2)	1213.1328 (z=2)	1032.5459 (z=2)	1032.5459 (z=2)
	1119.5905 (z=2)	1213.1328 (z=2)	929.4822 (z=3)	1119.5905 (z=2)	1119.5905 (z=2)
	1213.1328 (z=2)	929.4822 (z=3)	1300.1774 (z=2)	1111.0829 (z=2)	1213.1328 (z=2)
	929.4822 (z=3)	867.1207 (z=3)	987.5119 (z=3)	1213.1328 (z=2)	929.4822 (z=3)
	867.1207 (z=3)	987.5119 (z=3)	1199.6154 (z=3)	929.4822 (z=3)	867.1207 (z=3)
	987.5119 (z=3)	1016.8627 (z=3)	1016.8627 (z=3)	1300.1774 (z=2)	987.5119 (z=3)
	1016.8627 (z=3)	1137.2539 (z=3)	1137.2539 (z=3)		
		1257.6452 (z=3)	1257.6452 (z=3)		

Ions (*m/z* values) shown in all chromatograms

Muscle-invasive tumours	#1	#2	#3	#4	#5	#6	#7	#8
	1573.8320 (z=1)	787.4196 (z=2)	787.4196 (z=2)	787.4196 (z=2)	1573.8320 (z=1)	787.4196 (z=2)	787.4196 (z=2)	787.4196 (z=2)
	1777.9317 (z=1)	889.4695 (z=2)	889.4695 (z=2)	1777.9317 (z=1)	1777.9317 (z=1)	1777.9317 (z=1)	889.4695 (z=2)	1777.9317 (z=1)
	1982.0315 (z=1)	991.5194 (z=2)	991.5194 (z=2)	792.9276 (z=2)	1982.0315 (z=1)	991.5194 (z=2)	991.5194 (z=2)	2186.1313 (z=1)
	2186.1313 (z=1)	792.9276 (z=2)	1093.5693 (z=2)	915.4908 (z=2)	2186.1313 (z=1)	2186.1313 (z=1)	1093.5693 (z=2)	915.4908 (z=2)
	1195.6192 (z=2)	915.4908 (z=2)	792.9276 (z=2)	1038.0539 (z=2)	792.9276 (z=2)	915.4908 (z=2)	1195.6192 (z=2)	1038.0539 (z=2)
	792.9276 (z=2)	1038.0539 (z=2)	915.4908 (z=2)	1017.5407 (z=2)	915.4908 (z=2)	1038.0539 (z=2)	915.4908 (z=2)	1017.5407 (z=2)
	915.4908 (z=2)	1017.5407 (z=2)	1038.0539 (z=2)	1032.5459 (z=2)	1017.5407 (z=2)	1017.5407 (z=2)	1017.5407 (z=2)	1032.5459 (z=2)
	1038.0539 (z=2)	1032.5459 (z=2)	1017.5407 (z=2)	1119.5905 (z=2)	1032.5459 (z=2)	760.4050 (z=3)	1140.1038 (z=2)	1119.5905 (z=2)
	1017.5407 (z=2)	1119.5905 (z=2)	1140.1038 (z=2)	809.0909 (z=3)	1119.5905 (z=2)	1032.5459 (z=2)	1032.5459 (z=2)	809.0909 (z=3)
	1140.1038 (z=2)	741.0577 (z=3)	1032.5459 (z=2)	929.4822 (z=3)	1213.1328 (z=2)	1119.5905 (z=2)	1119.5905 (z=2)	929.4822 (z=3)
	1032.5459 (z=2)	809.0909 (z=3)	1119.5905 (z=2)	867.1207 (z=3)	929.4822 (z=3)	809.0909 (z=3)	809.0909 (z=3)	867.1207 (z=3)
	1119.5905 (z=2)	929.4822 (z=3)	809.0909 (z=3)	987.5119 (z=3)	1300.1774 (z=2)	929.4822 (z=3)	929.4822 (z=3)	987.5119 (z=3)
	1213.1328 (z=2)	867.1207 (z=3)	929.4822 (z=3)	1199.6154 (z=3)	1480.7642 (z=2)	867.1207 (z=3)	867.1207 (z=3)	1016.8627 (z=3)
	1393.7196 (z=2)	987.5119 (z=3)	867.1207 (z=3)	1016.8627 (z=3)	1016.8627 (z=3)	987.5119 (z=3)	987.5119 (z=3)	1137.2539 (z=3)
	1300.7196 (z=2)	1016.8627 (z=3)	987.5119 (z=3)	1137.2539 (z=3)		1016.8627 (z=3)	1199.6154 (z=3)	
	1480.7642 (z=2)	1137.2539 (z=3)		1257.6452 (z=3)		1137.2539 (z=3)	1016.8627 (z=3)	
		1257.6452 (z=3)				1257.6452 (z=3)	1137.2539 (z=3)	
							1257.6452 (z=3)	

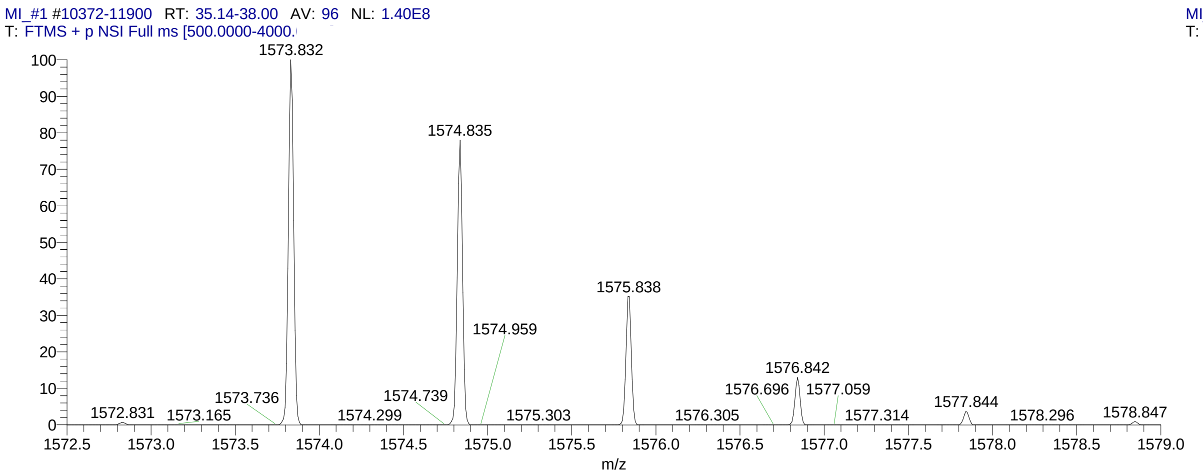

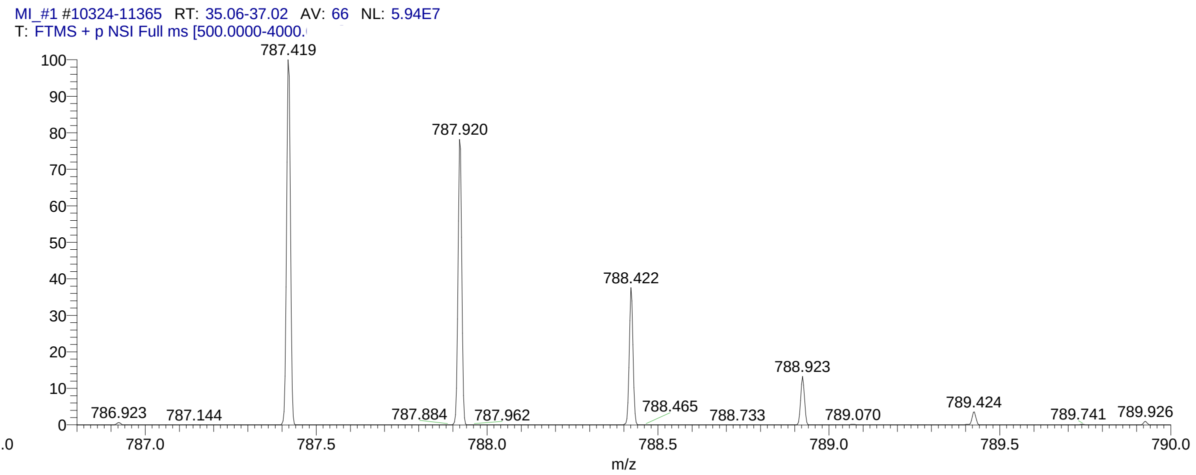

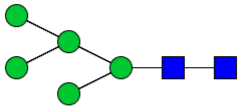

Glycan #1

HexNAc(2)Hex(5)

Permethylated, reduced

Theoretical *m/z* 1573.8320 (z=1)

Theoretical *m/z* 787.4196 (z=2)

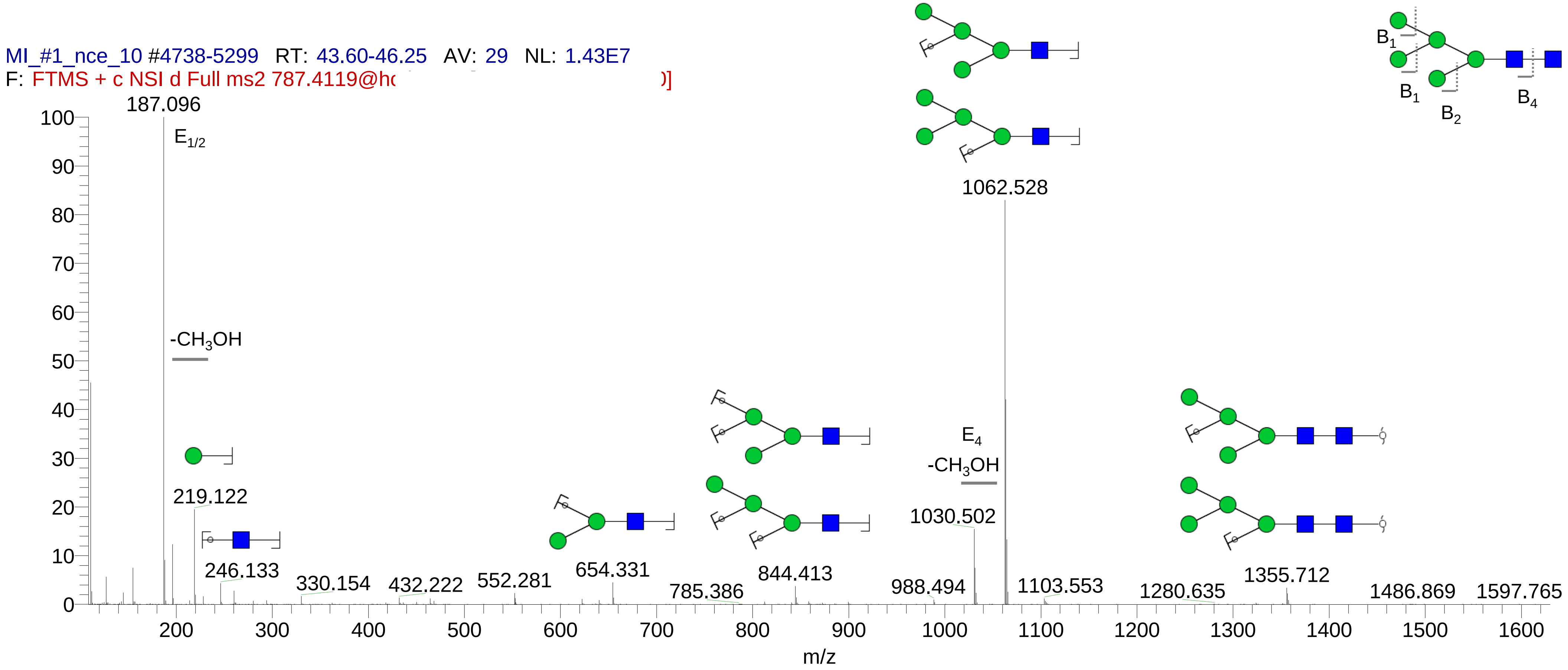

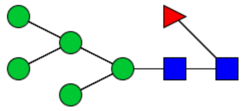

Glycan #2

HexNAc(2)Hex(5)Fuc(1)

Permethylated, reduced

Theoretical *m/z* 874.4642 (z=2)

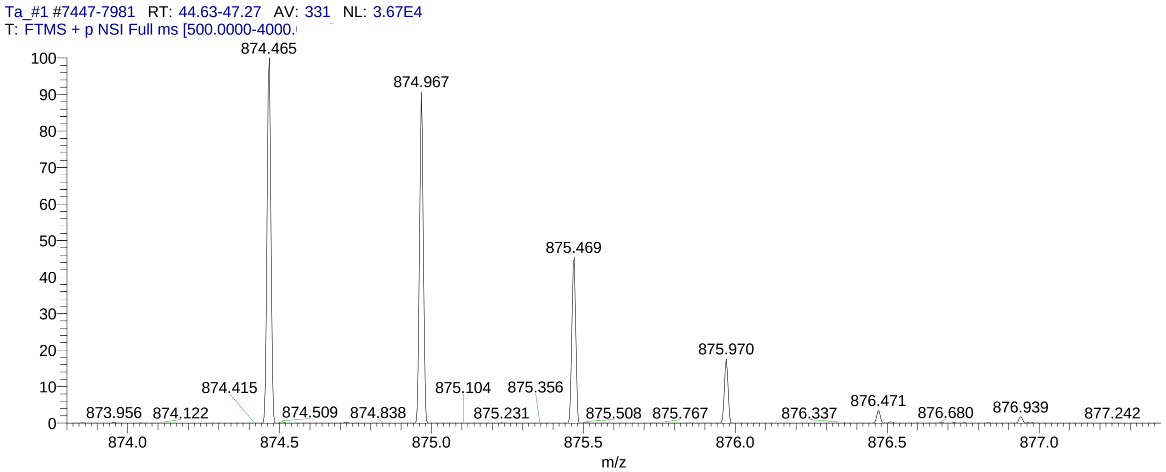

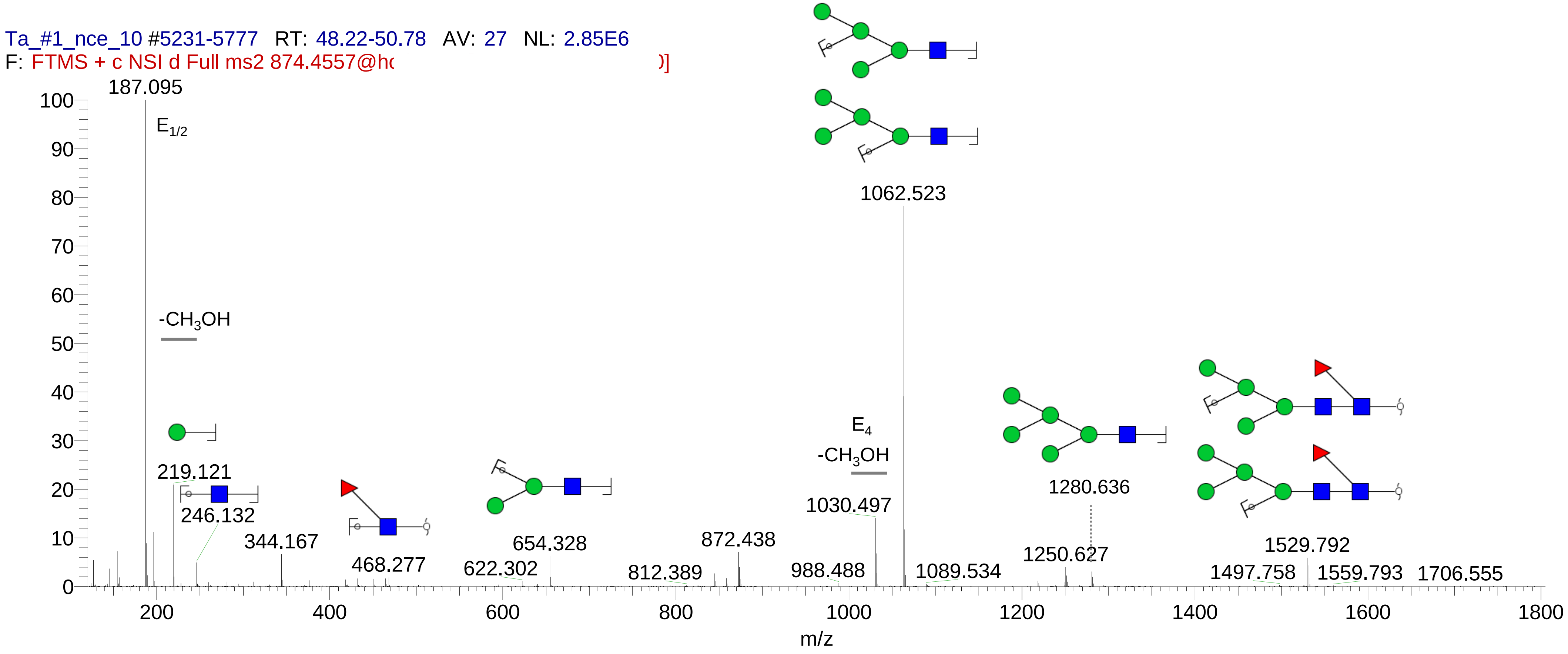

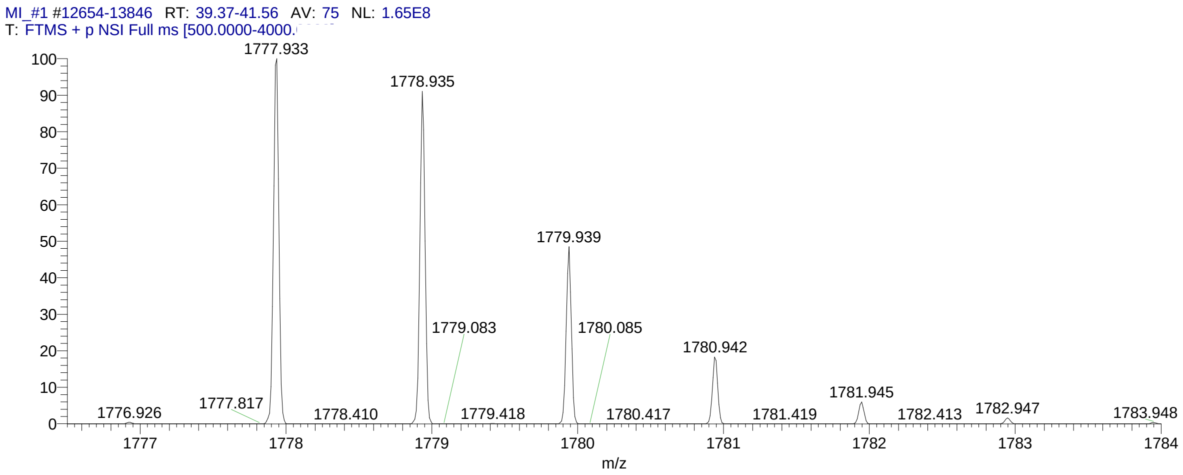

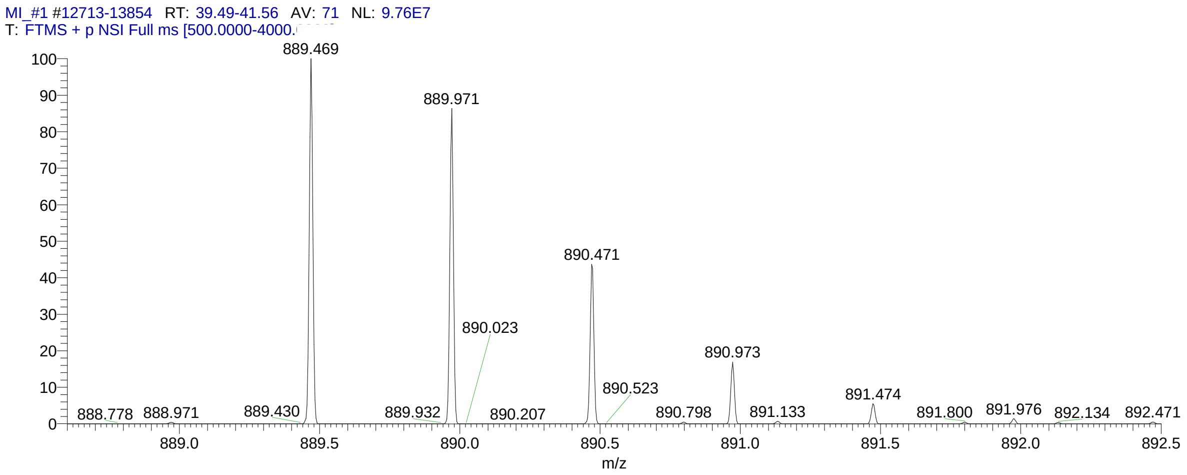

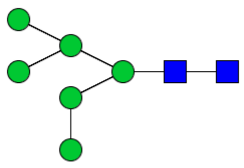

Glycan #3

HexNAc(2)Hex(6)

Permethylated, reduced

Theoretical *m/z* 1777.9317 (z=1)

Theoretical *m/z* 889.4695 (z=2)

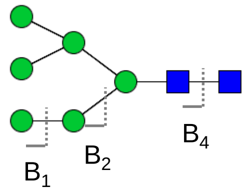

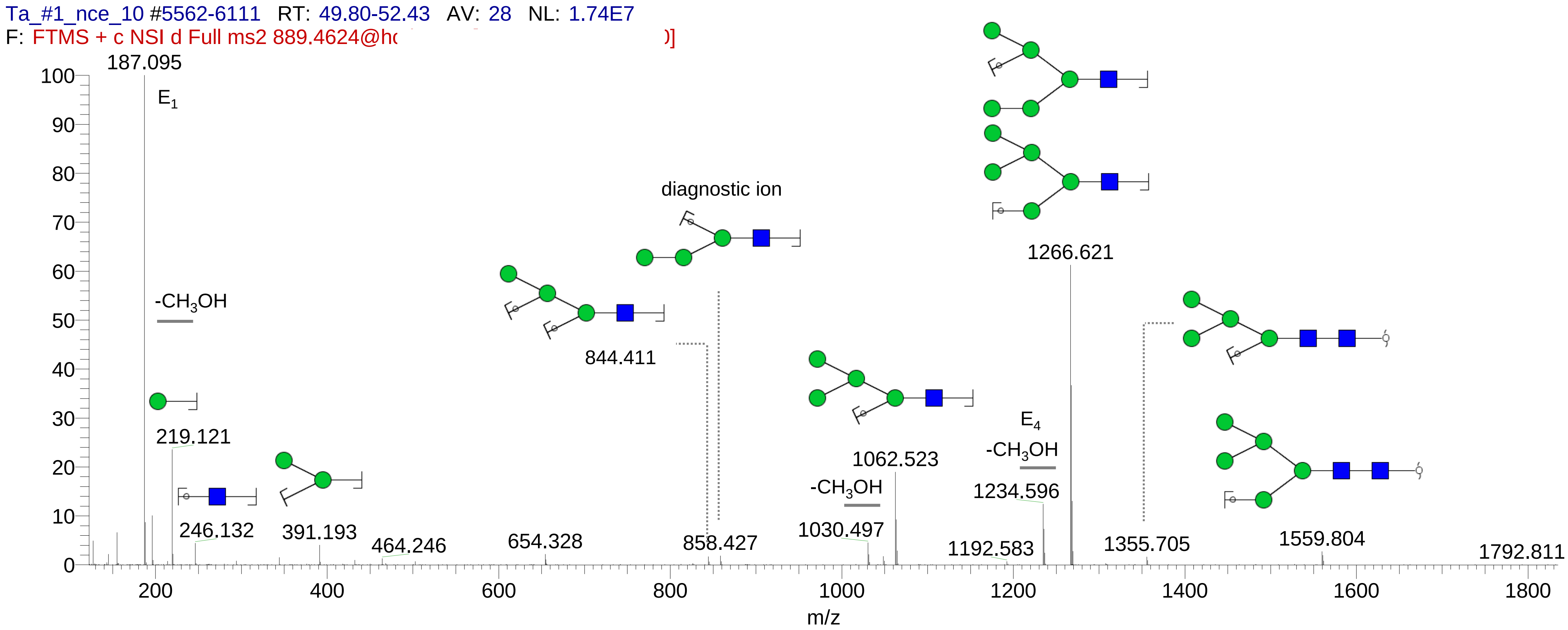

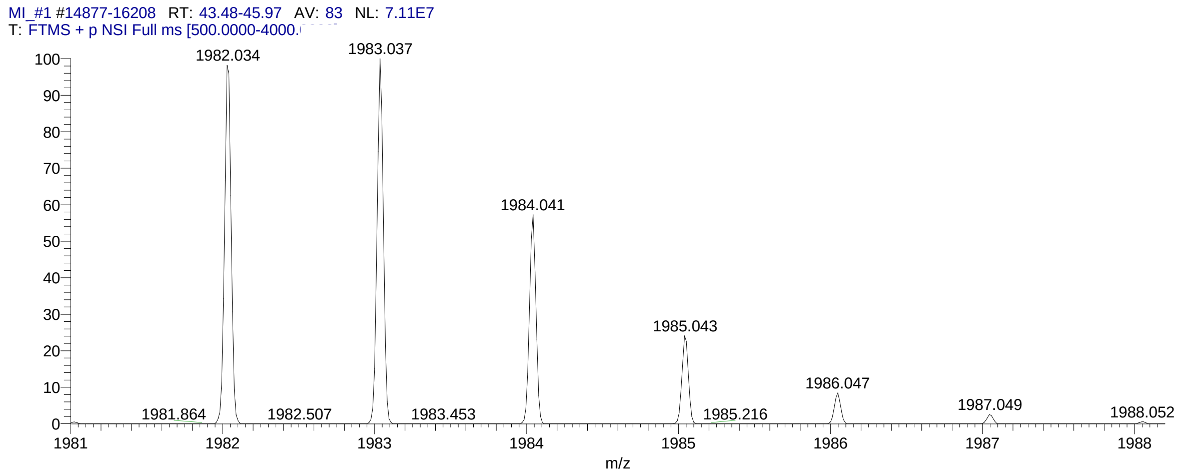

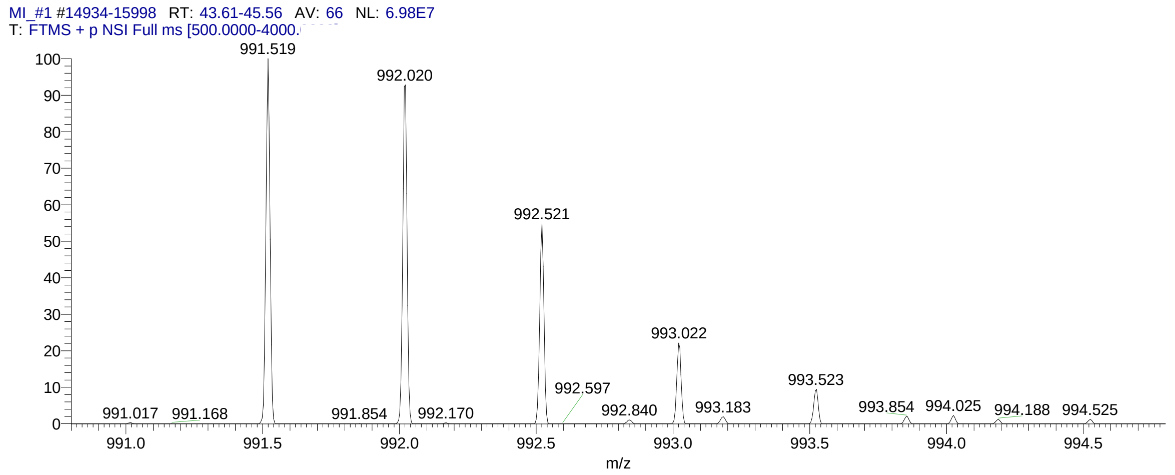

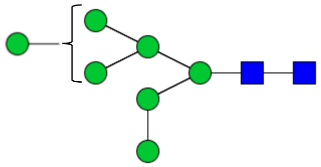

Glycan #4

HexNAc(2)Hex(7)

Permethylated, reduced

Theoretical *m/z* 1982.0315 (z=1)

Theoretical *m/z* 991.5194 (z=2)

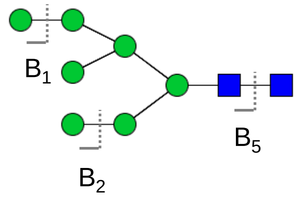

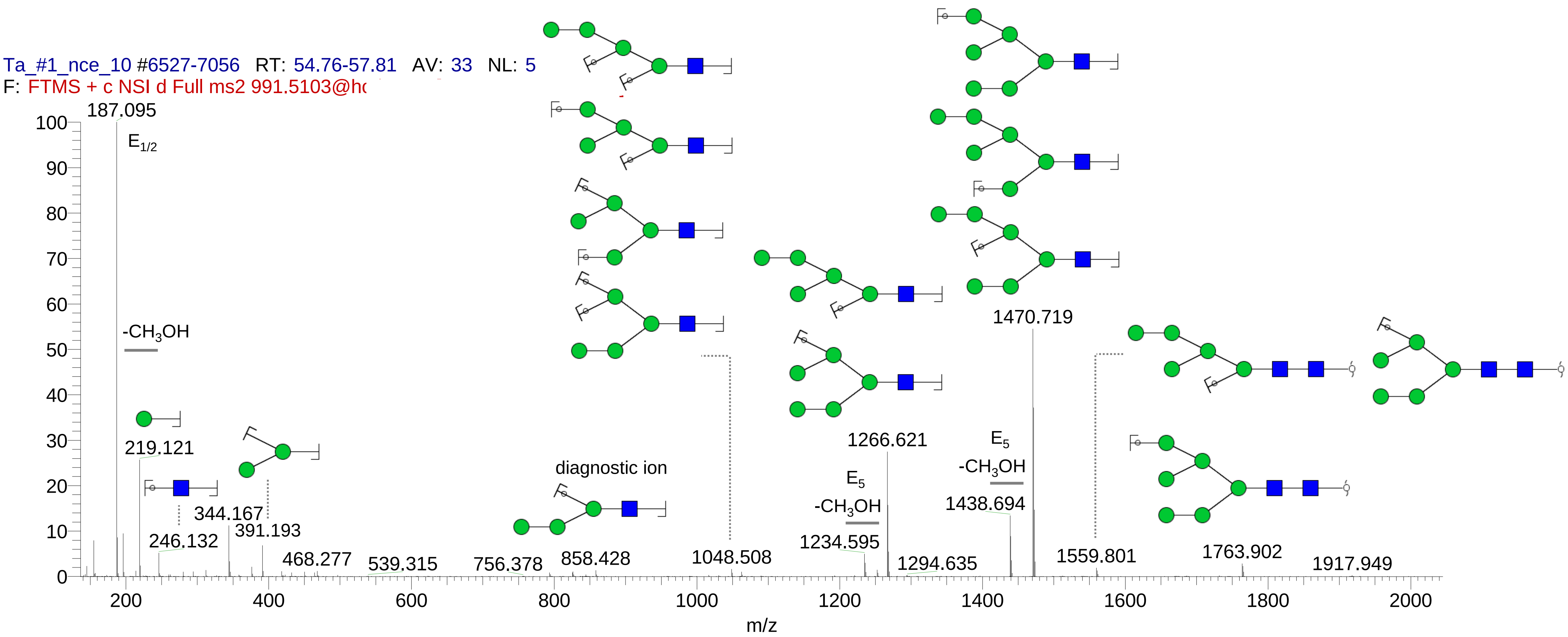

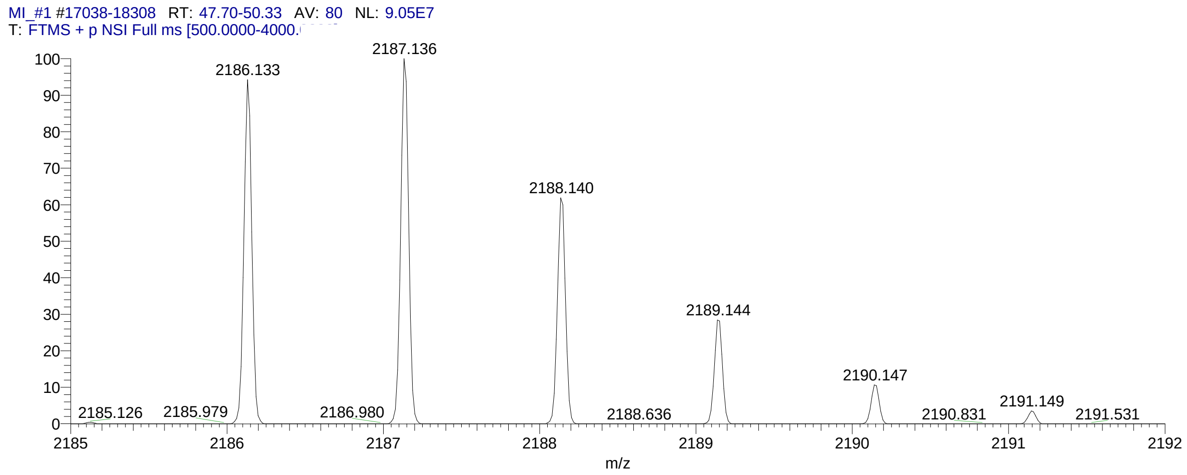

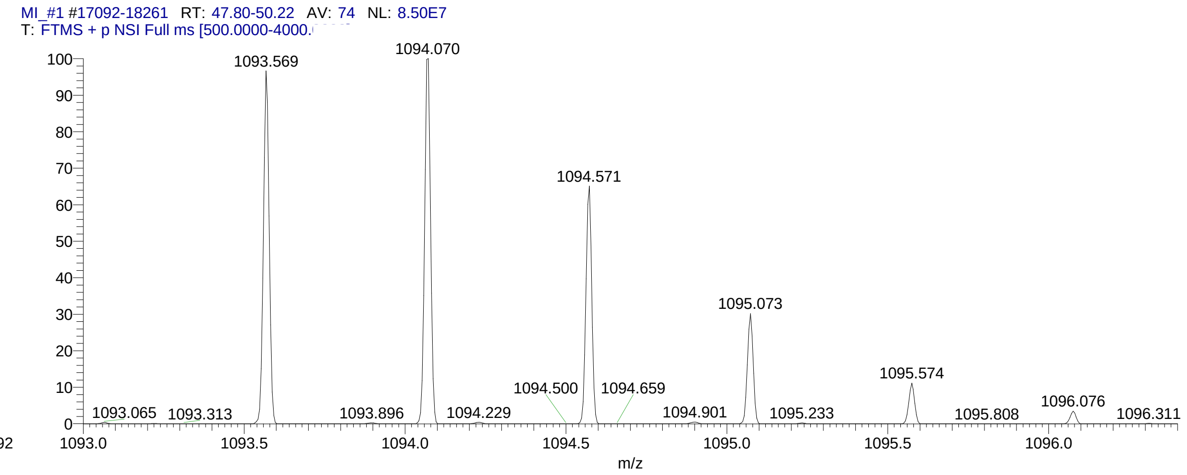

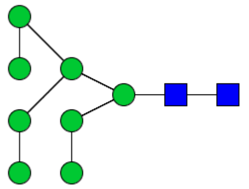

Glycan #5

HexNAc(2)Hex(8)

Permethylated, reduced

Theoretical *m/z* 2186.1313 (z=1)

Theoretical *m/z* 1093.5693 (z=2)

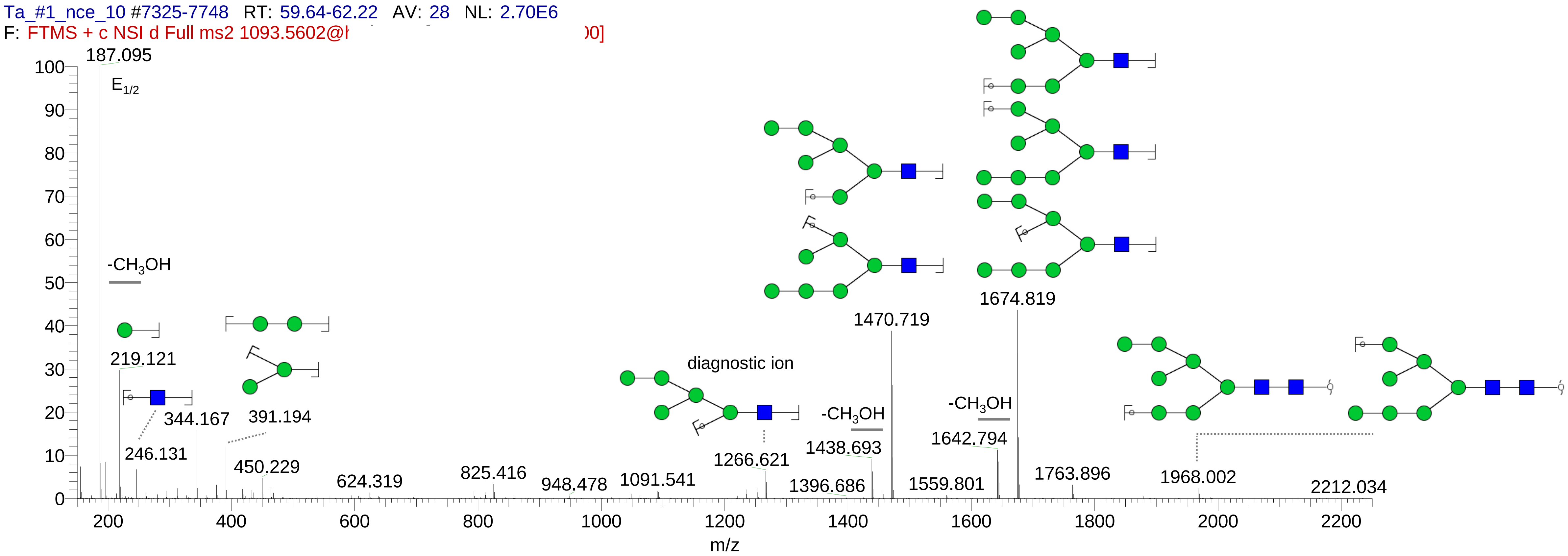

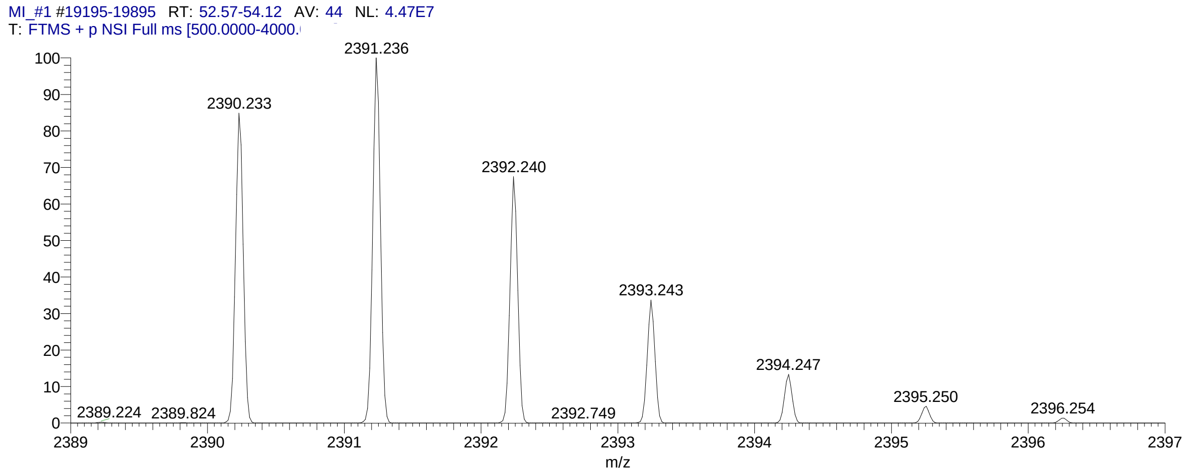

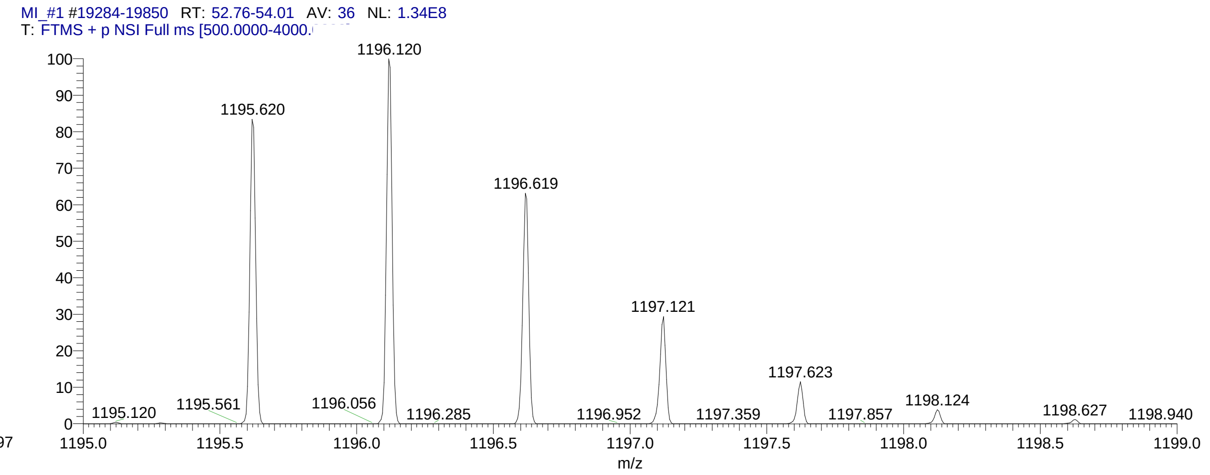

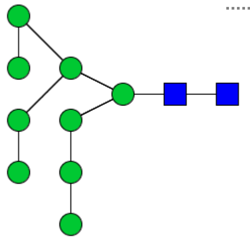

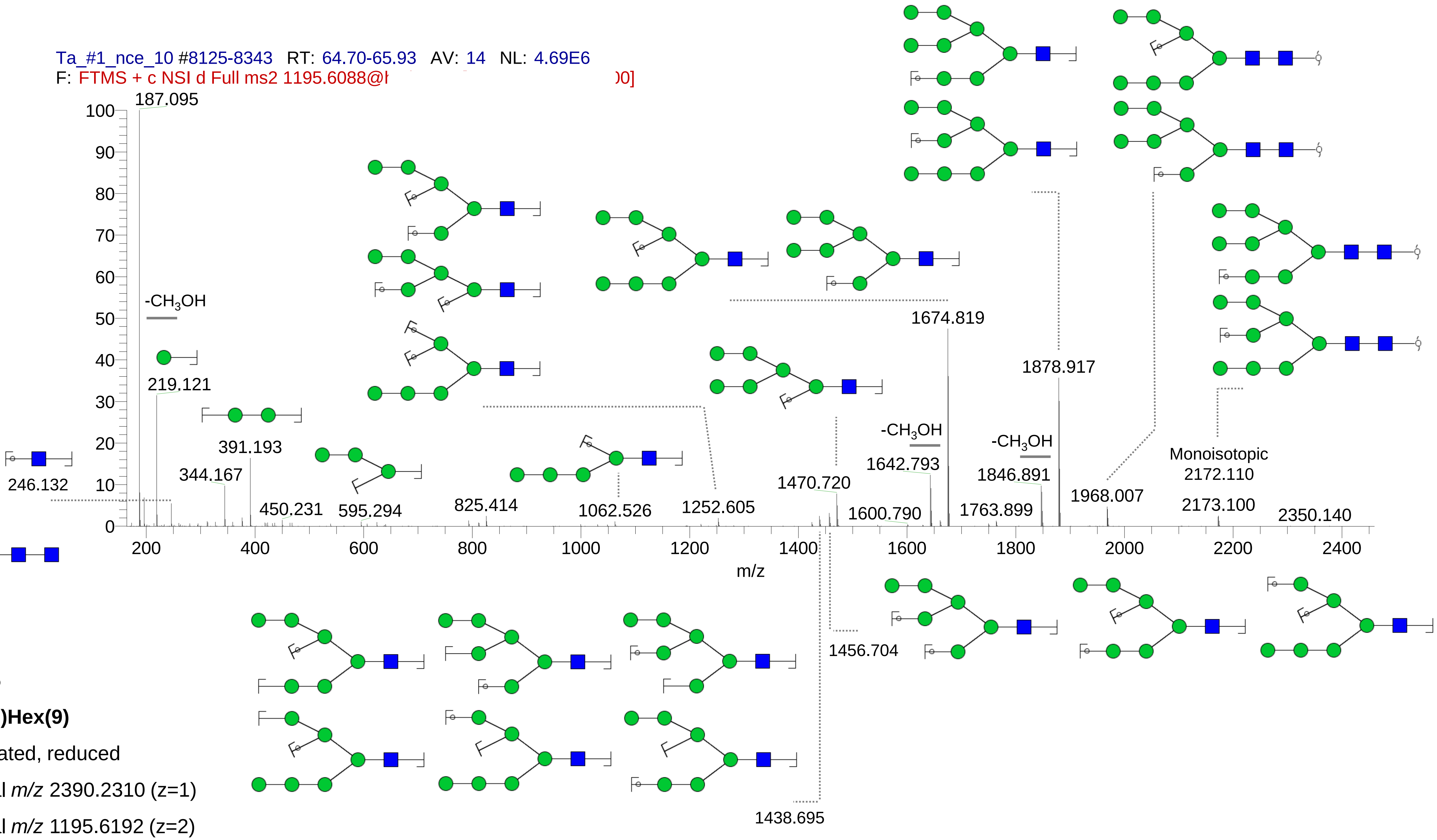

Glycan #6

HexNAc(2)Hex(9)

Permethylated, reduced

Theoretical *m/z* 2390.2310 (z=1)

Theoretical *m/z* 1195.6192 (z=2)

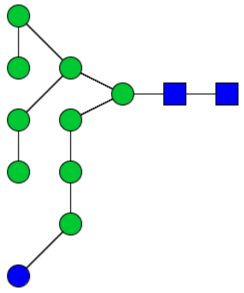

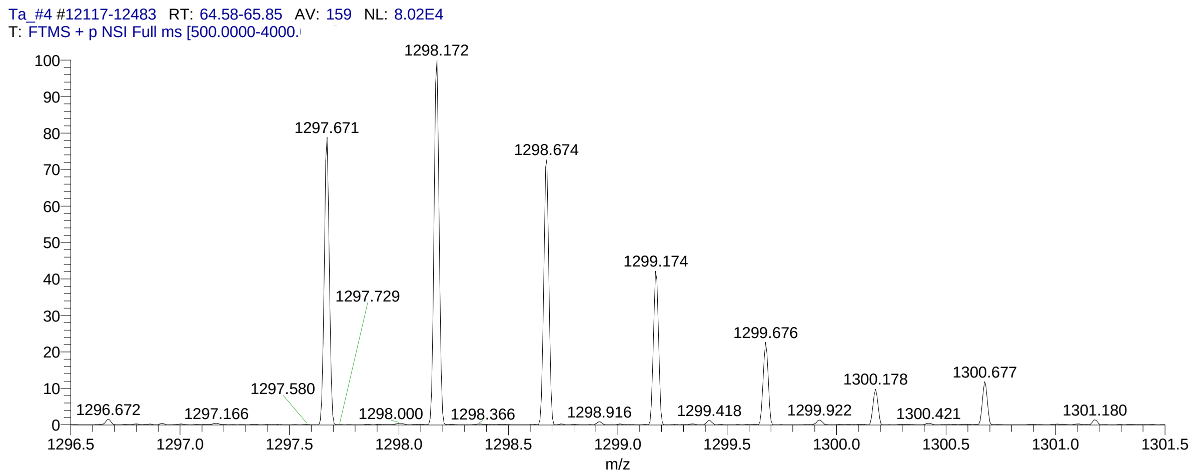

Glycan #7

HexNAc(2)Hex(10)

Permethylated, reduced

Theoretical *m/z* 1297.6690 (z=2)

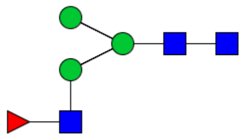

Glycan #8a

HexNAc(3)Hex(3)Fuc(1)

Permethylated, reduced

Theoretical m/z 1584.8479 (z=1)

Theoretical m/z 792.9276 (z=2)

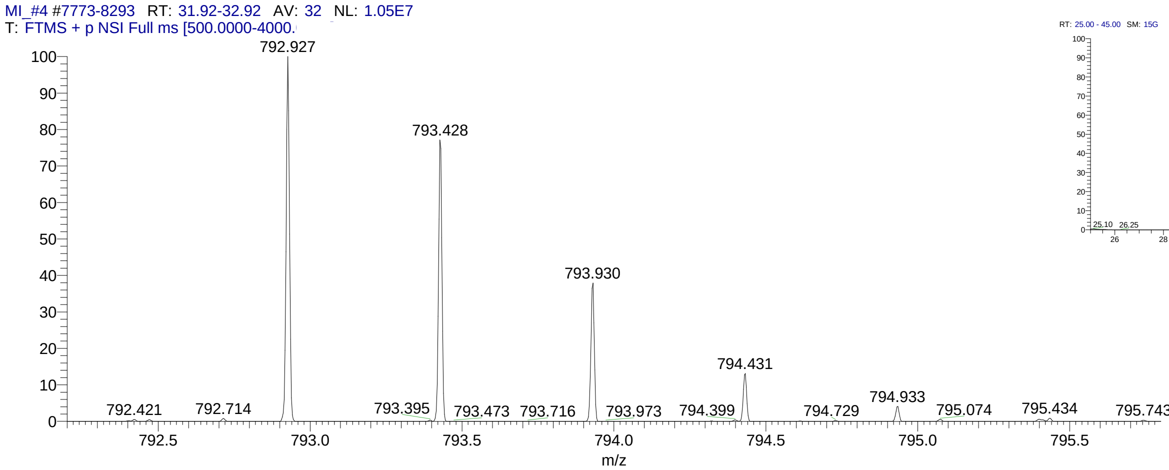

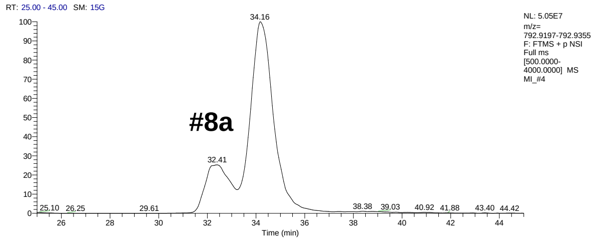

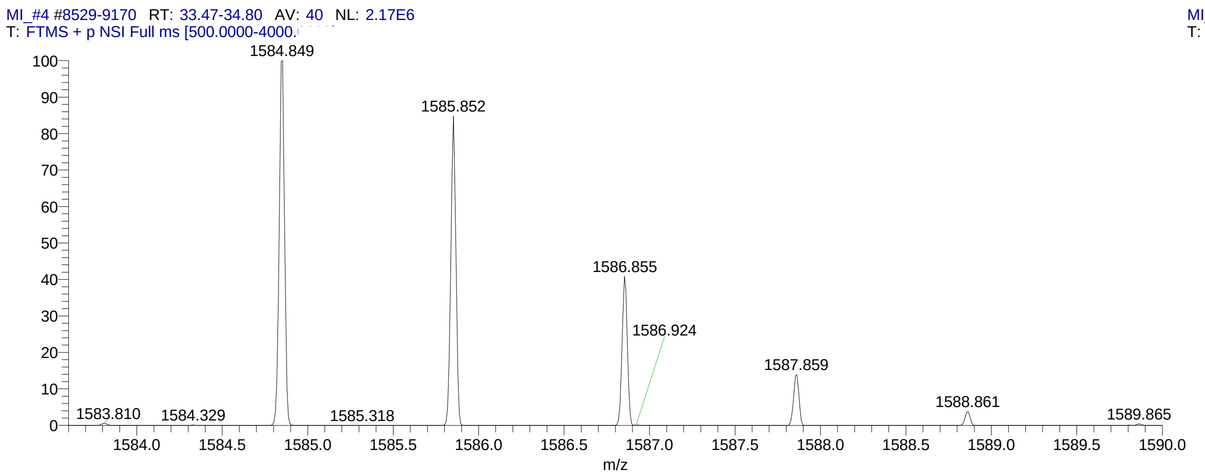

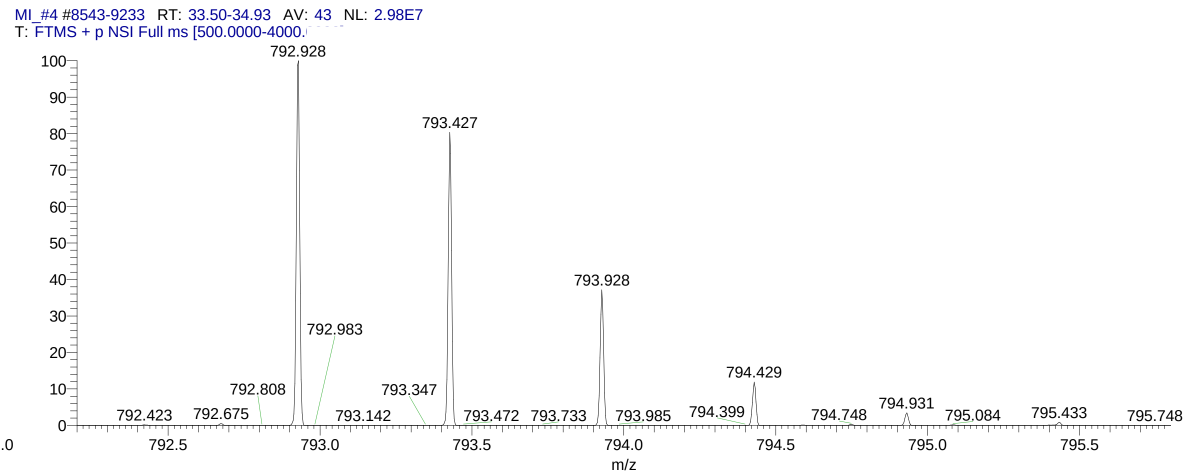

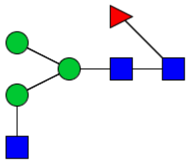

Glycan #8b

HexNAc(3)Hex(3)Fuc(1)

Permethylated, reduced

Theoretical m/z 1584.8479 (z=1)

Theoretical m/z 792.9276 (z=2)

#8b

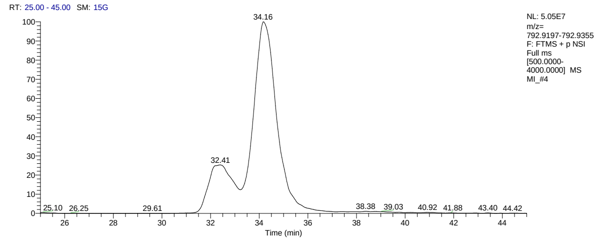

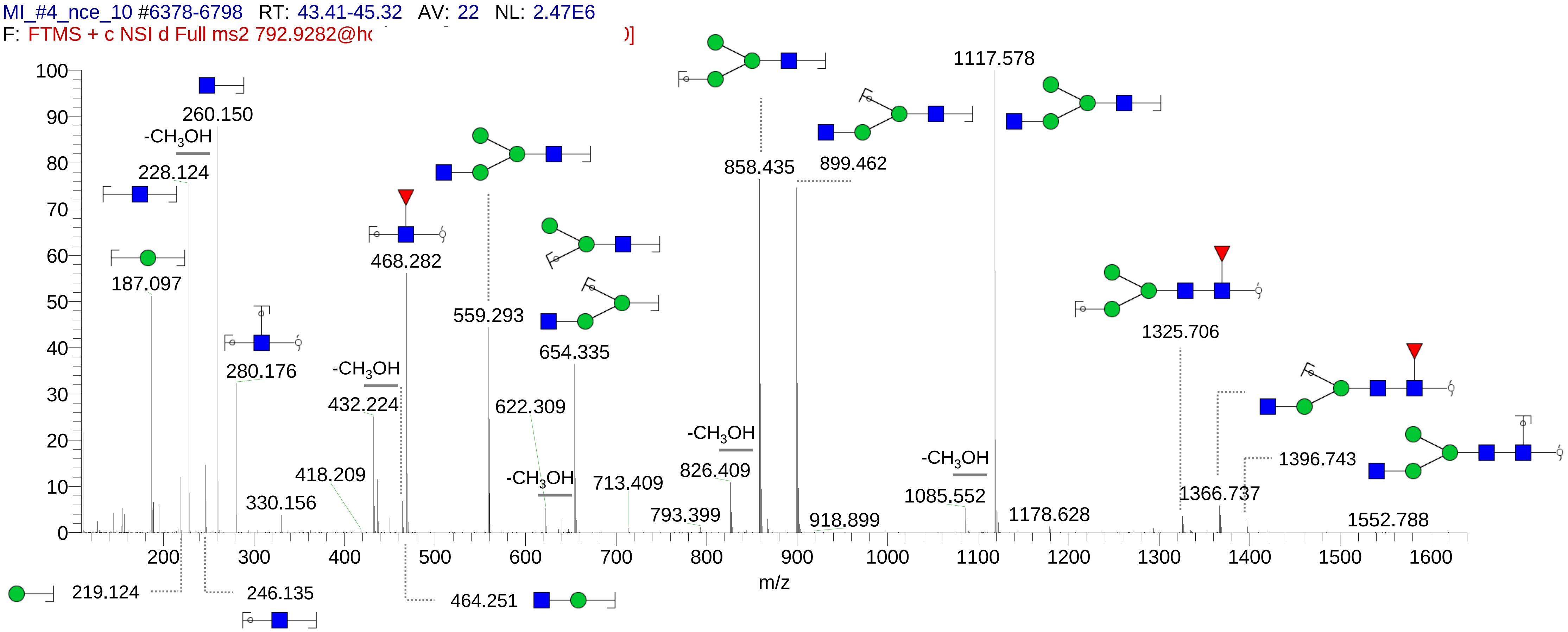

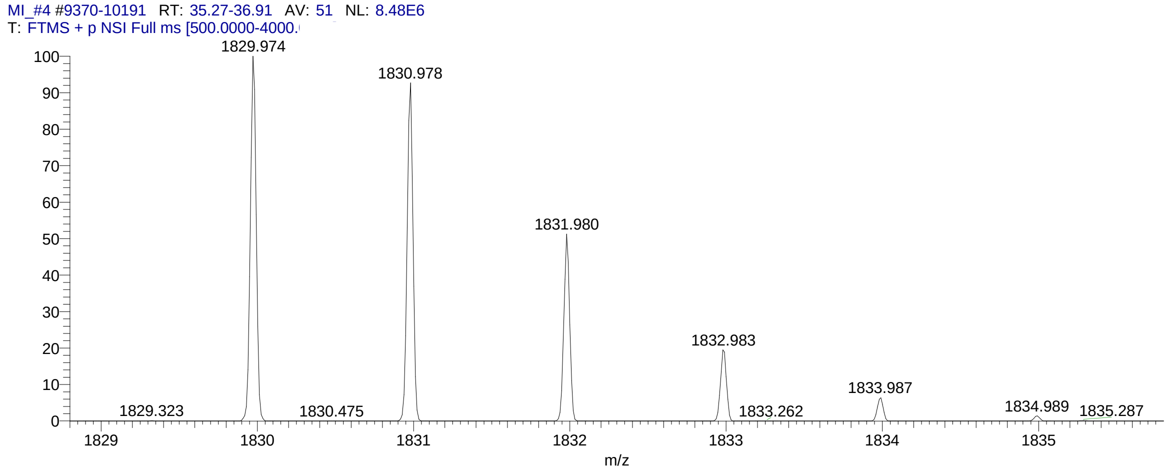

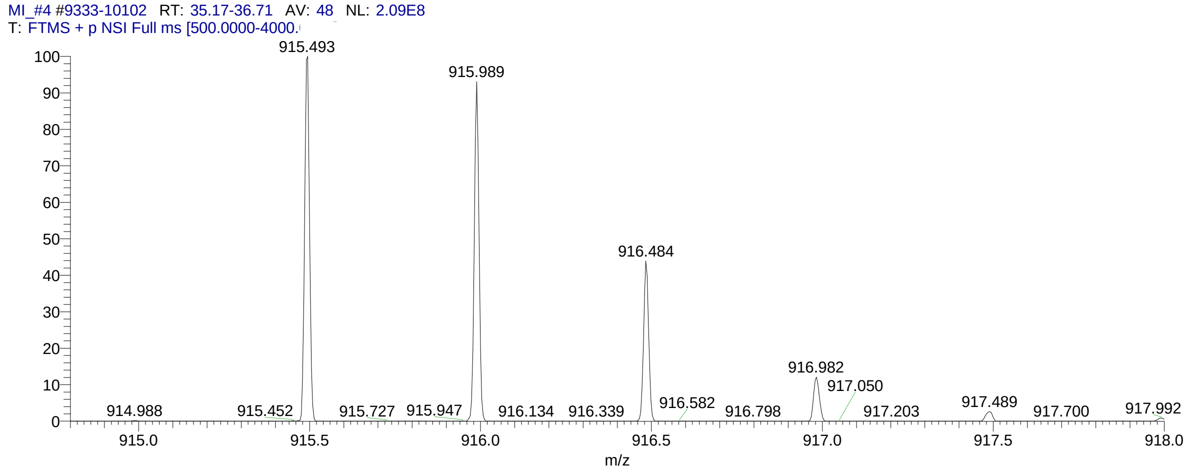

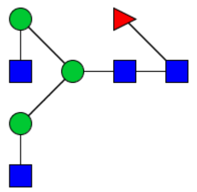

Glycan #9

HexNAc(4)Hex(3)Fuc(1)

Permethylated, reduced

Theoretical m/z 1829.9743 (z=1)

Theoretical m/z 915.4908 (z=2)

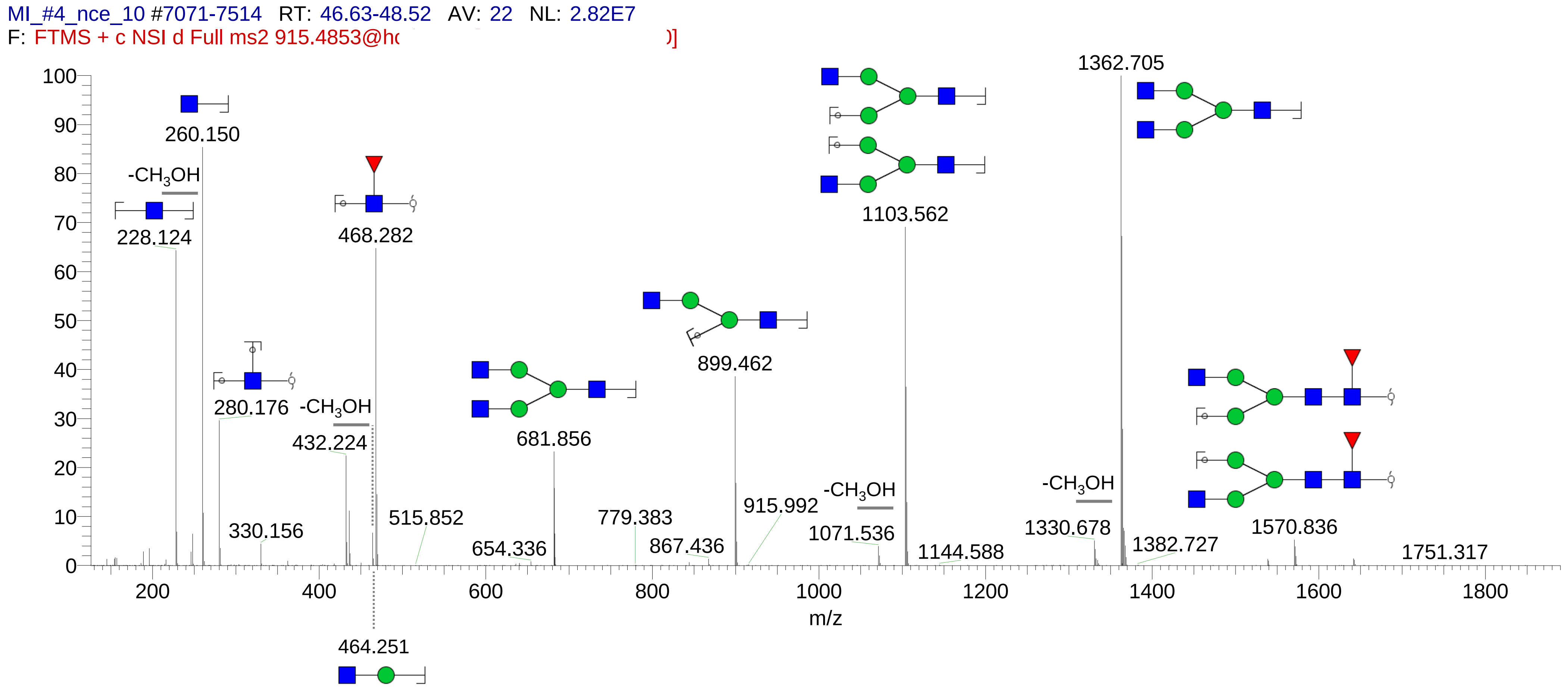

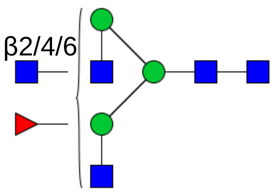

Glycan #10a

HexNAc(5)Hex(3)Fuc(1)

Permethylated, reduced

Theoretical m/z 2075.1006 (z=1)

Theoretical m/z 1038.0539 (z=2)

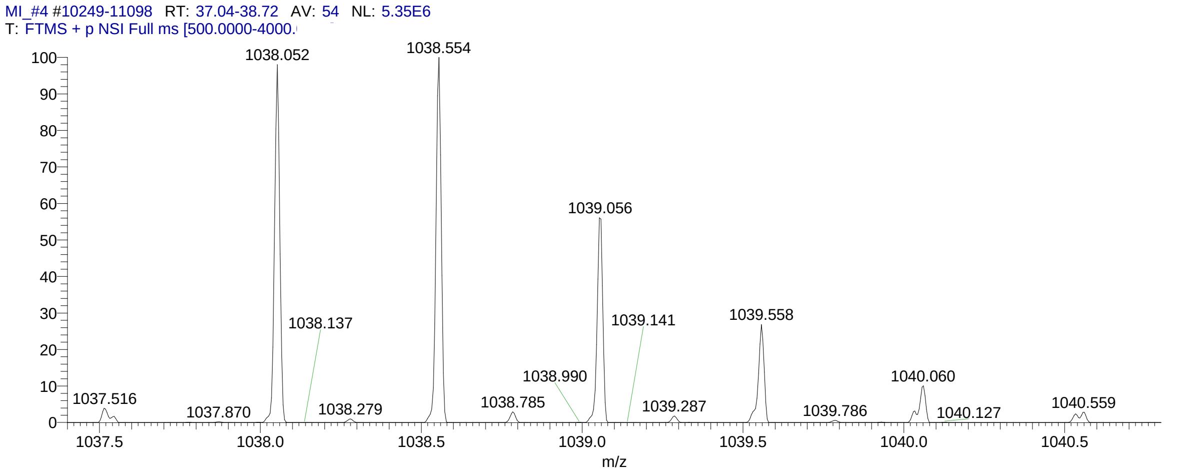

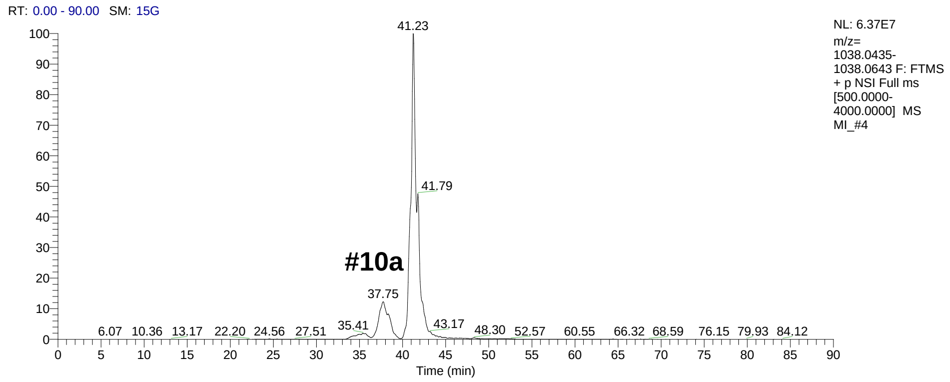

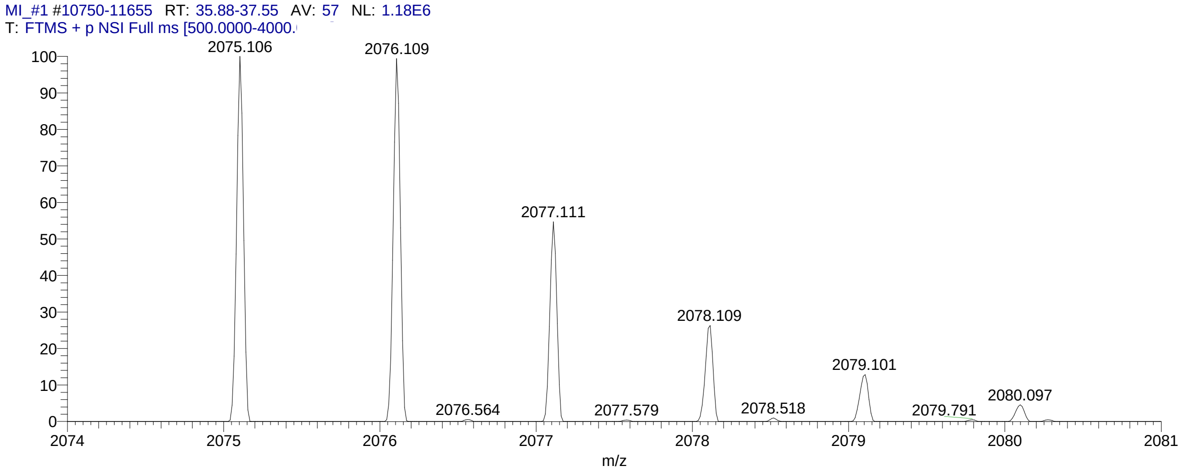

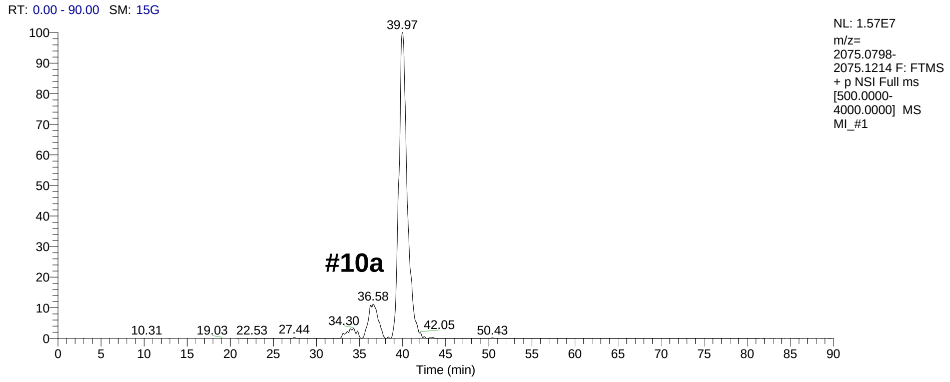

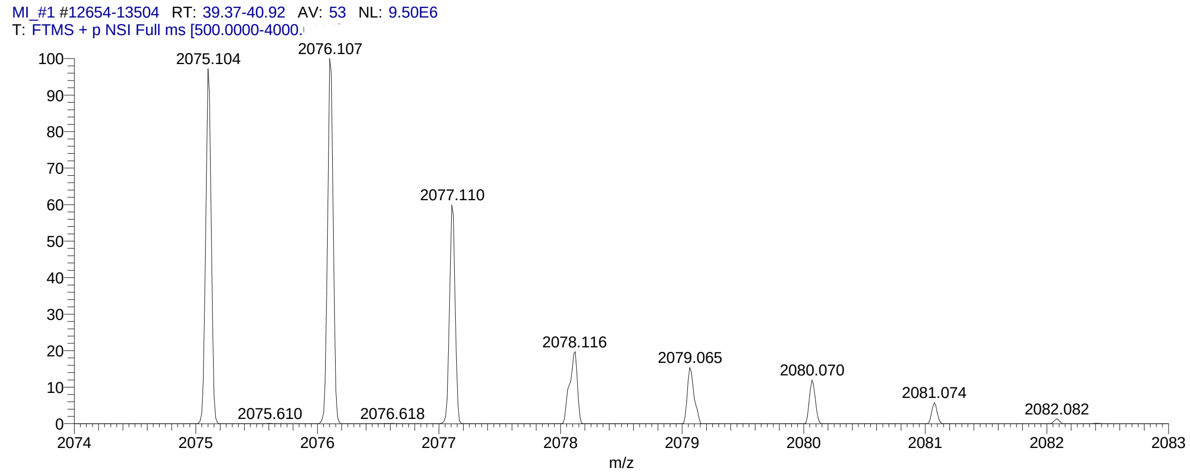

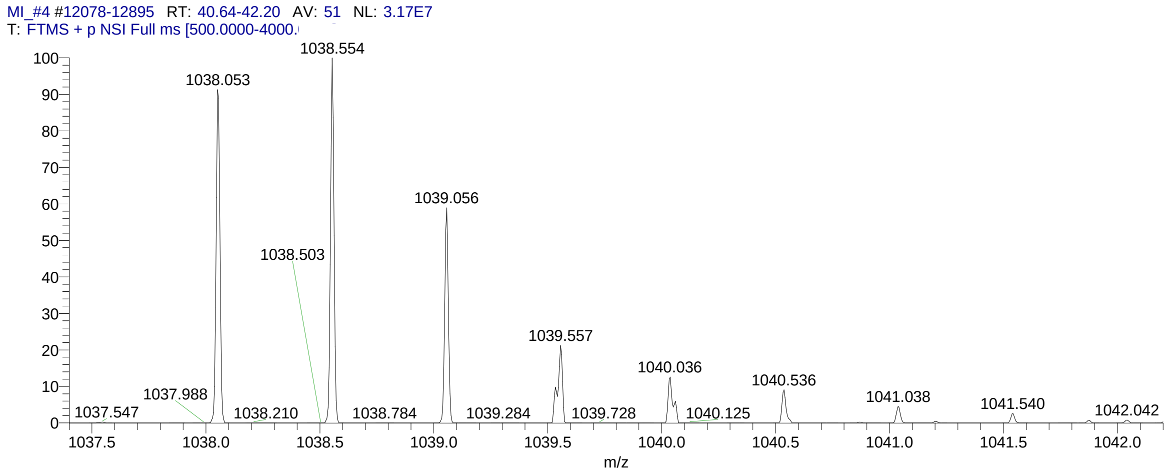

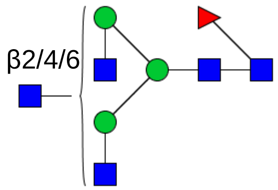

Glycan #10b

HexNAc(5)Hex(3)Fuc(1)

Permethylated, reduced

Theoretical m/z 2075.1006 (z=1)

Theoretical m/z 1038.0539 (z=2)

#10b

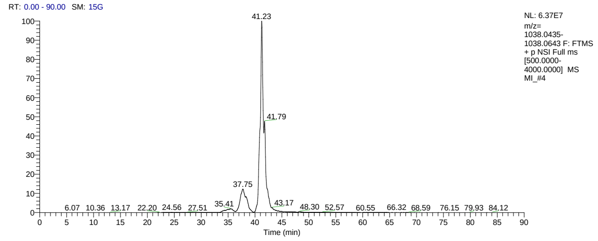

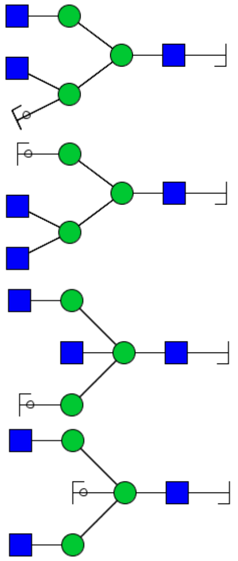

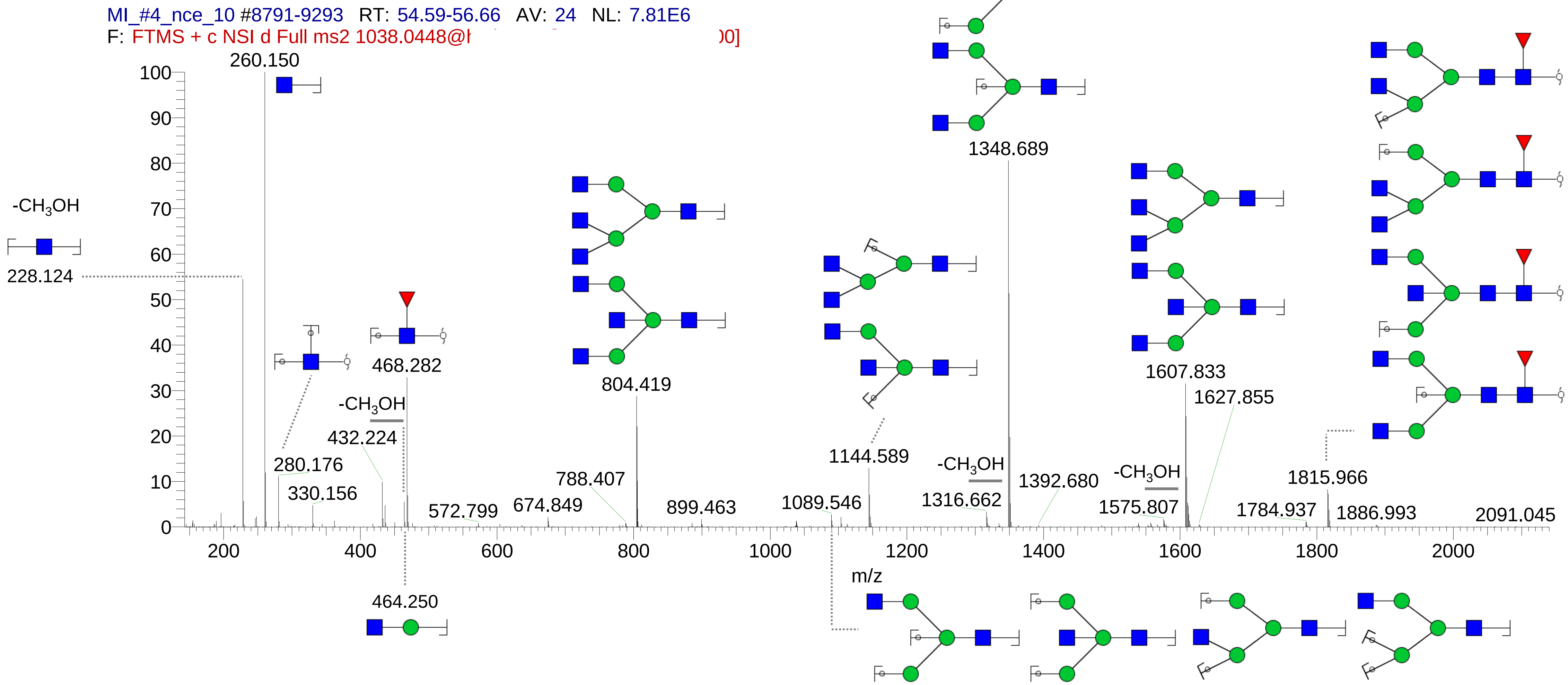

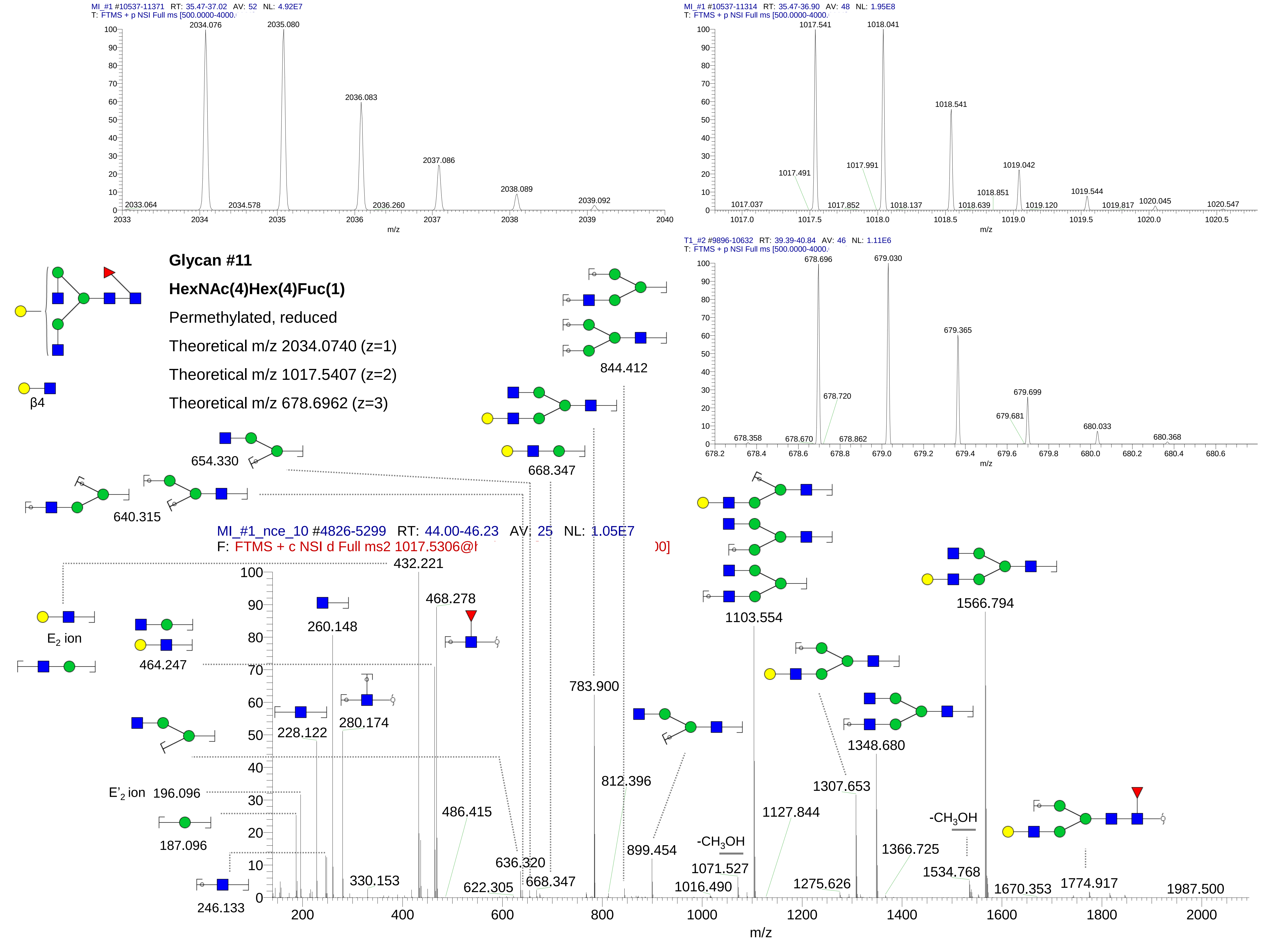
MI_#1 #10537-11371 RT: 35.47-37.02 AV: 52 NL: 4.92E7
T: FTMS + p NSI Full ms [500.0000-4000.
2034.076
2035.080
2036.083
2037.086
2038.089
2039.092
2033.064
2034.578
2036.260
m/z
MI_#1 #10537-11314 RT: 35.47-36.90 AV: 48 NL: 1.95E8
T: FTMS + p NSI Full ms [500.0000-4000.
1017.541
1018.041
1018.541
1019.042
1017.491
1017.991
1018.851
1019.544
1020.045
1020.547
1017.037
1017.852
1018.137
1018.639
1019.120
1019.817
m/z
T1_#2 #9896-10632 RT: 39.39-40.84 AV: 46 NL: 1.11E6
T: FTMS + p NSI Full ms [500.0000-4000.
678.696
679.030
679.365
678.720
679.699
679.681
680.033
680.368
678.358
678.670
678.862
m/z
Glycan #11
HexNAc(4)Hex(4)Fuc(1)
Permethylated, reduced
Theoretical m/z 2034.0740 (z=1)
Theoretical m/z 1017.5407 (z=2)
Theoretical m/z 678.6962 (z=3)
β4
844.412
654.330
668.347
640.315
MI_#1_nce_10 #4826-5299 RT: 44.00-46.23 AV: 25 NL: 1.05E7
F: FTMS + c NSI d Full ms2 1017.5306@
432.221
468.278
260.148
E2 ion
464.247
783.900
280.174
228.122
812.396
486.415
E'2 ion 196.096
187.096
636.320
668.347
622.305
330.153
246.133
899.454
1103.554
1566.794
1348.680
1307.653
1127.844
1366.725
1534.768
-CH3OH
1071.527
1016.490
1275.626
1670.353
1774.917
1987.500
m/z

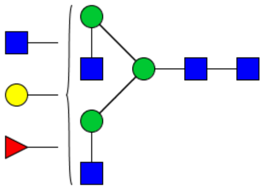

Glycan #12

HexNAc(5)Hex(4)Fuc(1)

Permethylated, reduced

Theoretical m/z 1140.1038 (z=2)

Theoretical m/z 760.4050 (z=3)

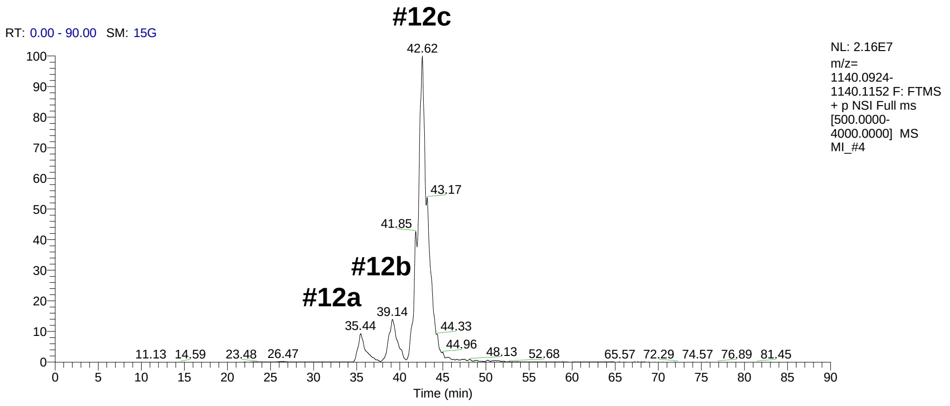

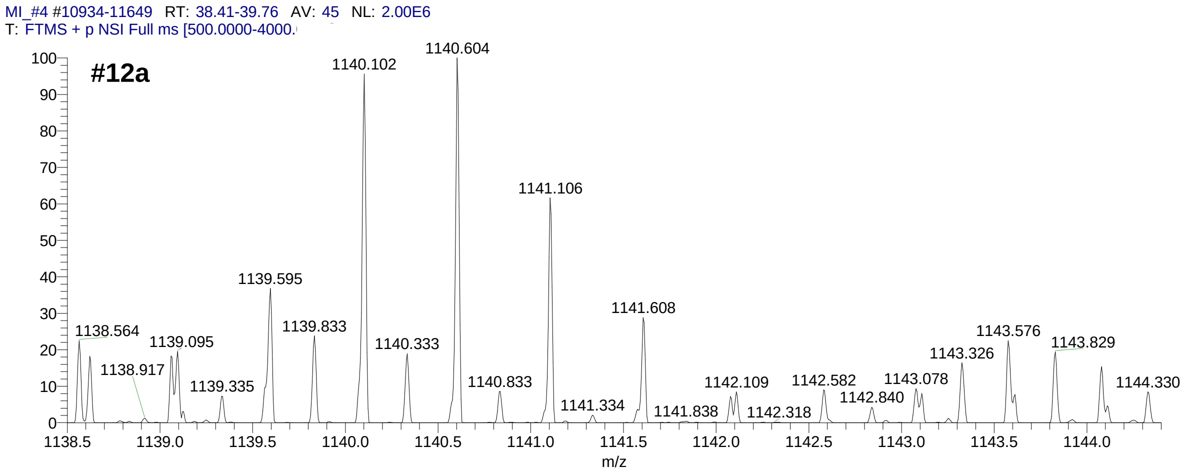

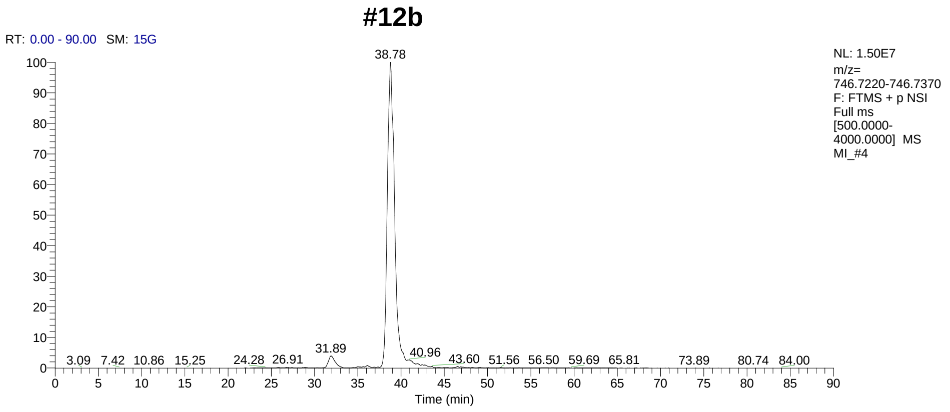

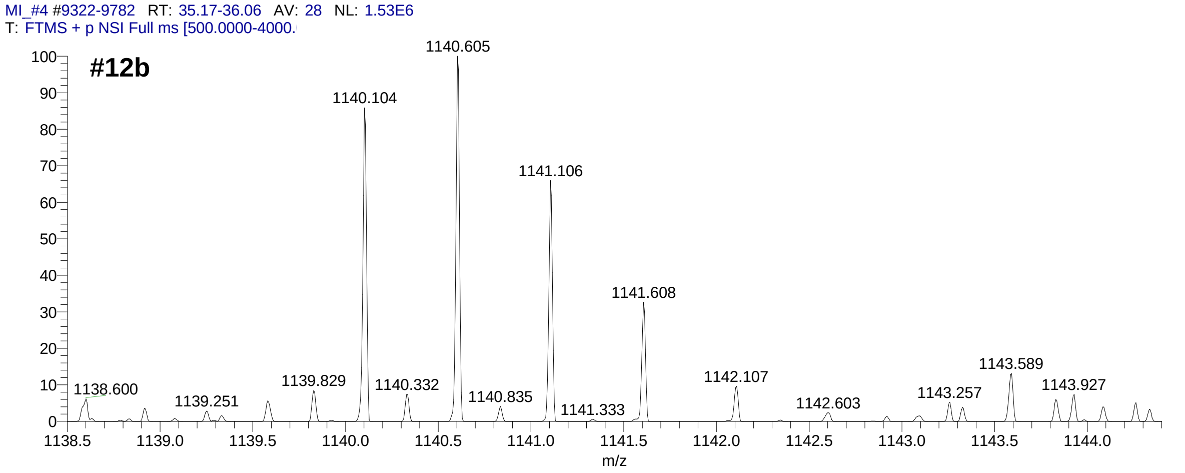

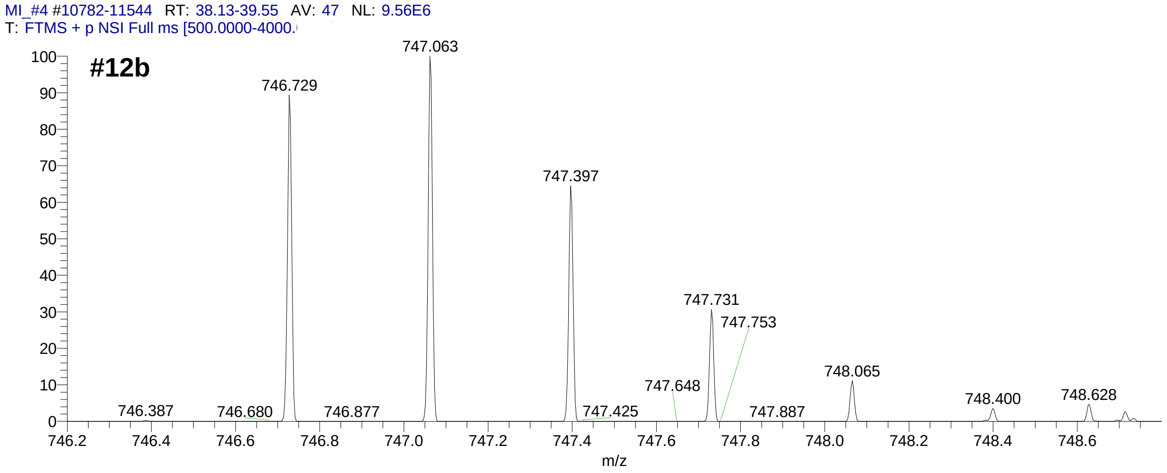

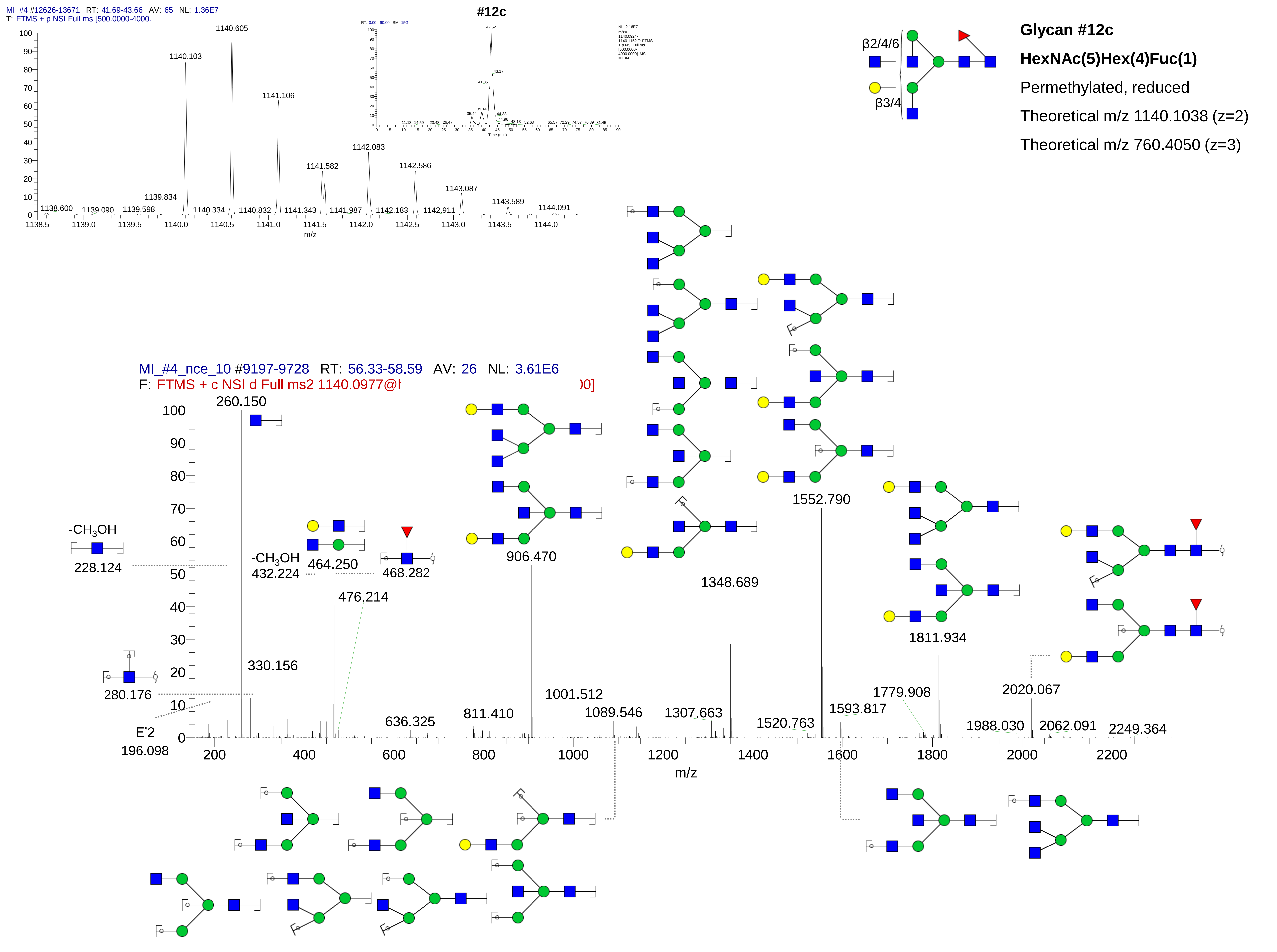

#12c
Glycan #12c
HexNAc(5)Hex(4)Fuc(1)
Permethylated, reduced
Theoretical m/z 1140.1038 (z=2)
Theoretical m/z 760.4050 (z=3)
β2/4/6
β3/4
MI_#4 #12626-13671 RT: 41.69-43.66 AV: 65 NL: 1.36E7
T: FTMS + p NSI Full ms [500.0000-4000.
1140.605
1140.103
1141.106
1142.083
1141.582
1142.586
1143.087
1143.589
1144.091
1139.834
1138.600
1139.090
1139.598
1140.334
1140.832
1141.343
1141.987
1142.183
1142.911
m/z
MI_#4_nce_10 #9197-9728 RT: 56.33-58.59 AV: 26 NL: 3.61E6
F: FTMS + c NSI d Full ms2 1140.0977@
260.150
-CH3OH
228.124
-CH3OH
432.224
464.250
468.282
476.214
330.156
280.176
E'2
196.098
636.325
811.410
906.470
1001.512
1089.546
1307.663
1348.689
1520.763
1552.790
1593.817
1779.908
1811.934
1988.030
2020.067
2062.091
2249.364
m/z

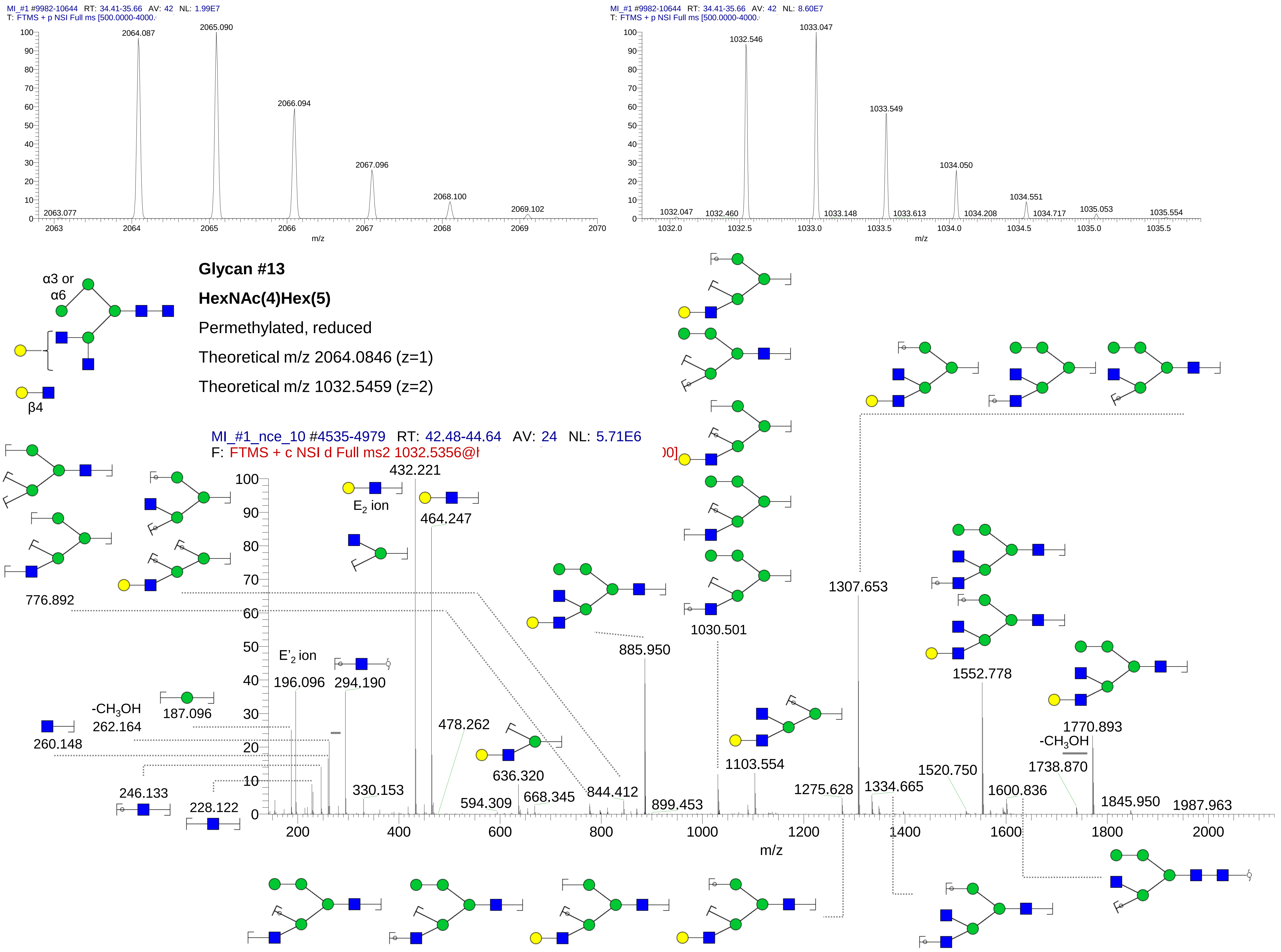

MI_#1 #9982-10644 RT: 34.41-35.66 AV: 42 NL: 1.99E7
T: FTMS + p NSI Full ms [500.0000-4000.
2063.077
2064.087
2065.090
2066.094
2067.096
2068.100
2069.102
m/z
MI_#1 #9982-10644 RT: 34.41-35.66 AV: 42 NL: 8.60E7
T: FTMS + p NSI Full ms [500.0000-4000.
1032.047
1032.460
1032.546
1033.047
1033.148
1033.549
1033.613
1034.050
1034.208
1034.551
1034.717
1035.053
1035.554
m/z
α3 or α6
β4
Glycan #13
HexNAc(4)Hex(5)
Permethylated, reduced
Theoretical m/z 2064.0846 (z=1)
Theoretical m/z 1032.5459 (z=2)
MI_#1_nce_10 #4535-4979 RT: 42.48-44.64 AV: 24 NL: 5.71E6
F: FTMS + c NSI d Full ms2 1032.5356@h
0]
432.221
E$_2$ ion
464.247
776.892
E'$_2$ ion
196.096
294.190
-CH$_3$OH
262.164
187.096
260.148
246.133
228.122
330.153
478.262
636.320
594.309
668.345
844.412
885.950
899.453
1030.501
1103.554
1307.653
1275.628
1334.665
1520.750
1552.778
1600.836
1738.870
1770.893
-CH$_3$OH
1845.950
1987.963
m/z

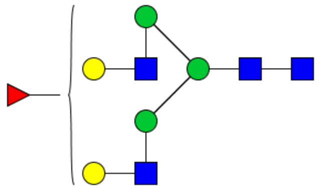

Glycan #14a

HexNAc(4)Hex(5)Fuc(1)

Permethylated, reduced

Theoretical m/z 2238.1738 (z=1)

Theoretical m/z 1119.5905 (z=2)

Theoretical m/z 746.7295 (z=3)

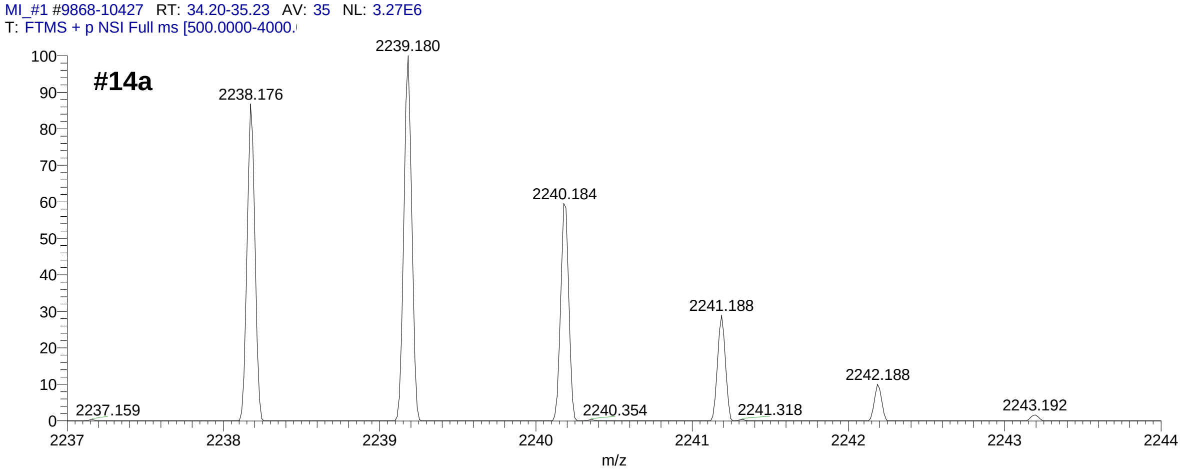

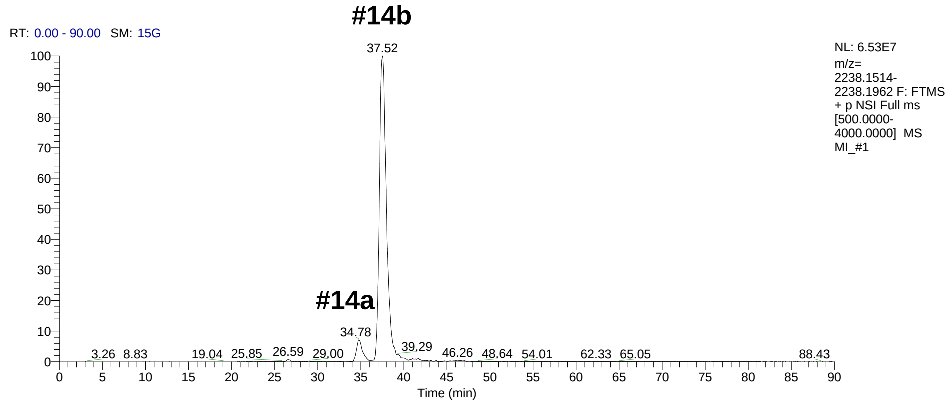

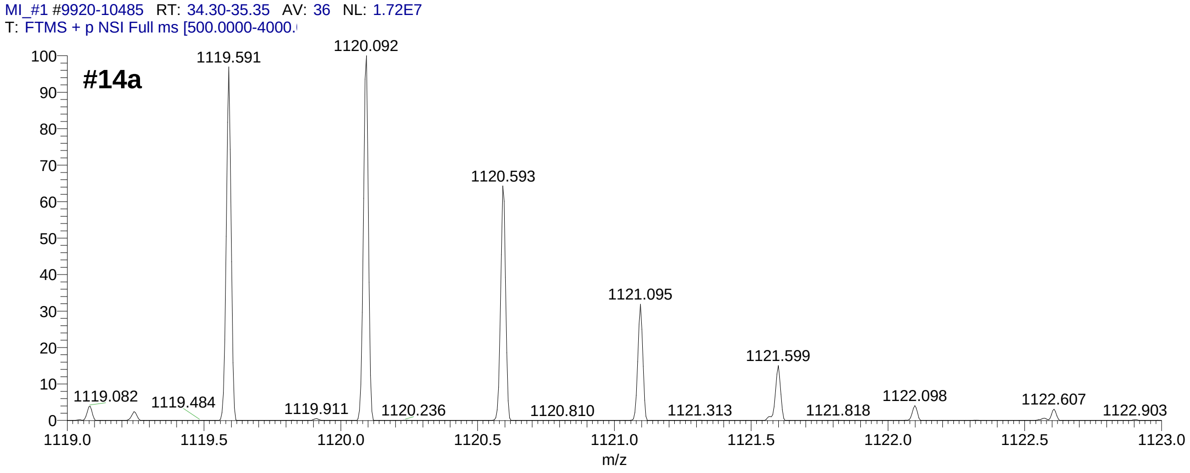

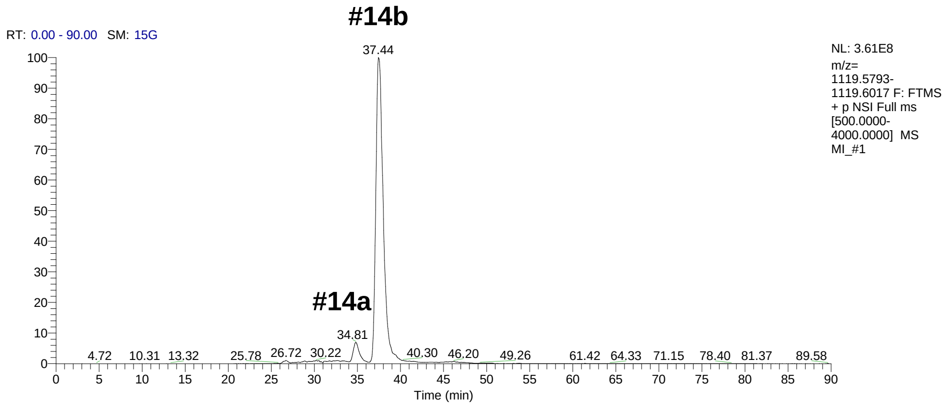

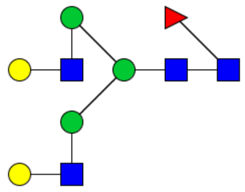

Glycan #14b

HexNAc(4)Hex(5)Fuc(1)

Permethylated, reduced

Theoretical m/z 2238.1738 (z=1)

Theoretical m/z 1119.5905 (z=2)

Theoretical m/z 746.7295 (z=3)

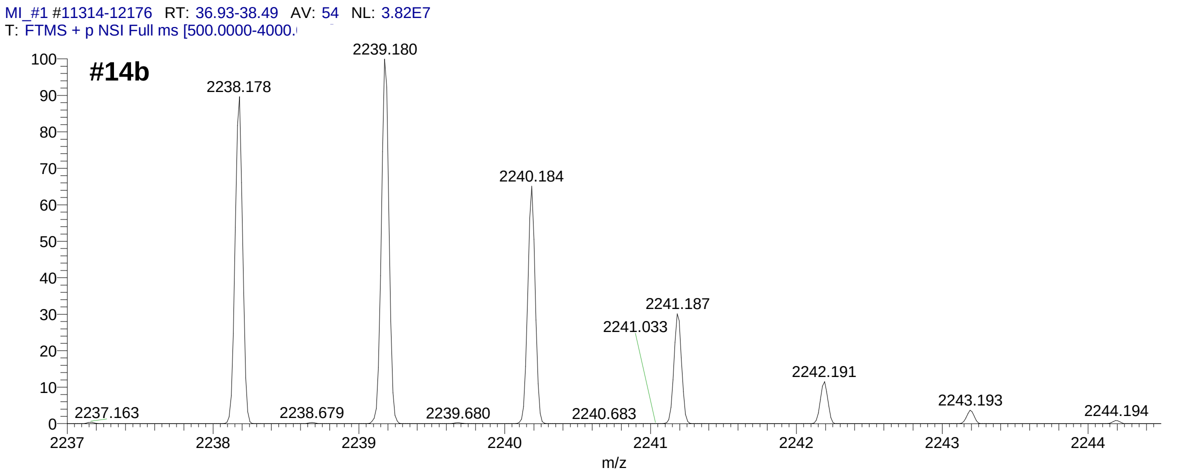

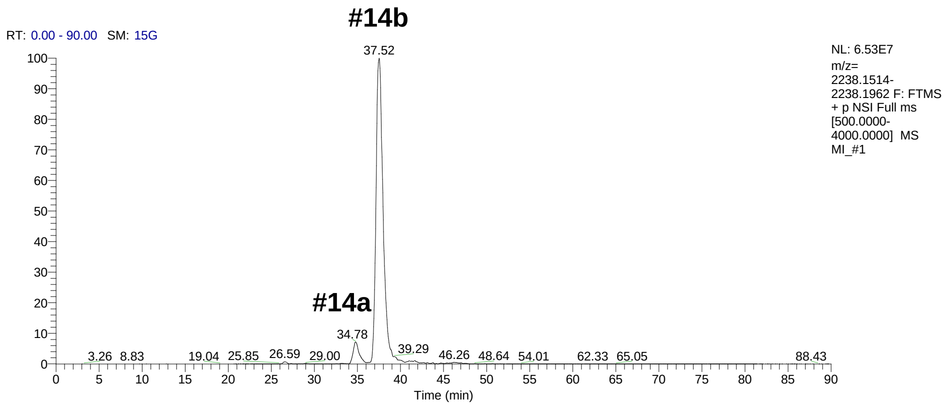

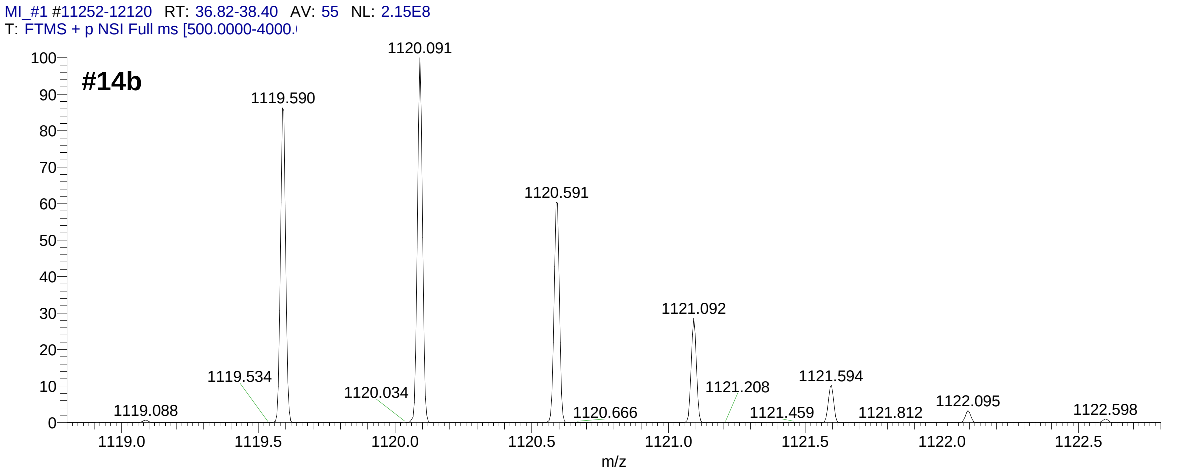

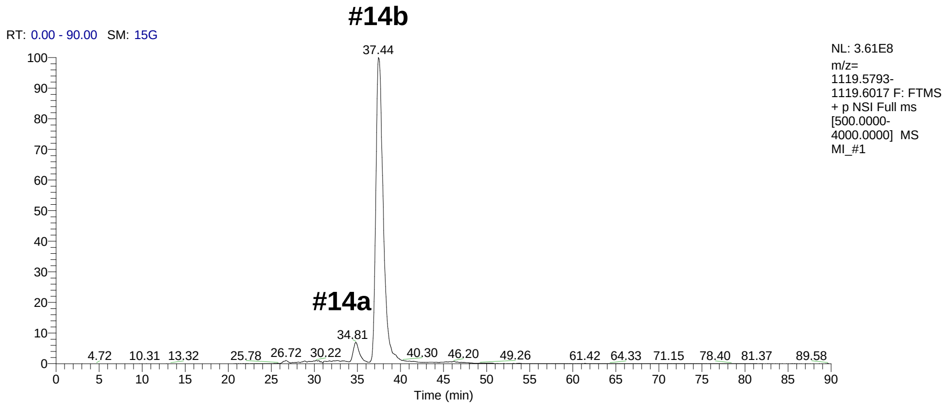

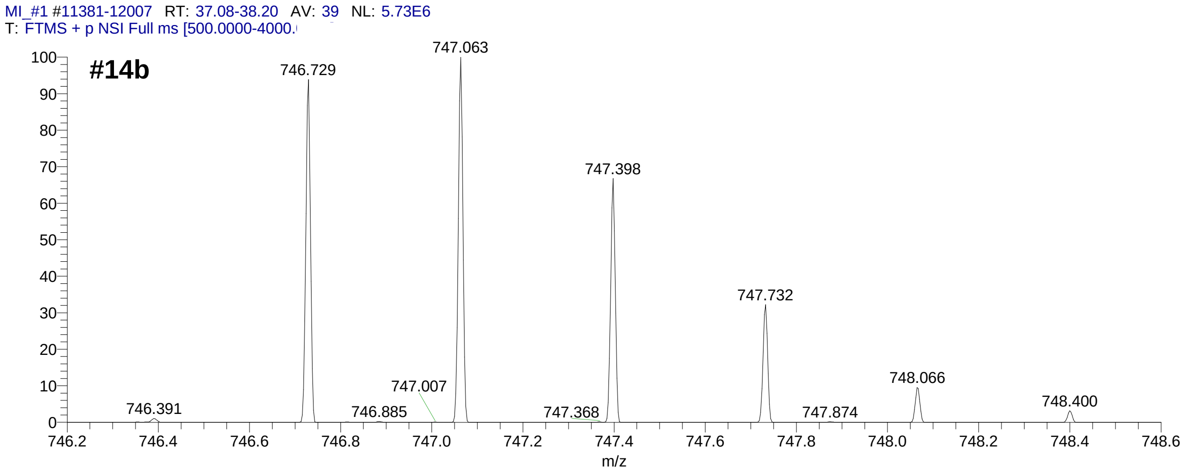

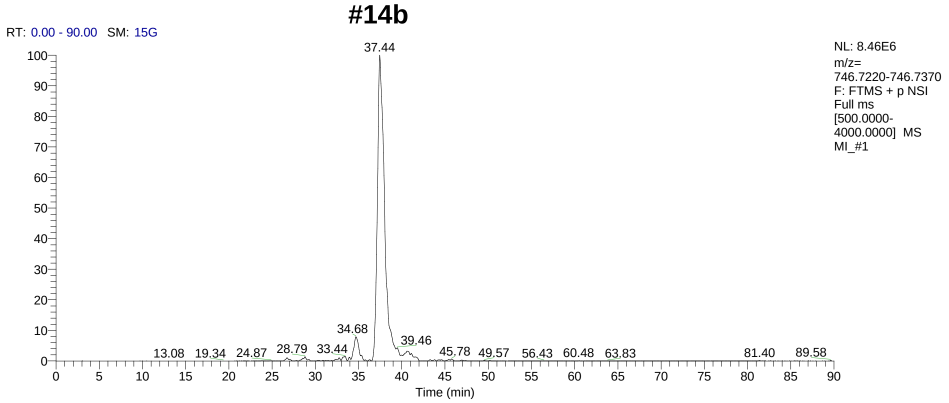

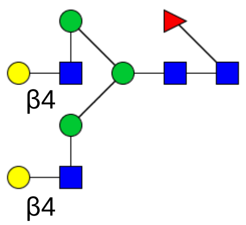

Glycan #14b

HexNAc(4)Hex(5)Fuc(1)

Permethylated, reduced

Theoretical m/z 2238.1738 (z=1)

Theoretical m/z 1119.5905 (z=2)

Theoretical m/z 746.7295 (z=3)

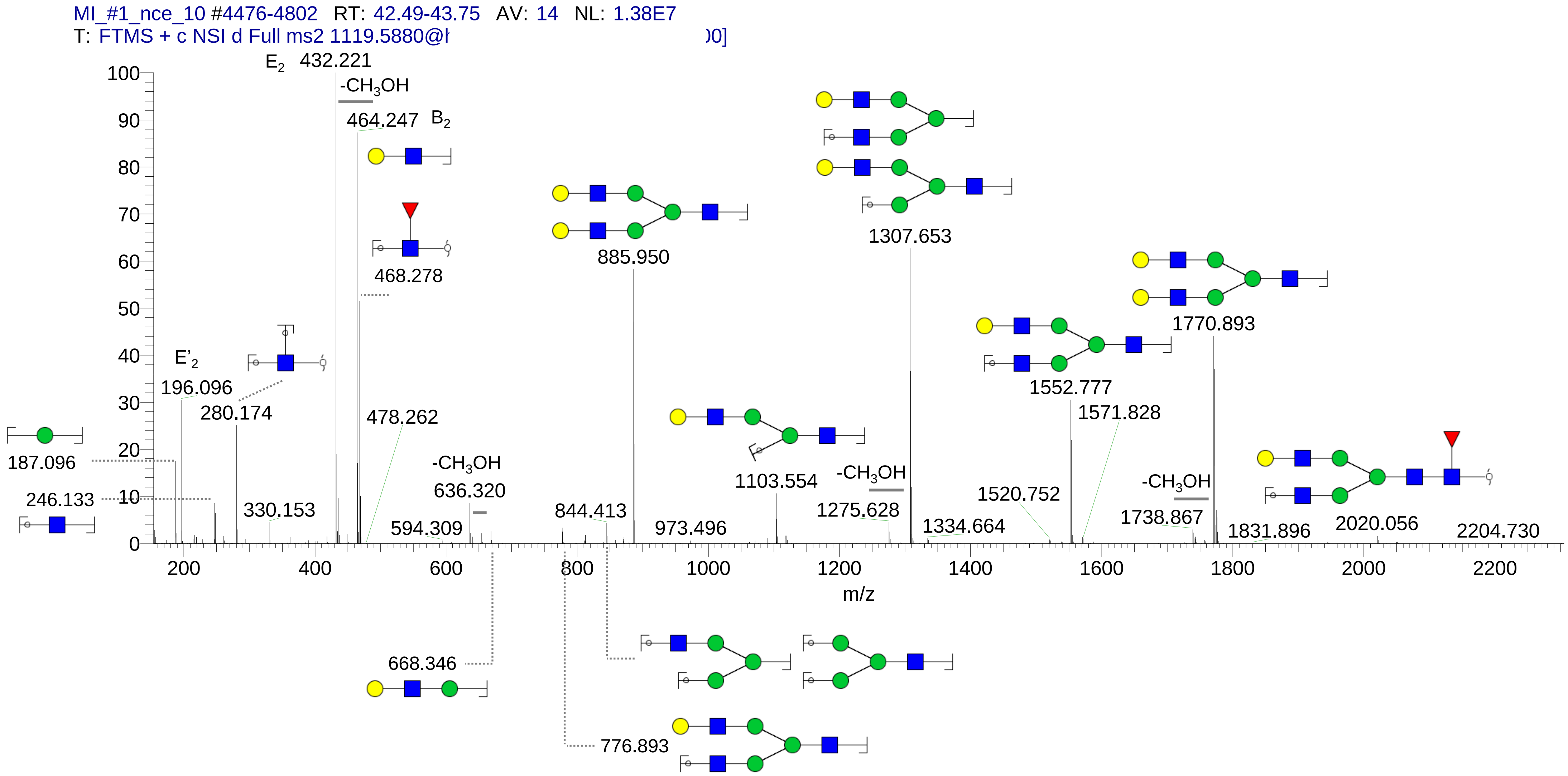

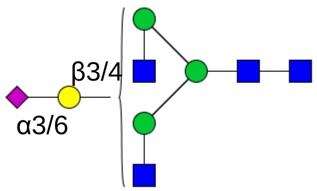

Glycan #15

HexNAc(4)Hex(4)NeuAc(1)

Permethylated, reduced

Theoretical m/z 1111.0829 (z=2)

Theoretical m/z 741.0577 (z=3)

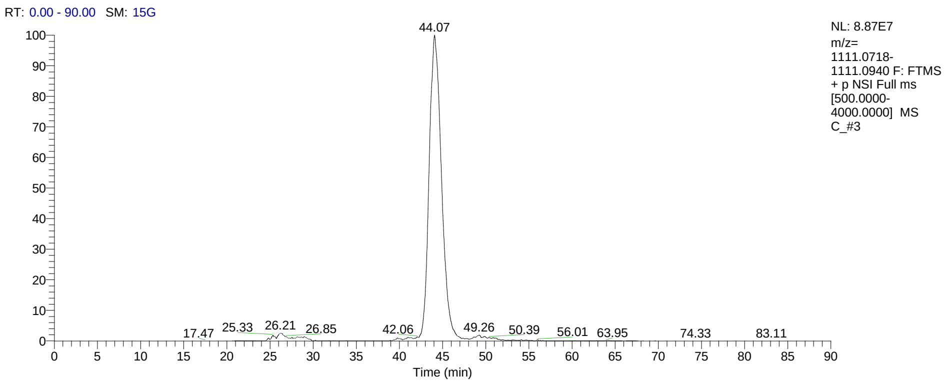

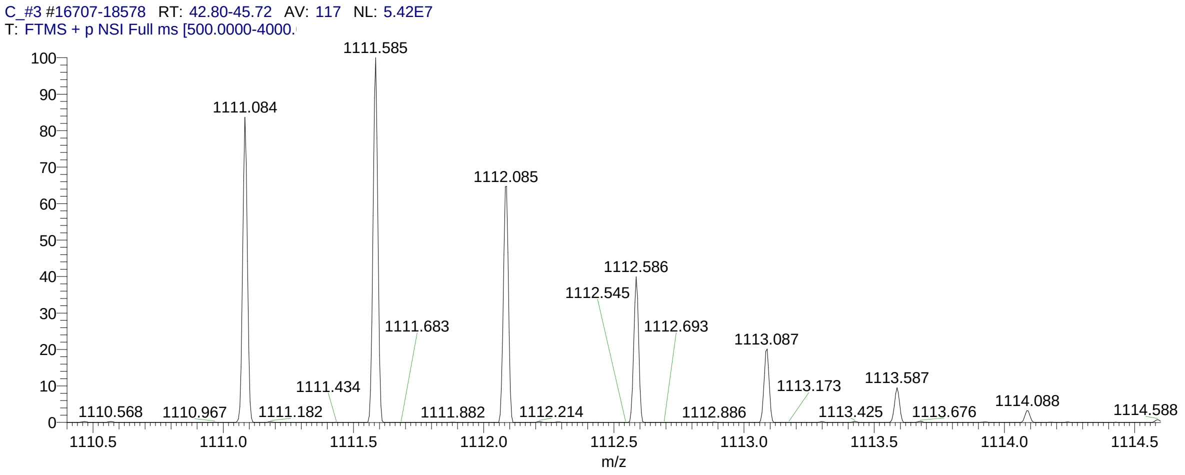

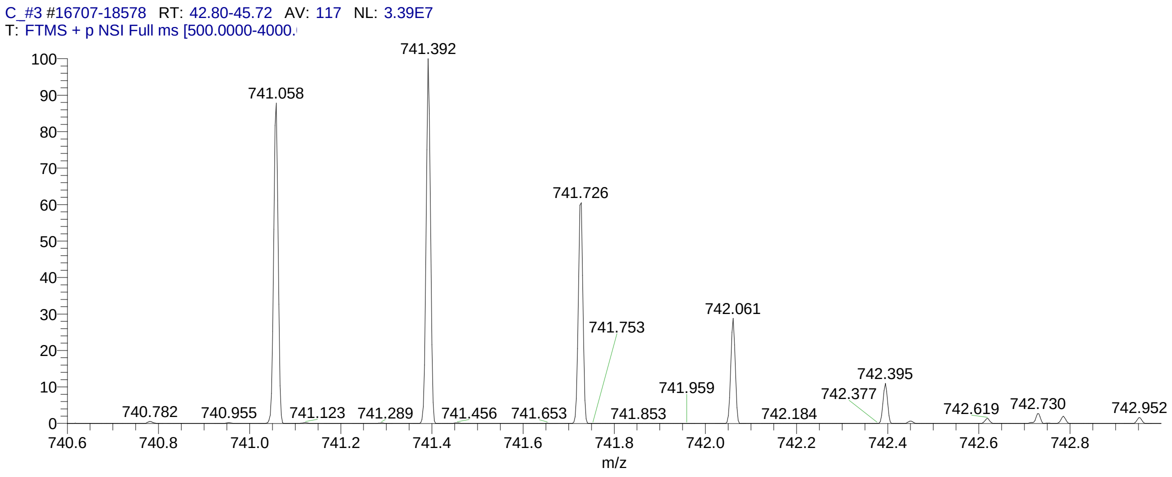

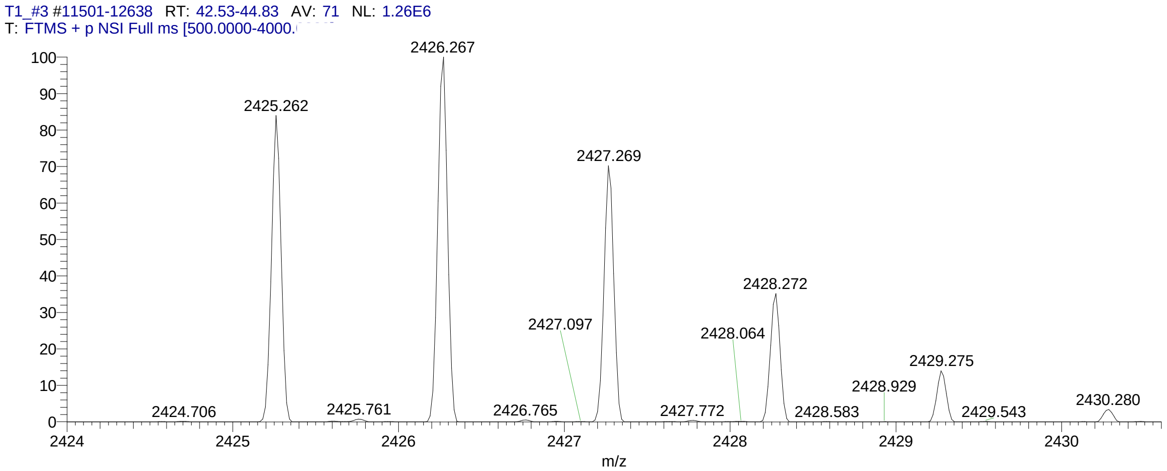

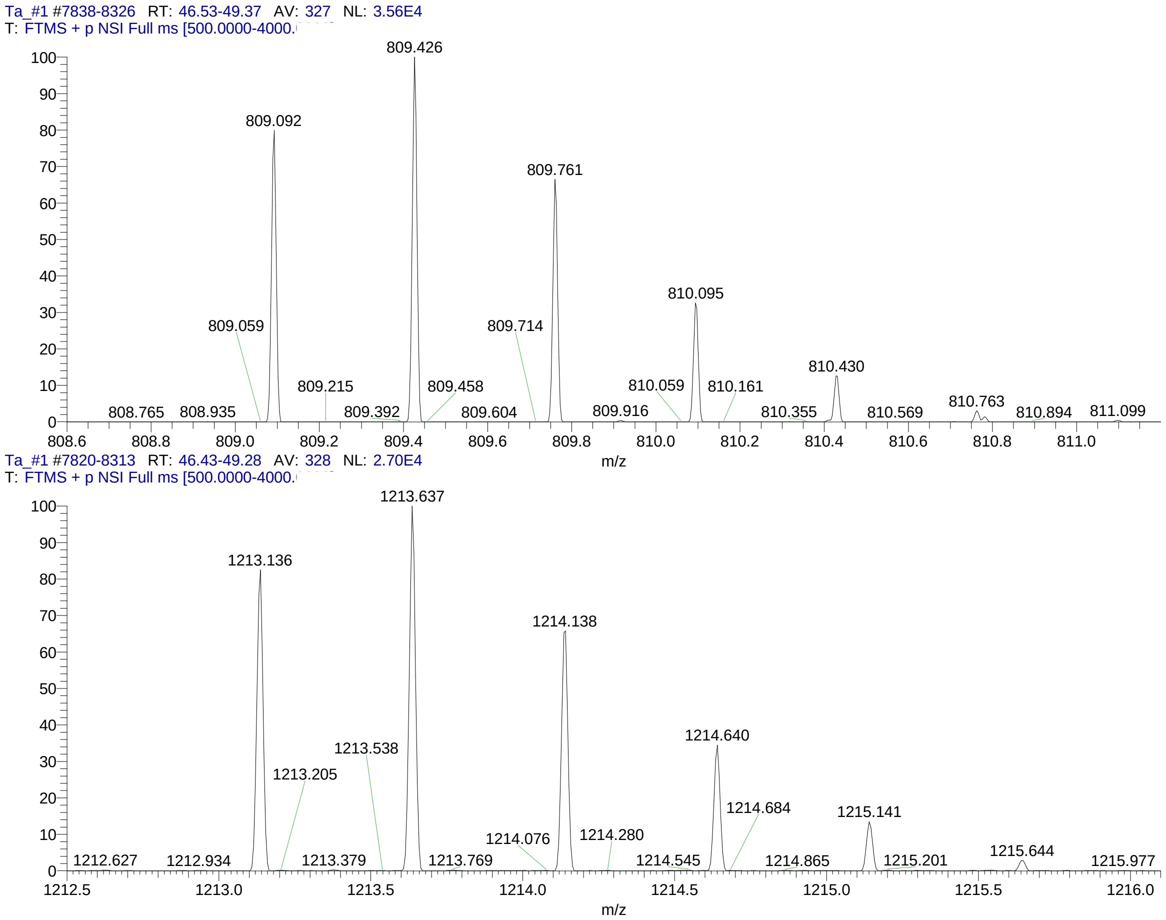

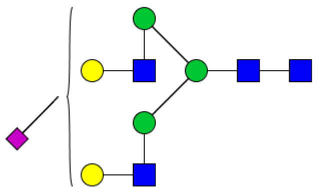

Glycan #16

HexNAc(4)Hex(5)NeuAc(1)

Permethylated, reduced

Theoretical m/z 2425.2583 (z=1)

Theoretical m/z 1213.1328 (z=2)

Theoretical m/z 809.0909 (z=3)

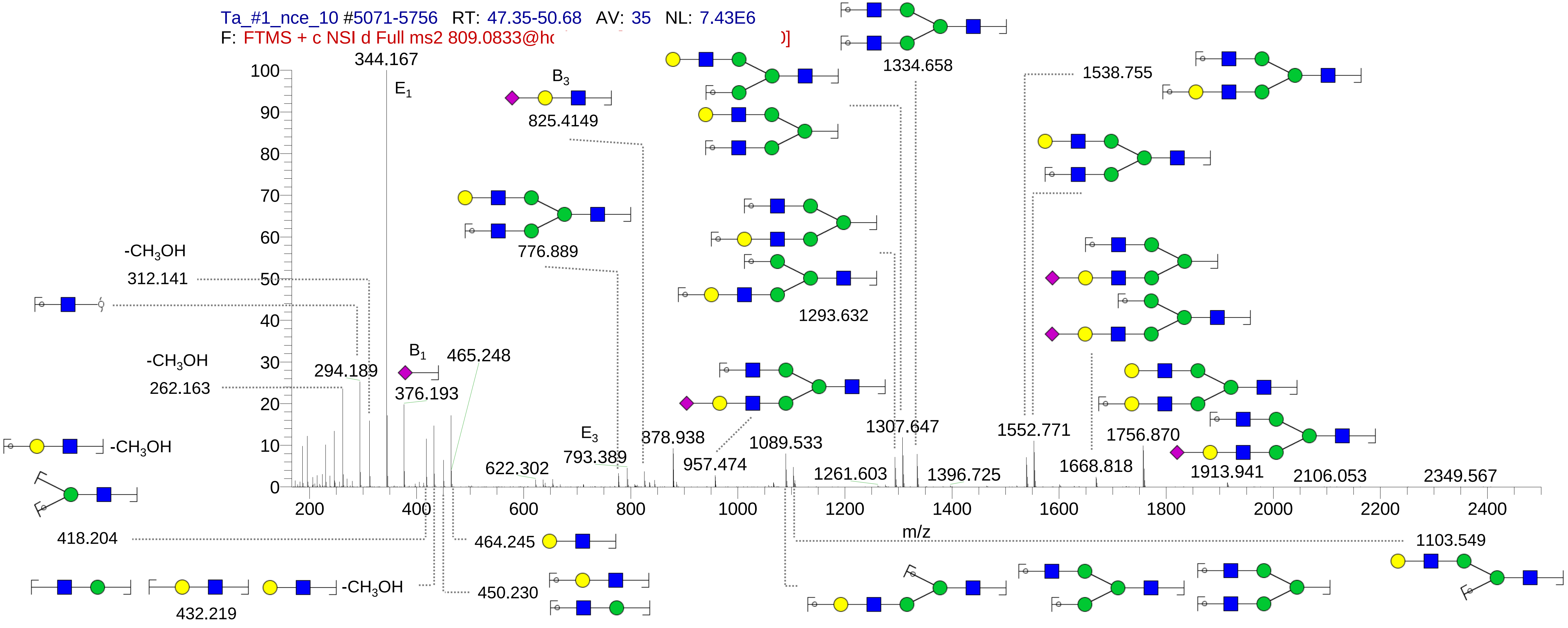

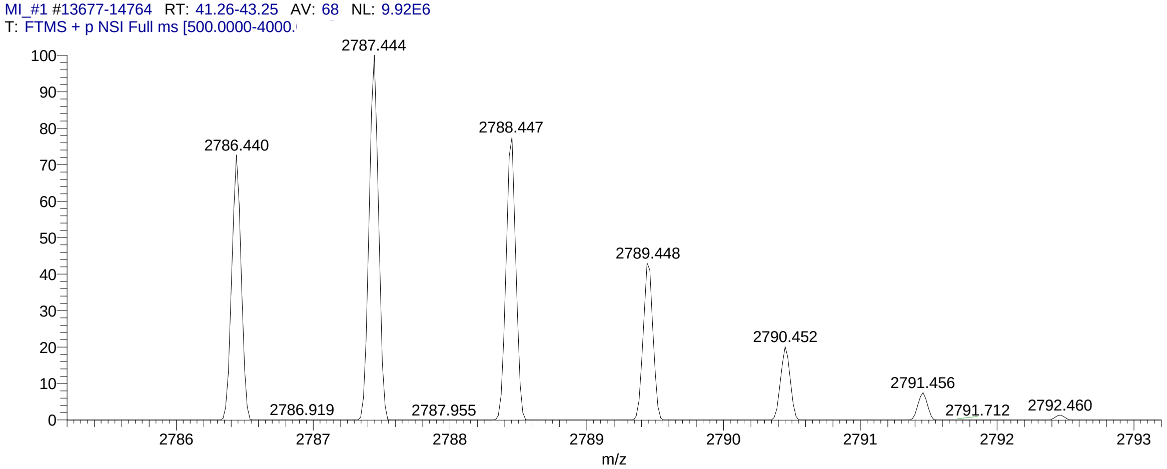

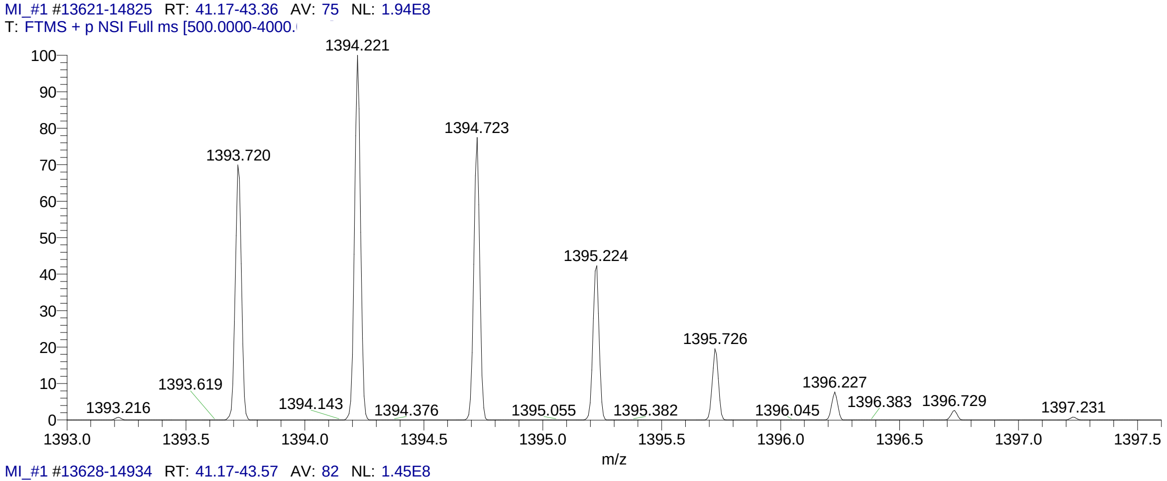

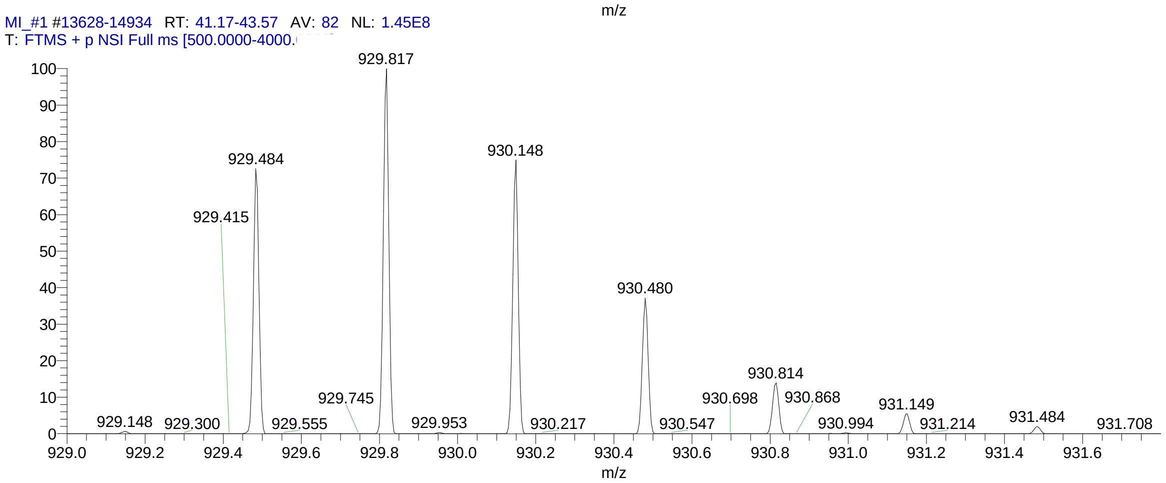

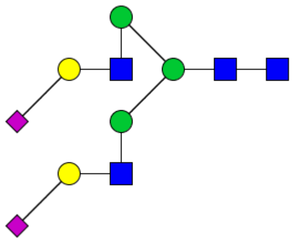

Glycan #17

HexNAc(4)Hex(5)NeuAc(2)

Permethylated, reduced

Theoretical m/z 2786.4319 (z=1)

Theoretical m/z 1393.7196 (z=2)

Theoretical m/z 929.4822 (z=3)

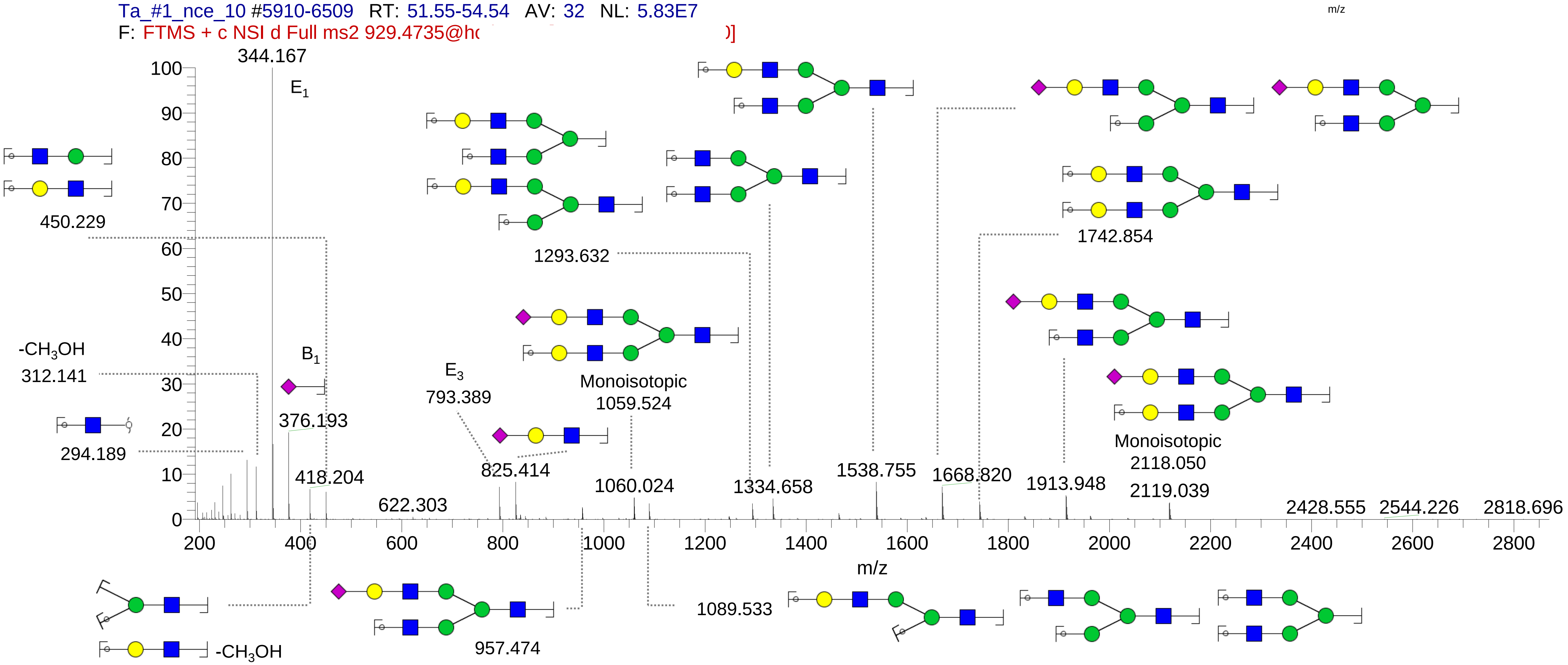

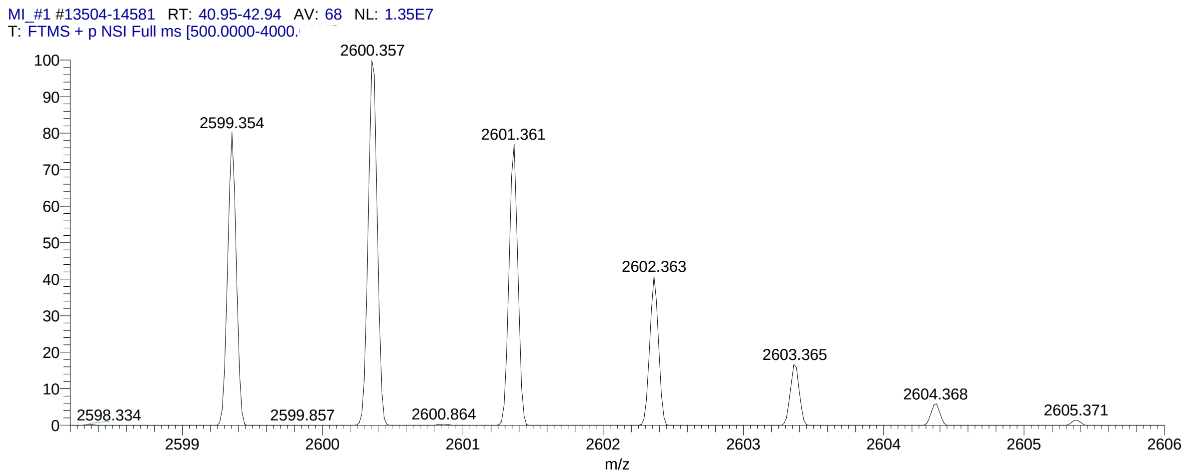

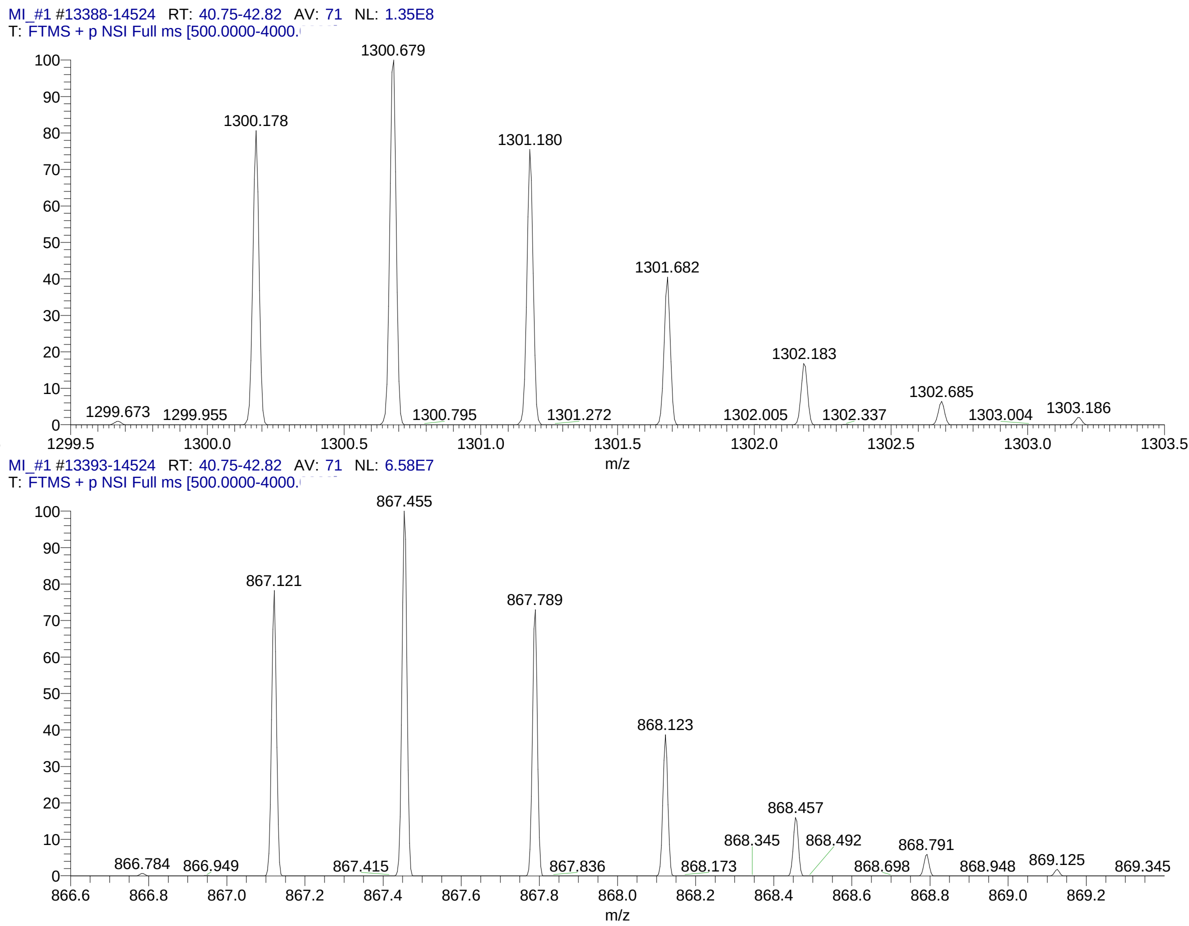

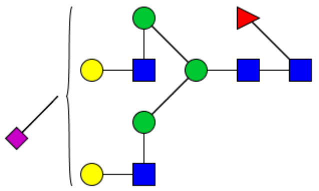

Glycan #18

HexNAc(4)Hex(5)Fuc(1)NeuAc(1)

Permethylated, reduced

Theoretical m/z 2599.3475 (z=1)

Theoretical m/z 1300.1774 (z=2)

Theoretical m/z 867.1207 (z=3)

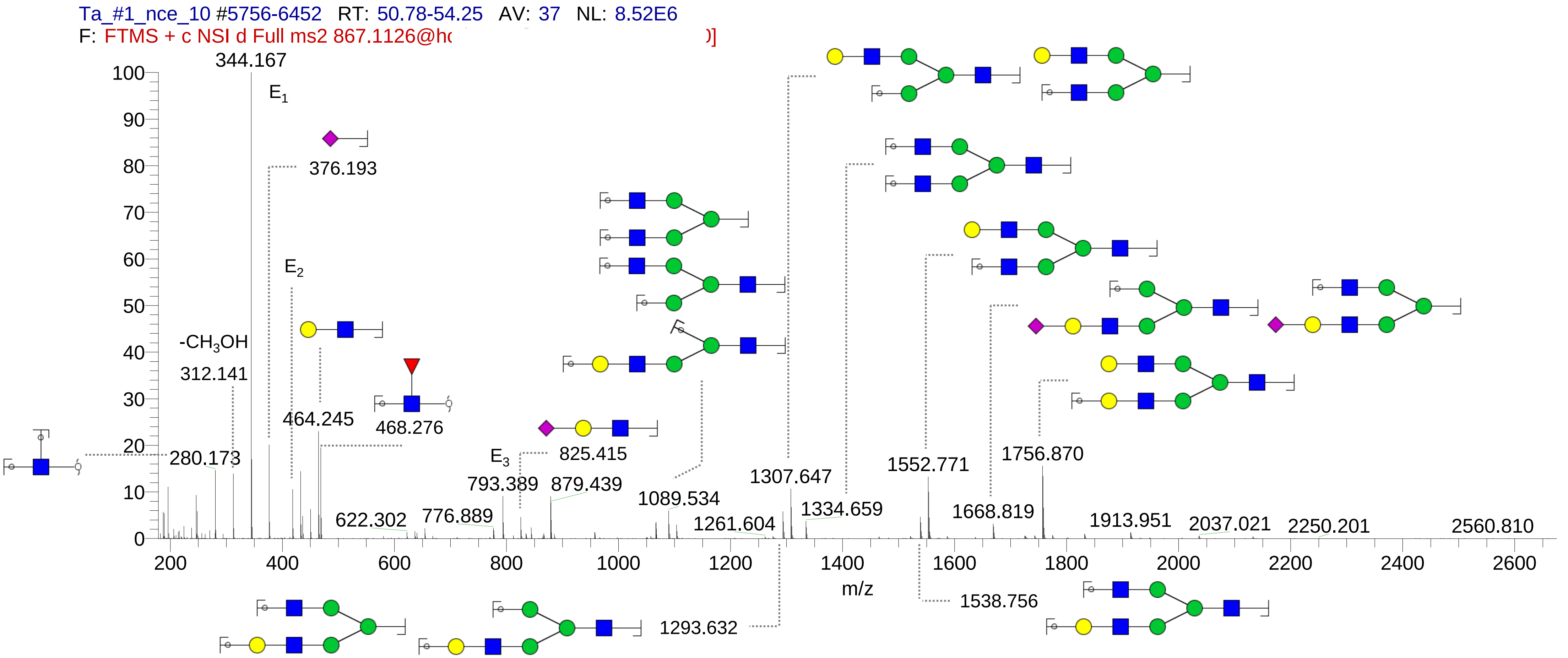

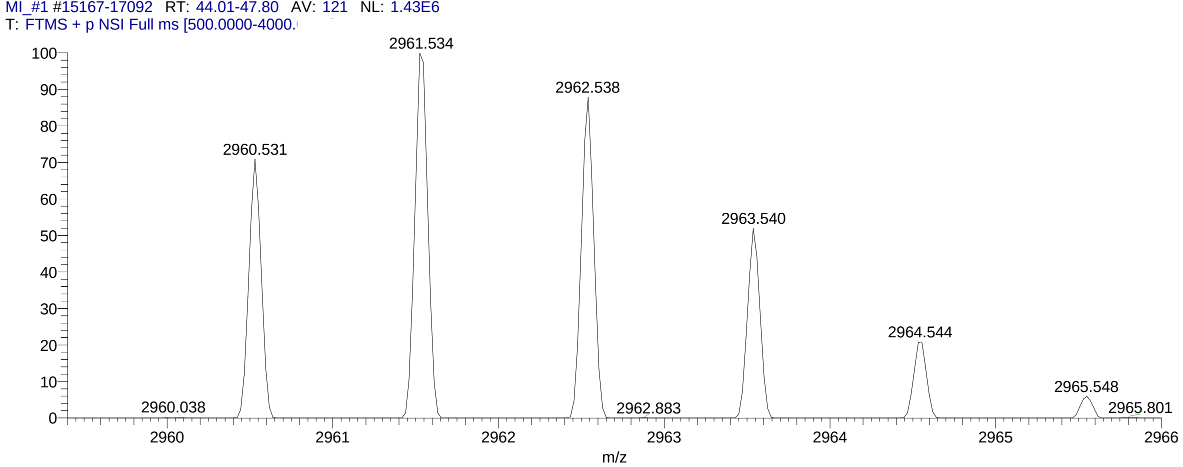

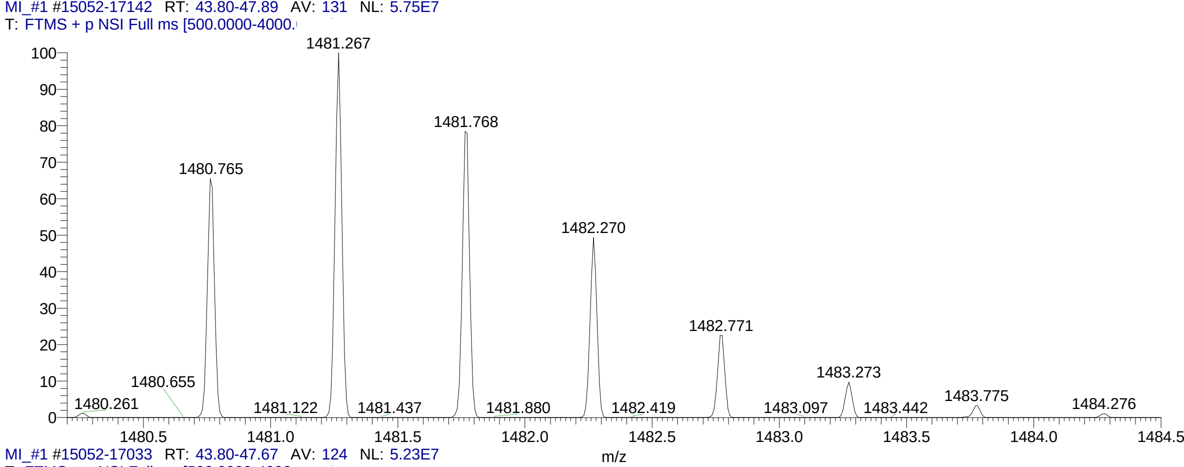

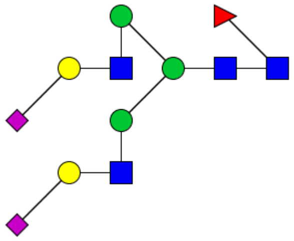

Glycan #19

HexNAc(4)Hex(5)Fuc(1)NeuAc(2)

Permethylated, reduced

Theoretical m/z 2960.5211 (z=1)

Theoretical m/z 1480.7642 (z=2)

Theoretical m/z 987.5119 (z=3)

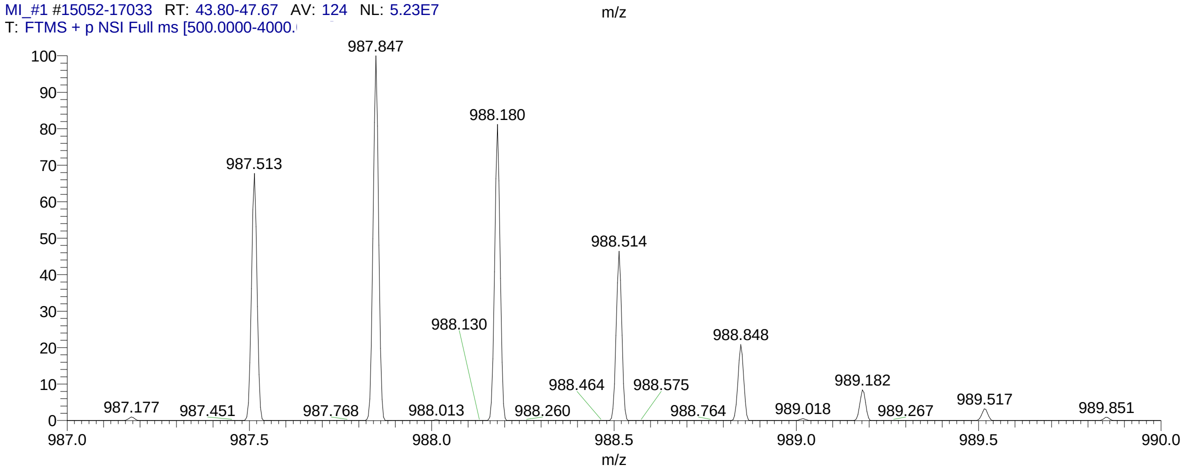

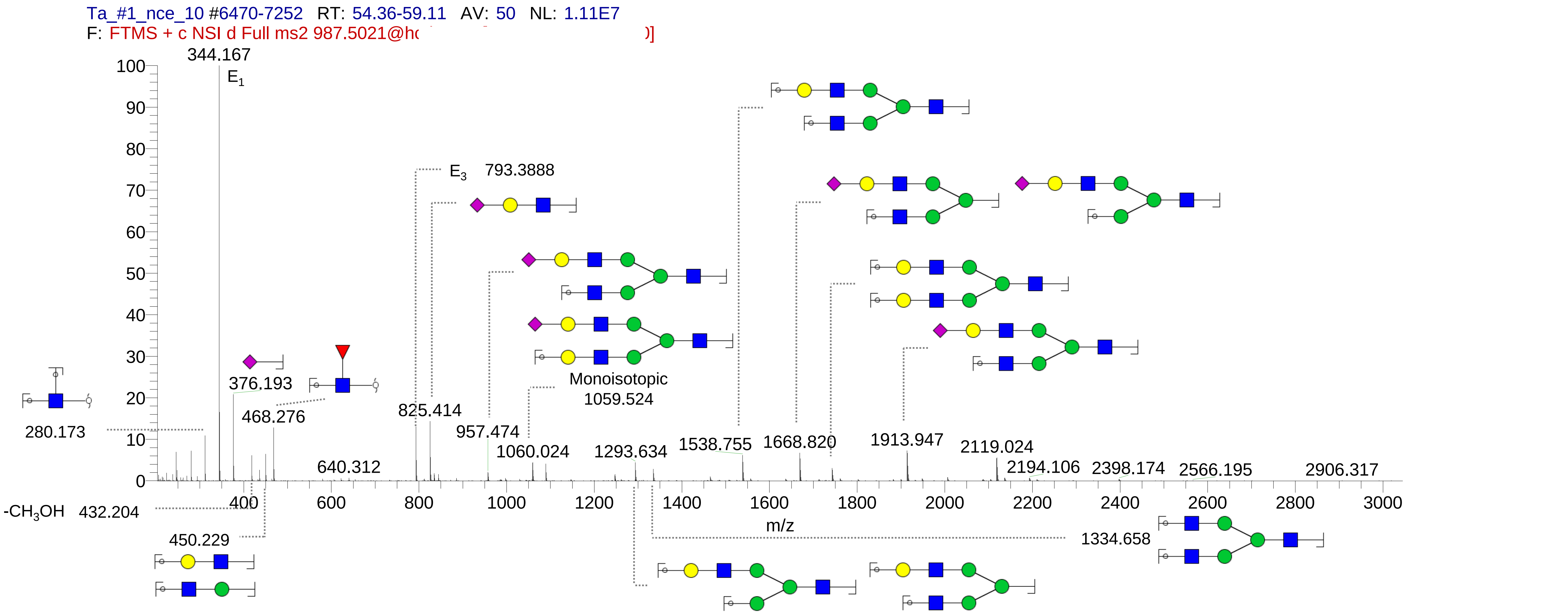

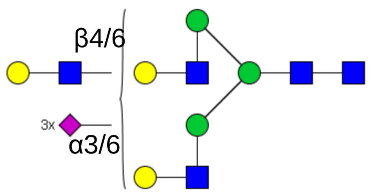

Glycan #20

HexNAc(5)Hex(6)NeuAc(3)

Permethylated, reduced

Theoretical m/z 1798.9195 (z=2)

Theoretical m/z 1199.6154 (z=3)

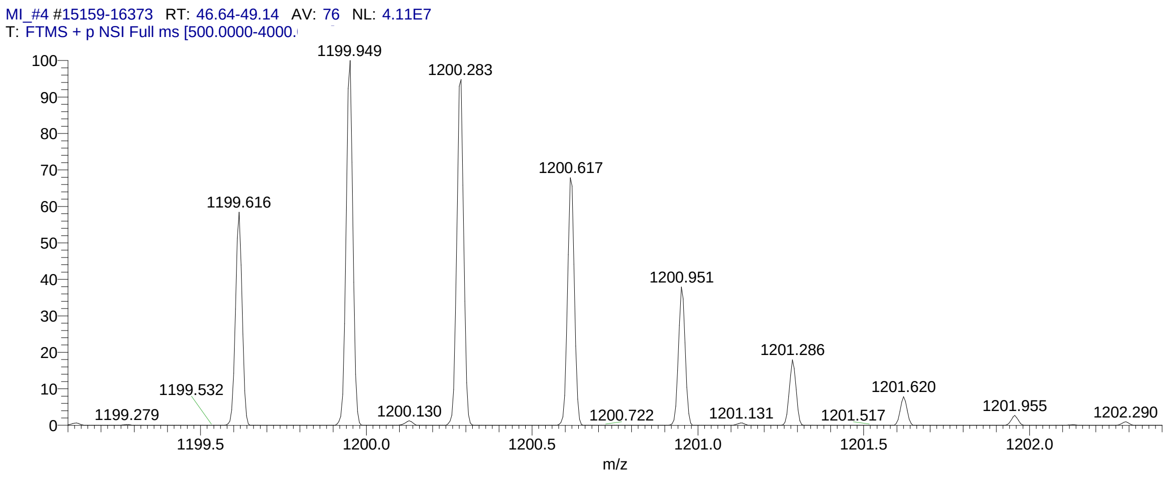

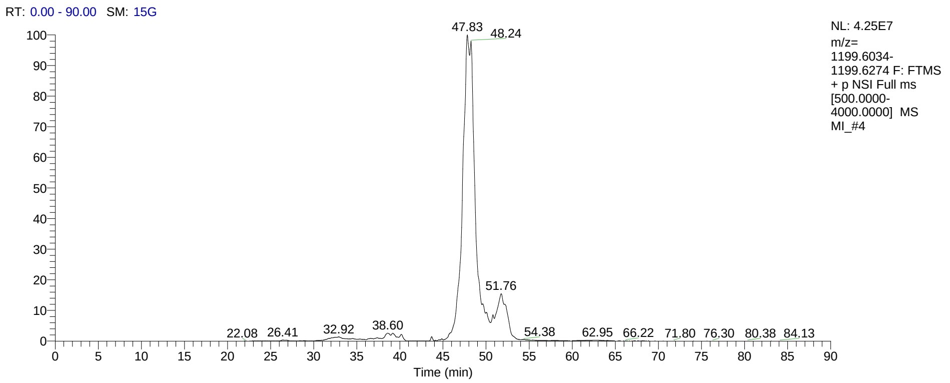

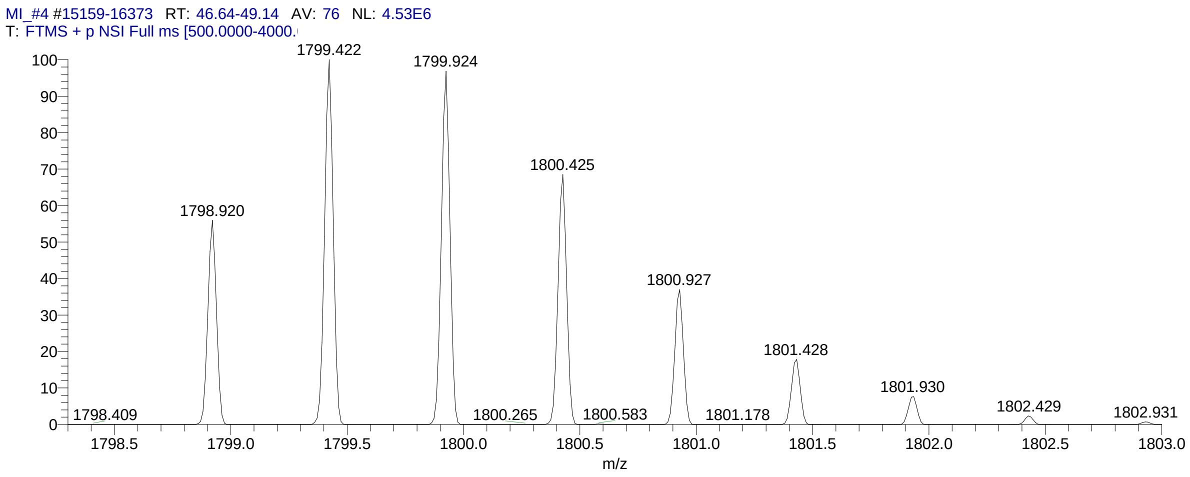

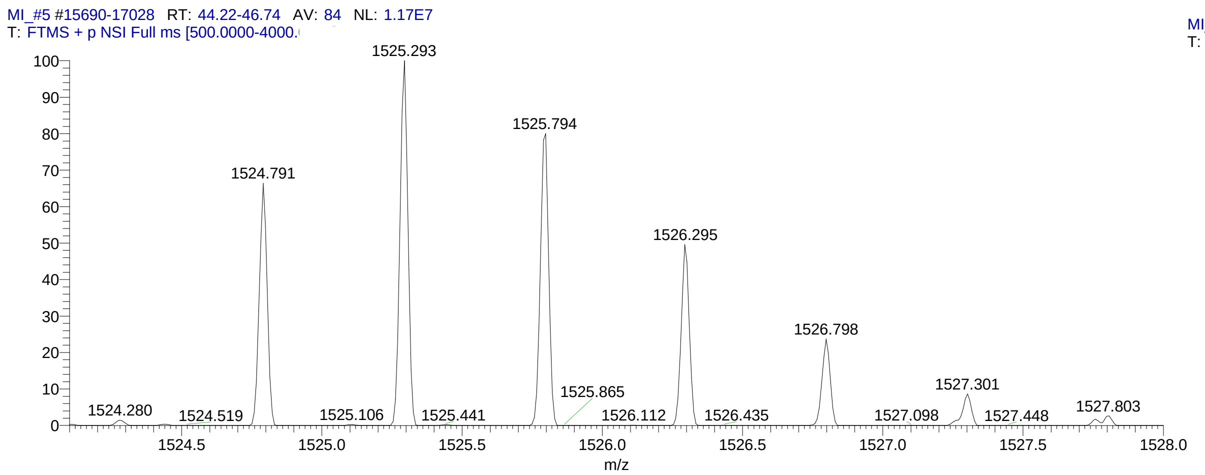

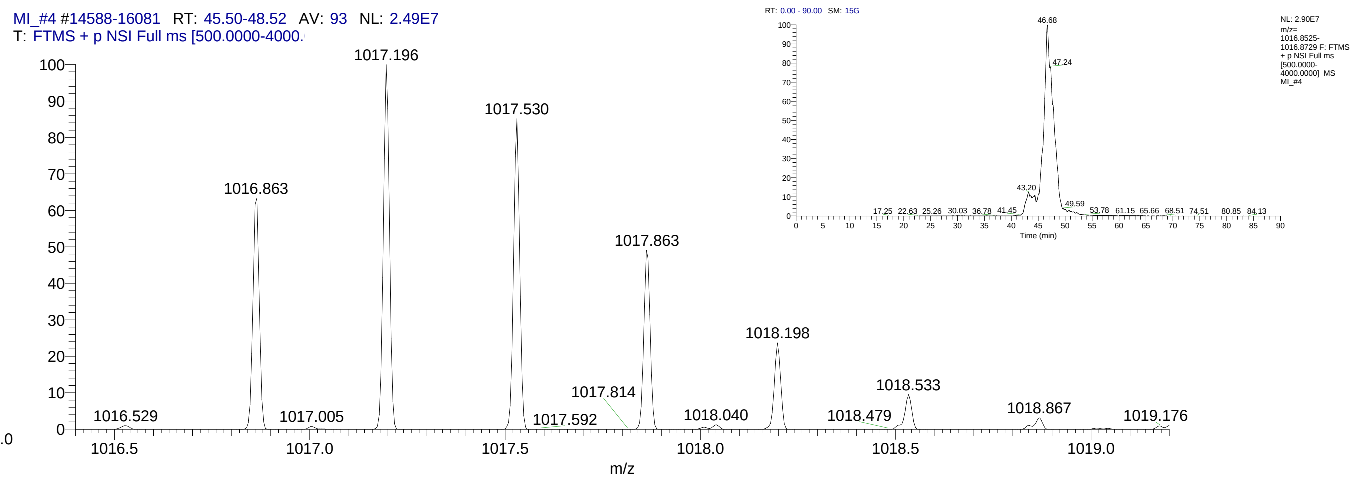

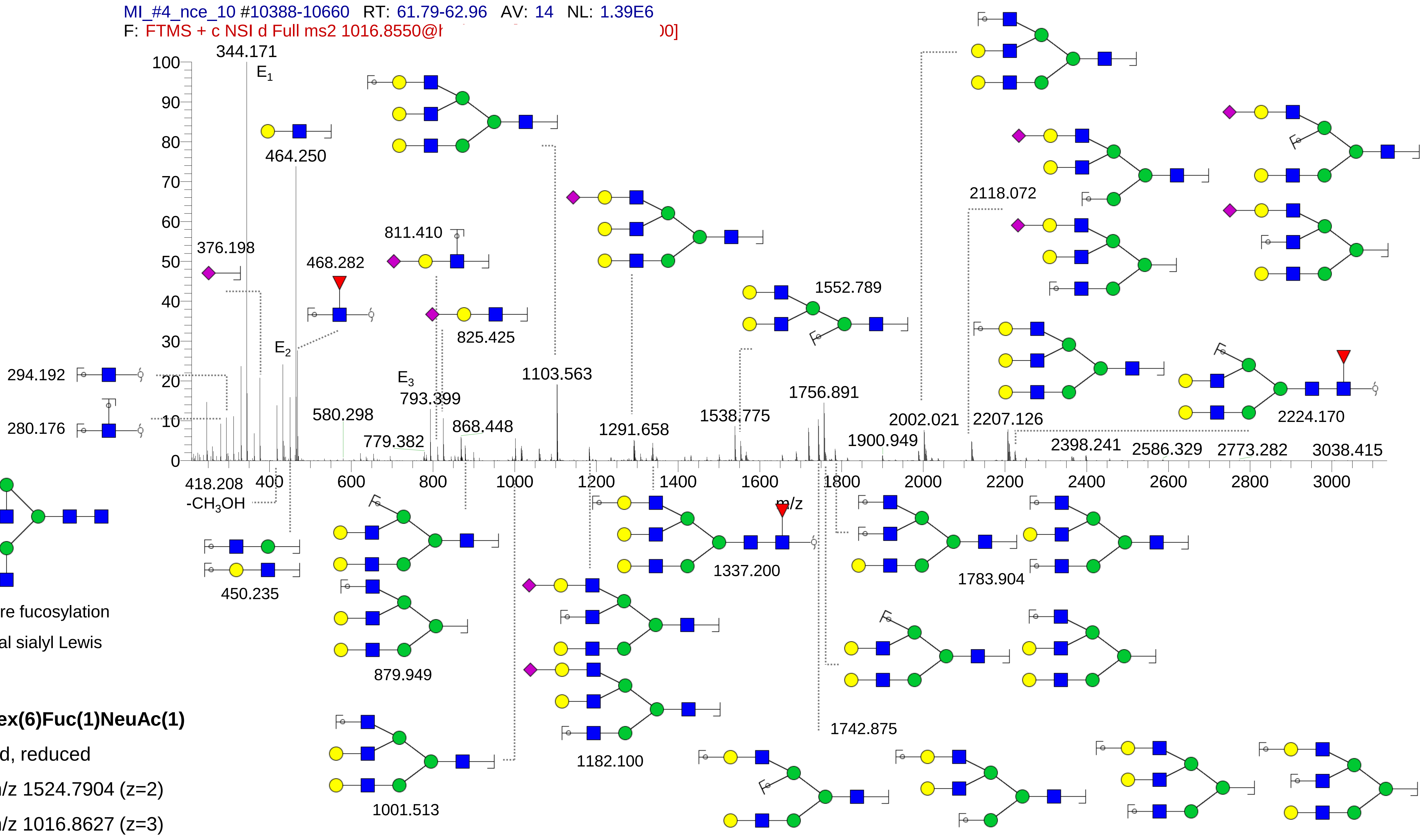

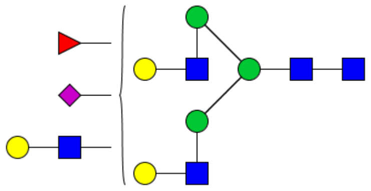

Presence of core fucosylation

but also terminal sialyl Lewis

Glycan #21

HexNAc(5)Hex(6)Fuc(1)NeuAc(1)

Permethylated, reduced

Theoretical m/z 1524.7904 (z=2)

Theoretical m/z 1016.8627 (z=3)

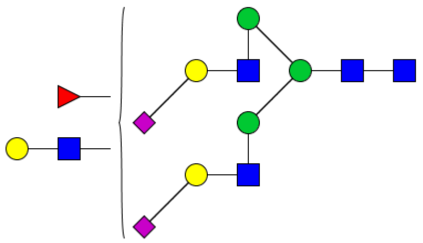

Glycan #22

HexNAc(5)Hex(6)Fuc(1)NeuAc(2)

Permethylated, reduced

Theoretical m/z 1705.3773 (z=2)

Theoretical m/z 1137.2539 (z=3)

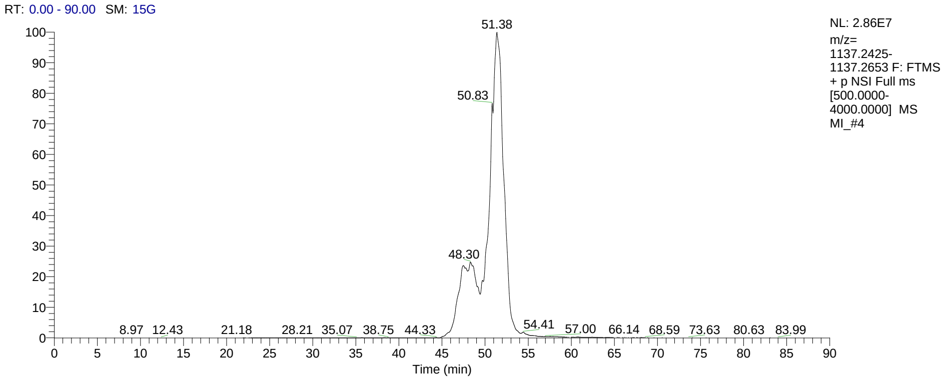

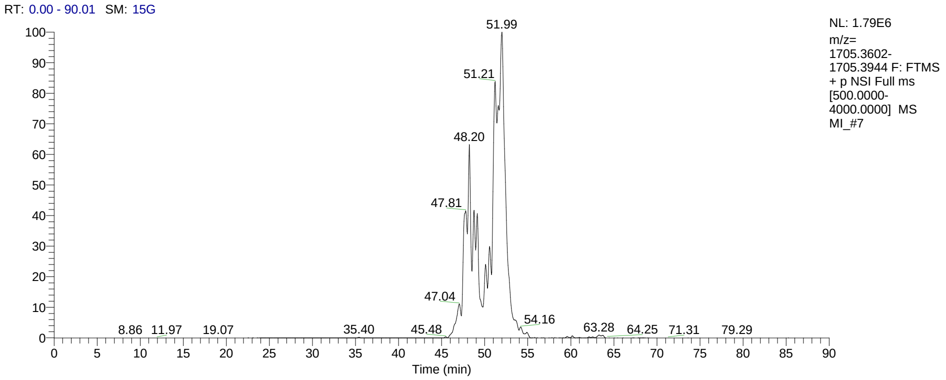

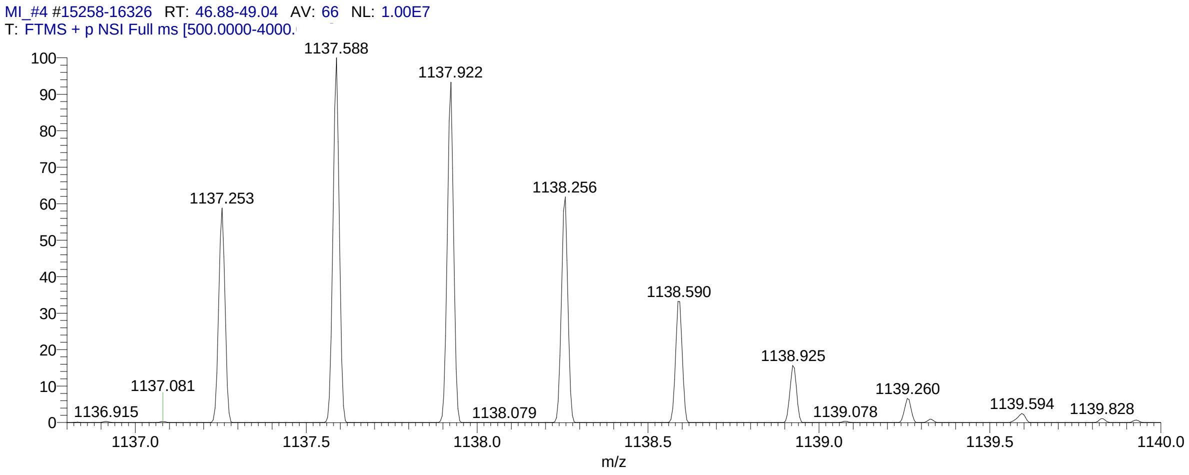

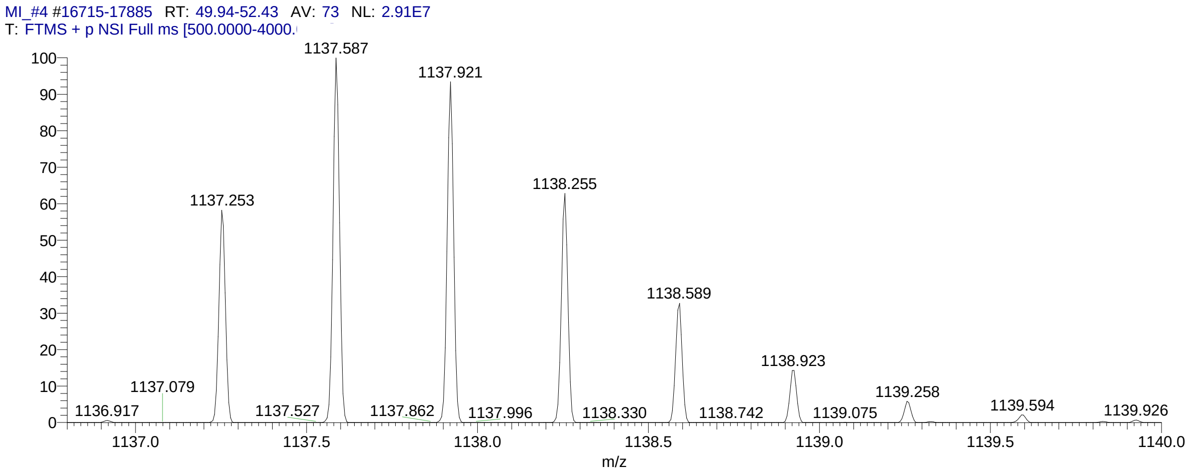

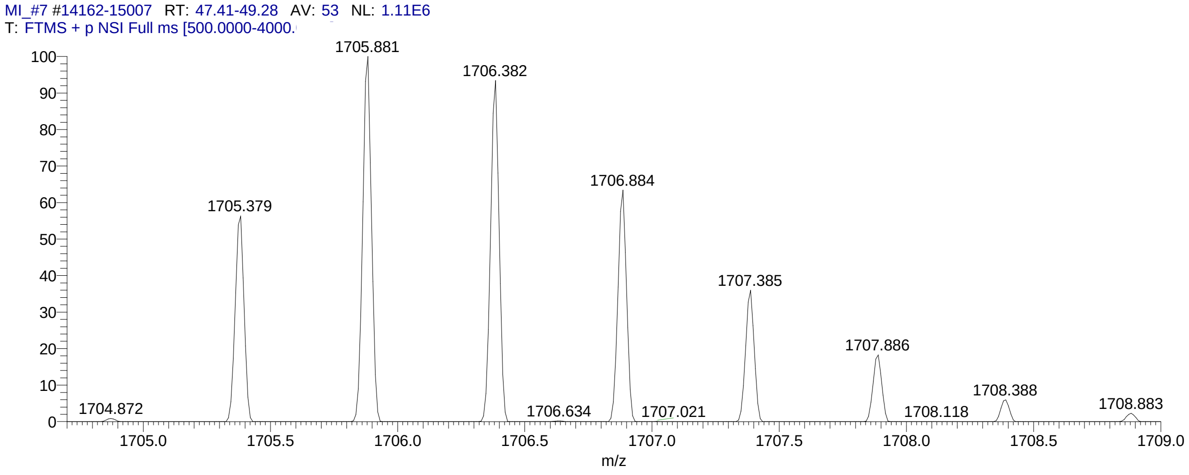

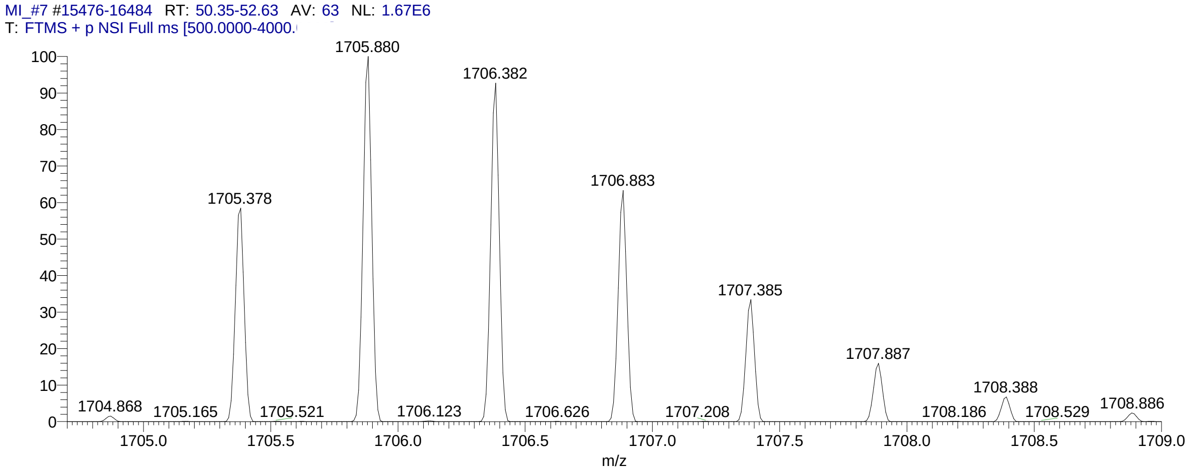

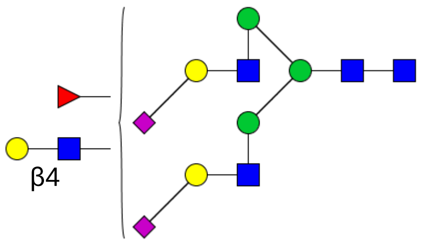

Glycan #22

HexNAc(5)Hex(6)Fuc(1)NeuAc(2)

Permethylated, reduced

Theoretical m/z 1705.3773 (z=2)

Theoretical m/z 1137.2539 (z=3)

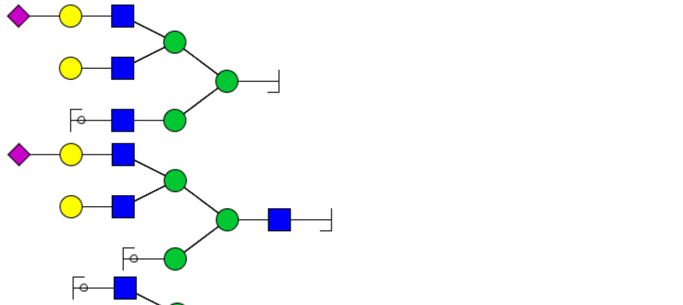

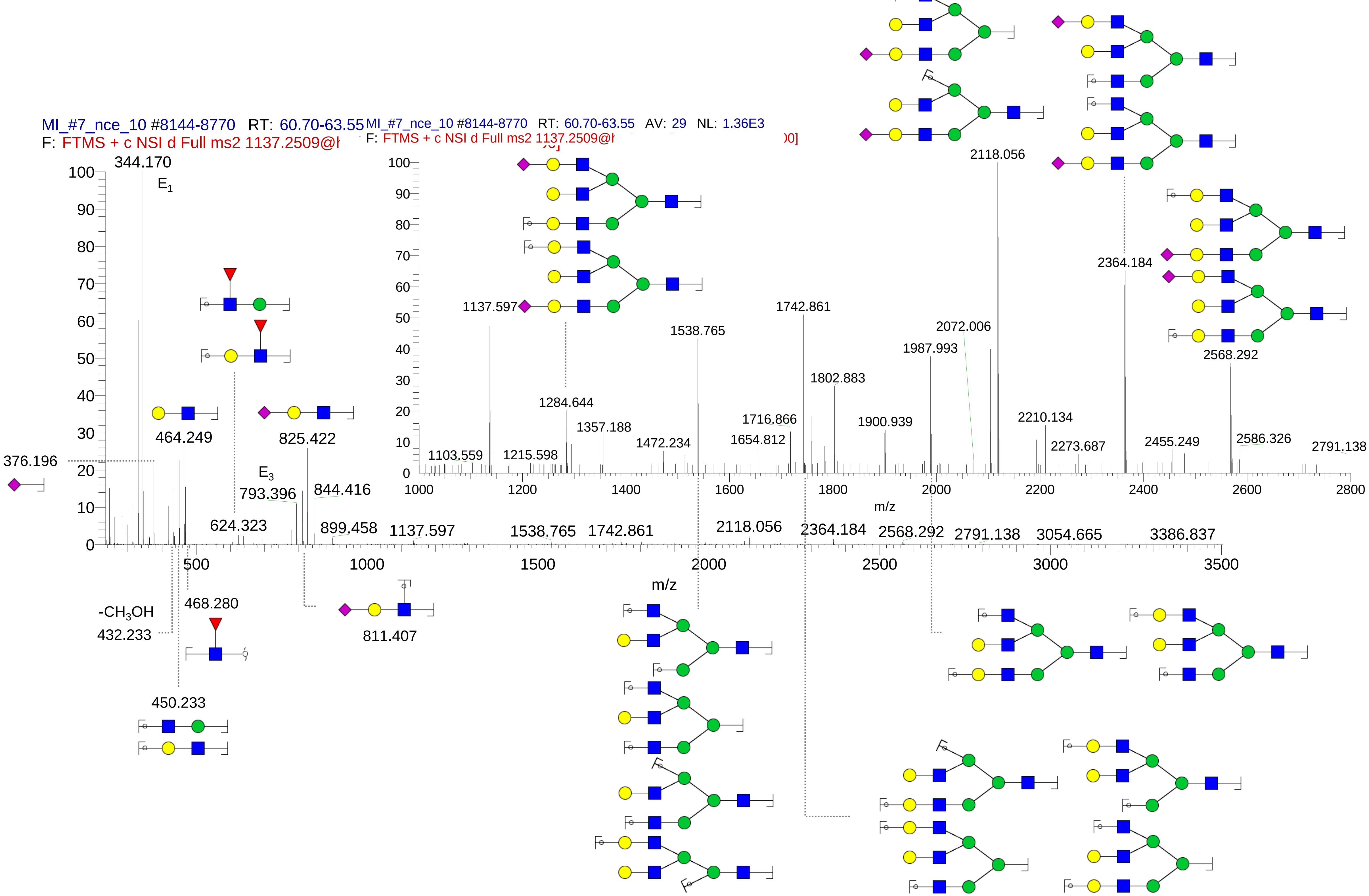

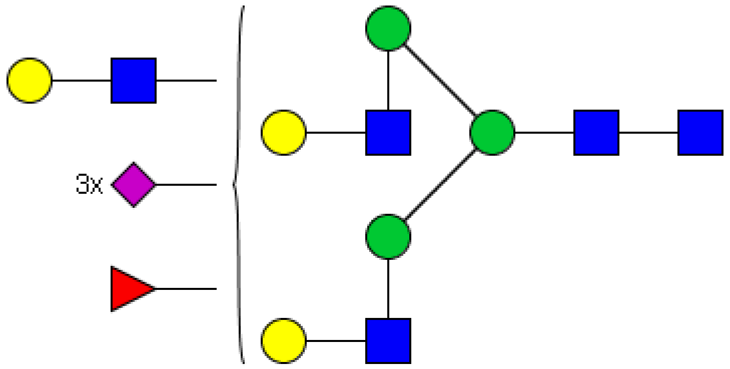

Glycan #23

HexNAc(5)Hex(6)Fuc(1)NeuAc(3)

Permethylated, reduced

Theoretical m/z 1257.6452 (z=3)

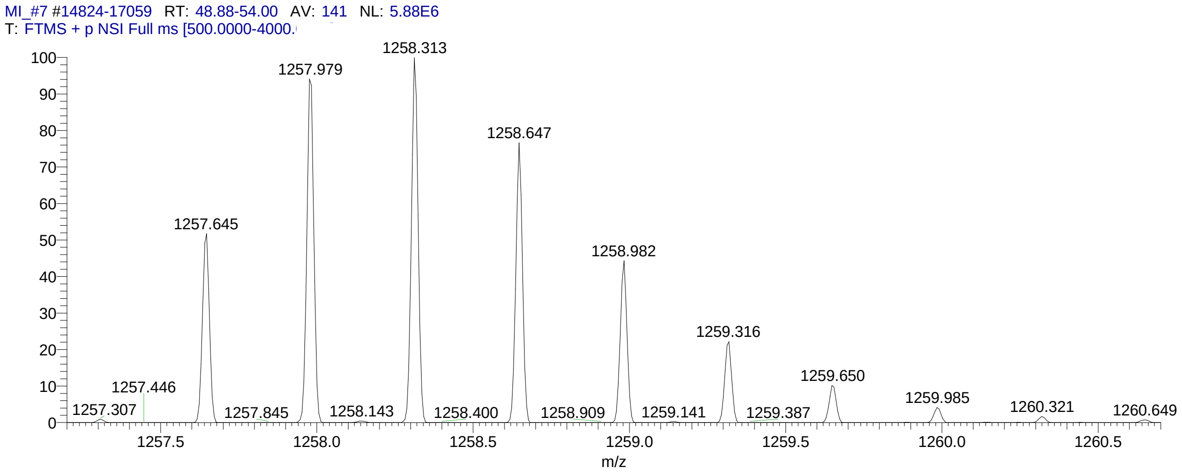

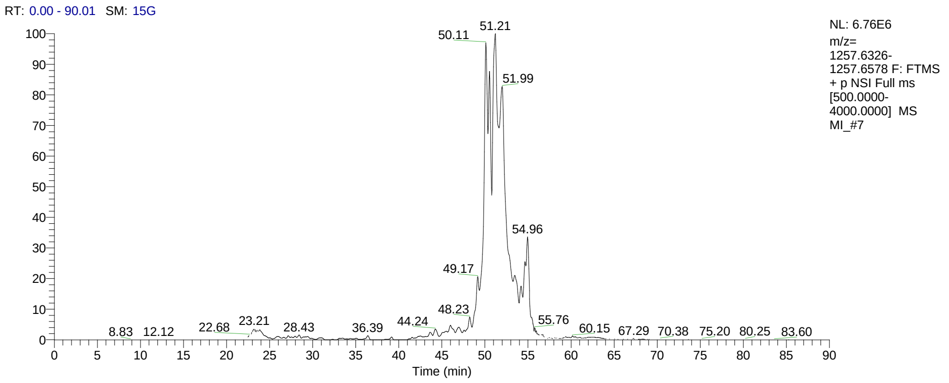

Myeloperoxidase

Sample: MI_1

m/z 1154.998

Glycan composition:

HexNAc(2)Hex(2)Fuc(1)

Glycosite Asn323

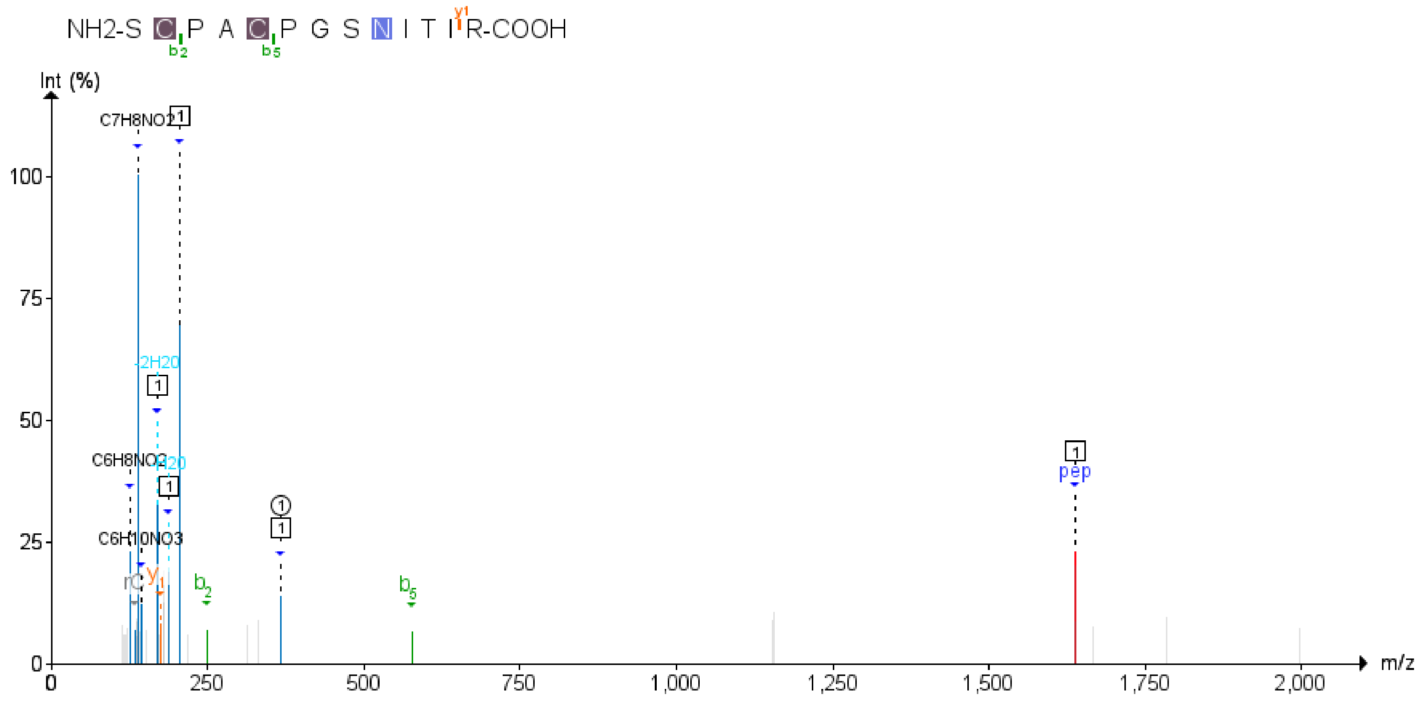

Myeloperoxidase

Sample: MI_2

m/z 1236.025

Glycan composition:

HexNAc(2)Hex(3)Fuc(1)

Glycosite Asn323

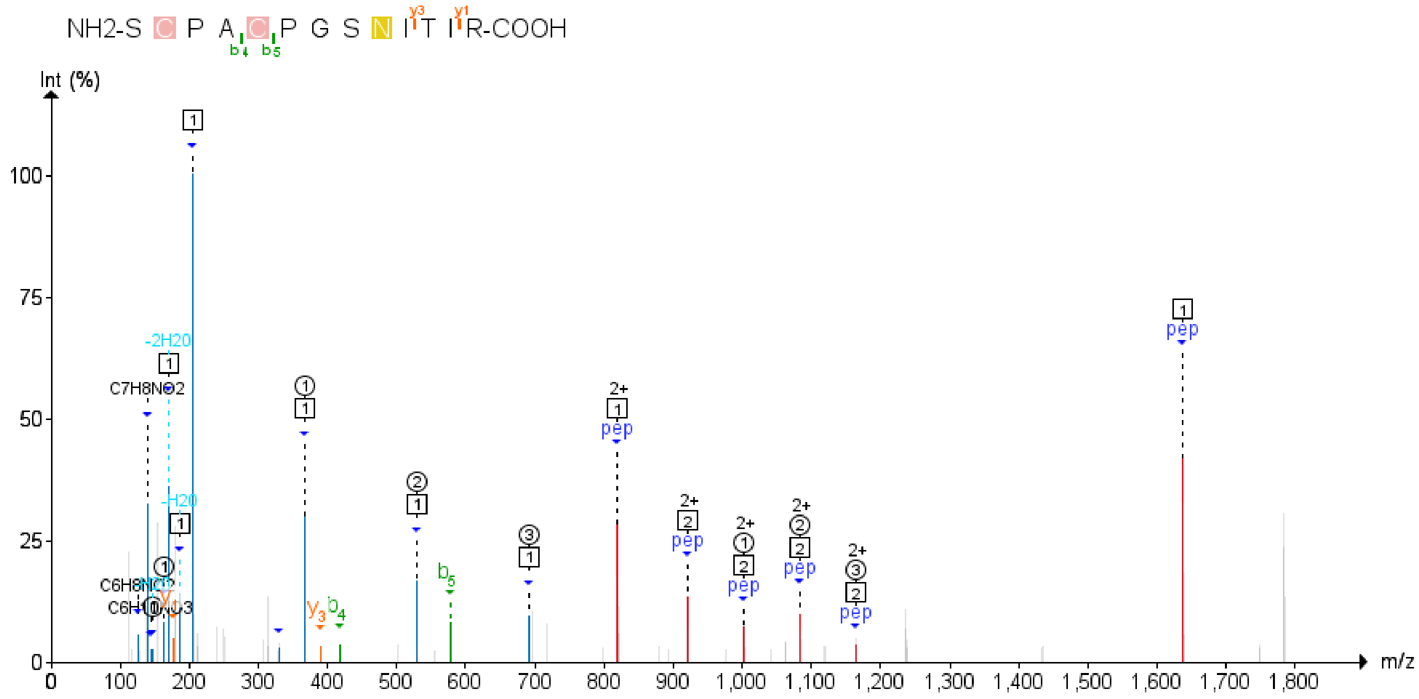

Myeloperoxidase

Sample: T1_2

m/z 998.443

Glycan composition

HexNAc(3)Hex(5)

Glycosite Asn355

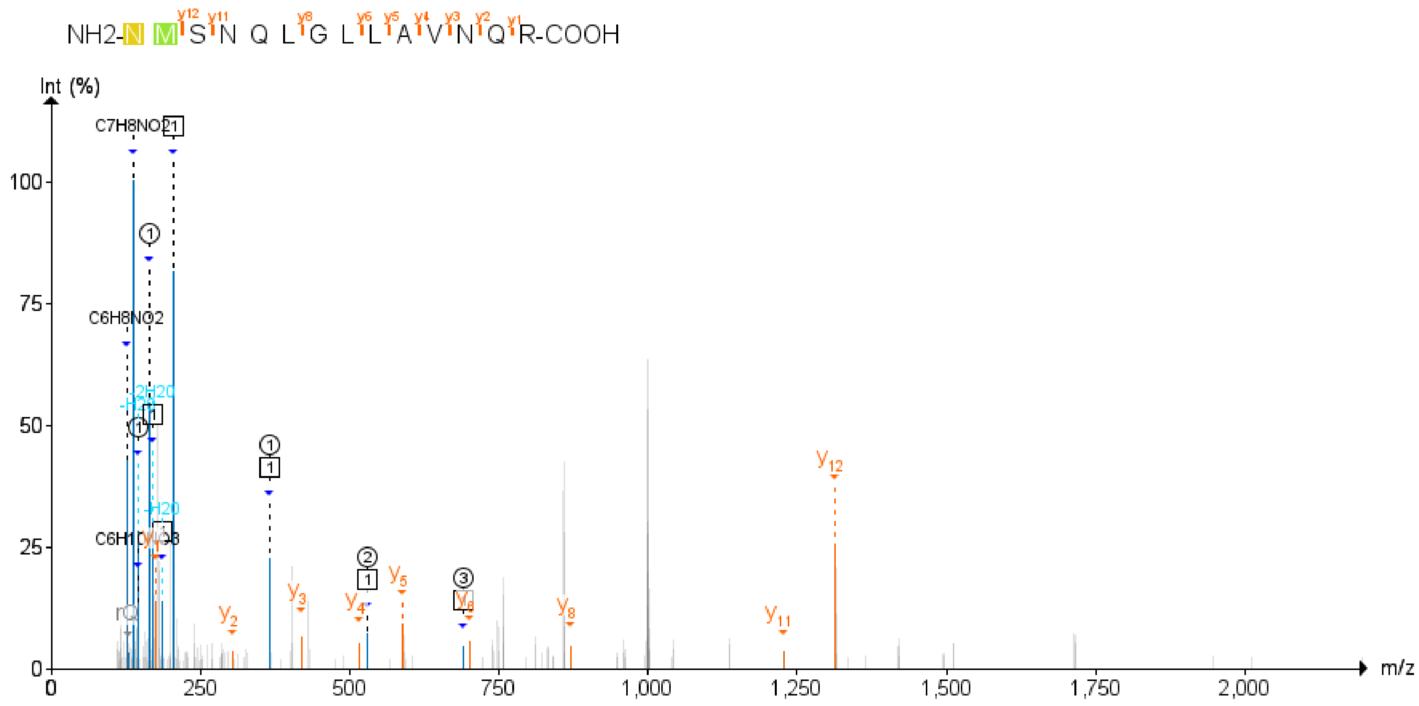

Myeloperoxidase

Sample: T1_2

m/z 1006.129

Glycan composition:

HexNAc(2)Hex(3)

Glycosite Asn391

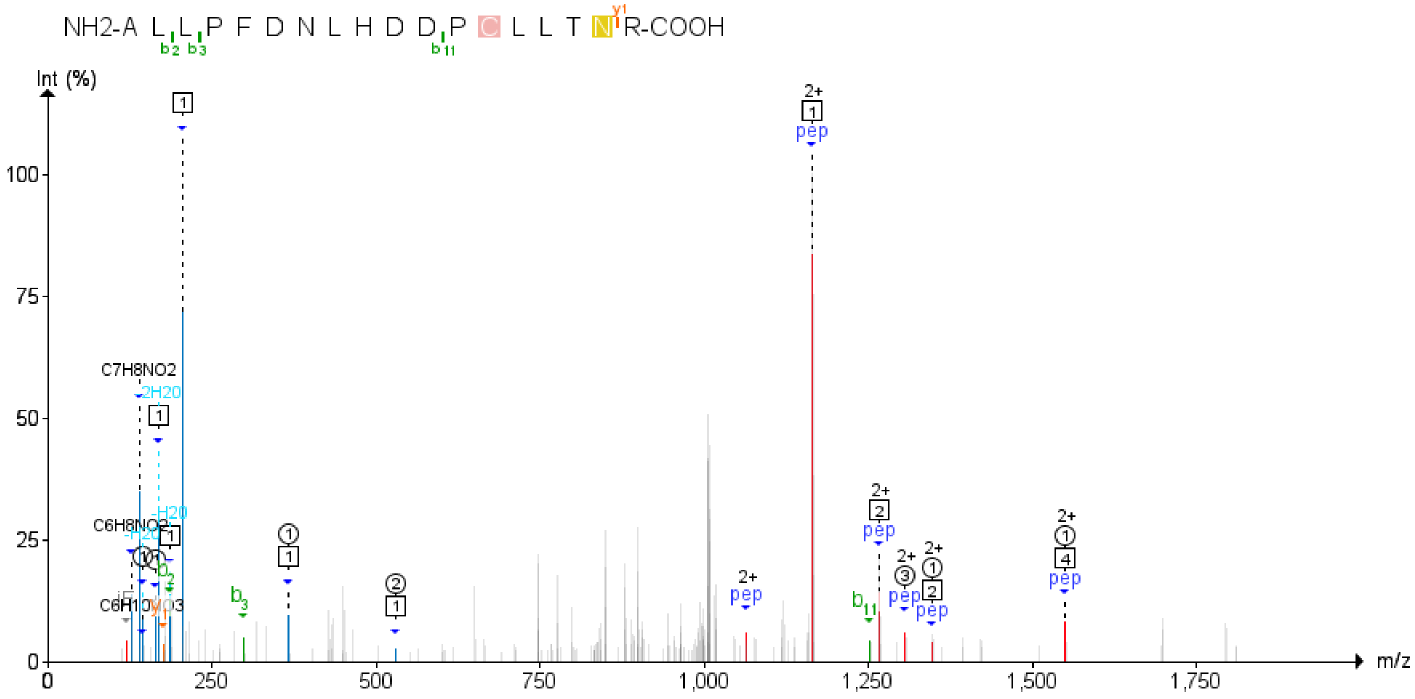

Myeloperoxidase

Sample: T1_2

m/z 1060.146

Glycan composition:

HexNAc(2)Hex(4)

Glycosite Asn391

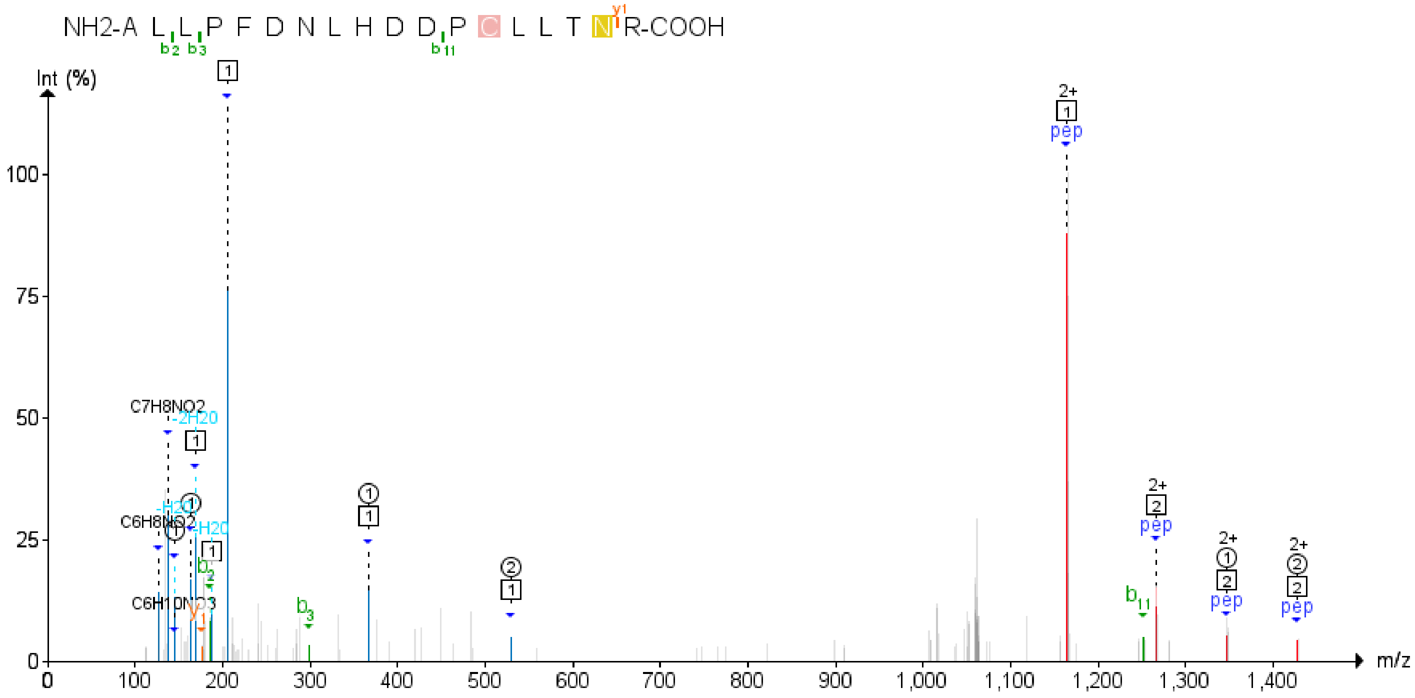

Myeloperoxidase

Sample: T1_2

m/z 1114.164

Glycan composition:

HexNAc(2)Hex(5)

Glycosite Asn391

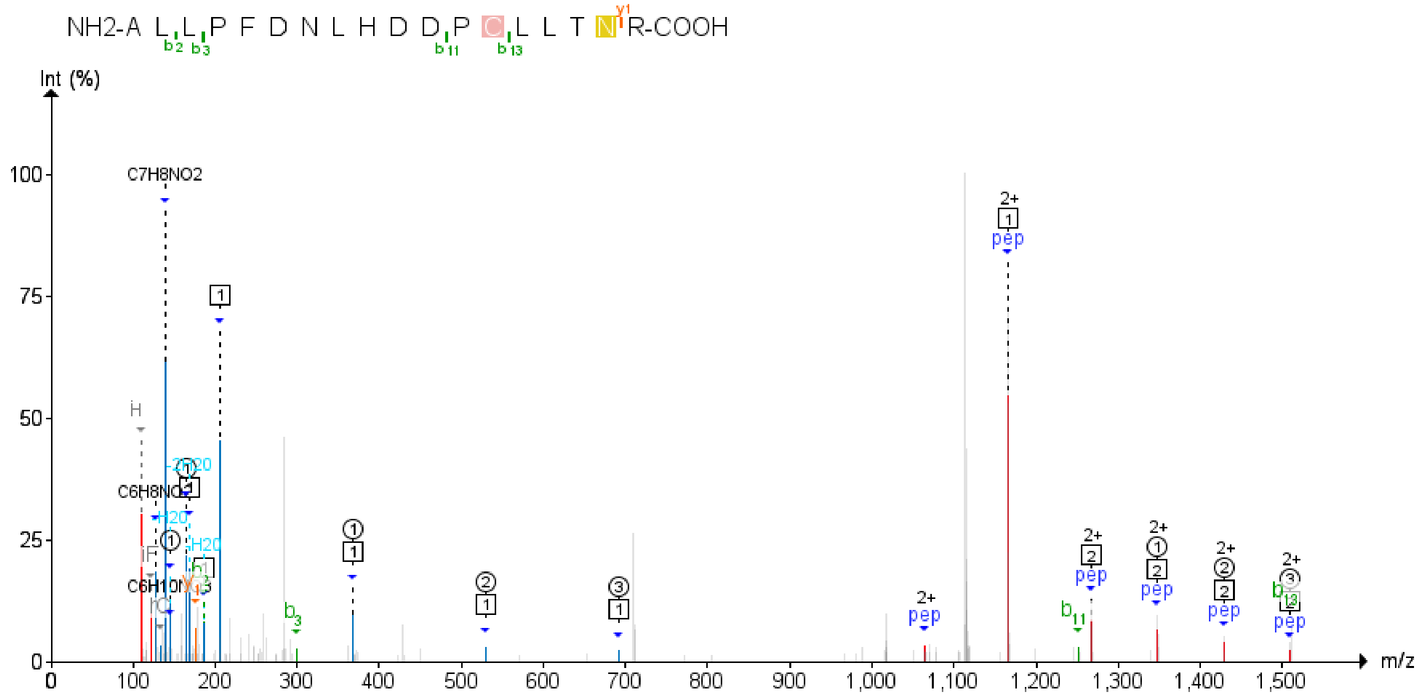

Myeloperoxidase

Sample: T1_2

m/z 1168.181

Glycan composition:

HexNAc(2)Hex(6)

Glycosite Asn391

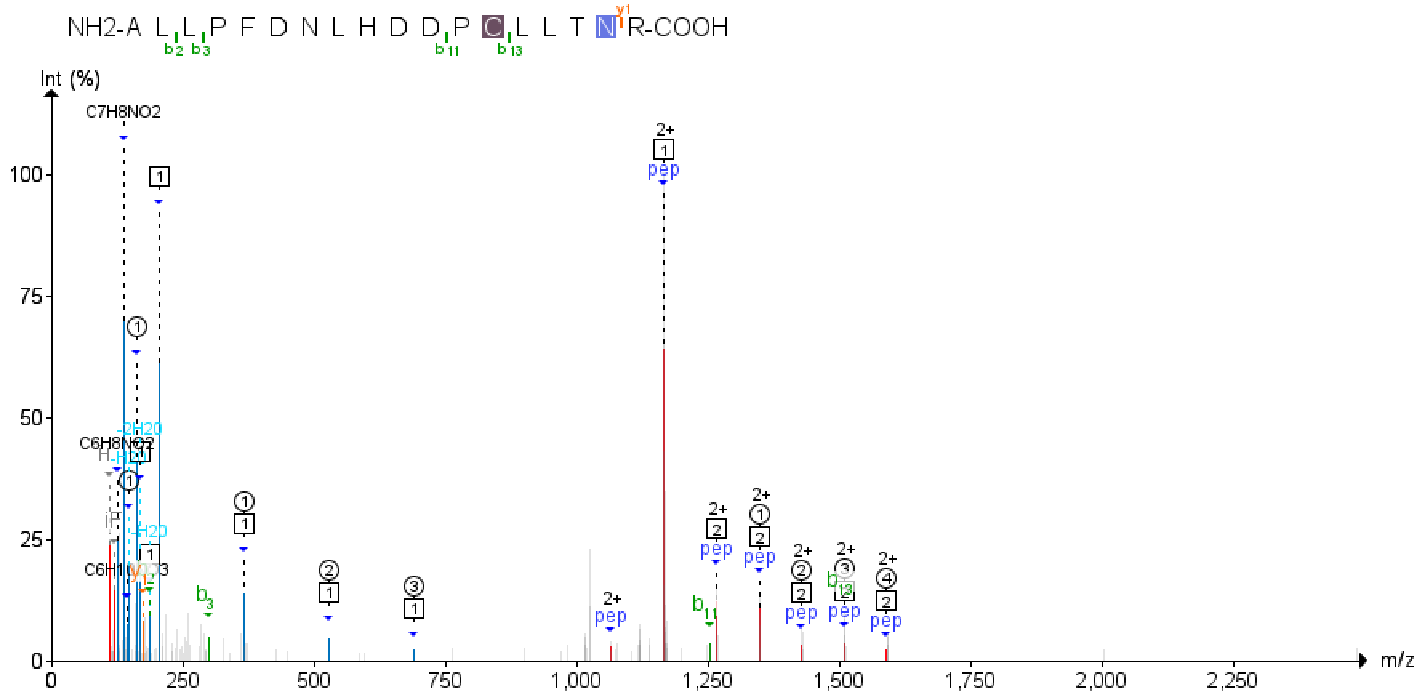

Myeloperoxidase

Sample: T1_2

m/z 1045.923

Glycan composition

HexNAc(2)Hex(3)Fuc(1)

Glycosite Asn483

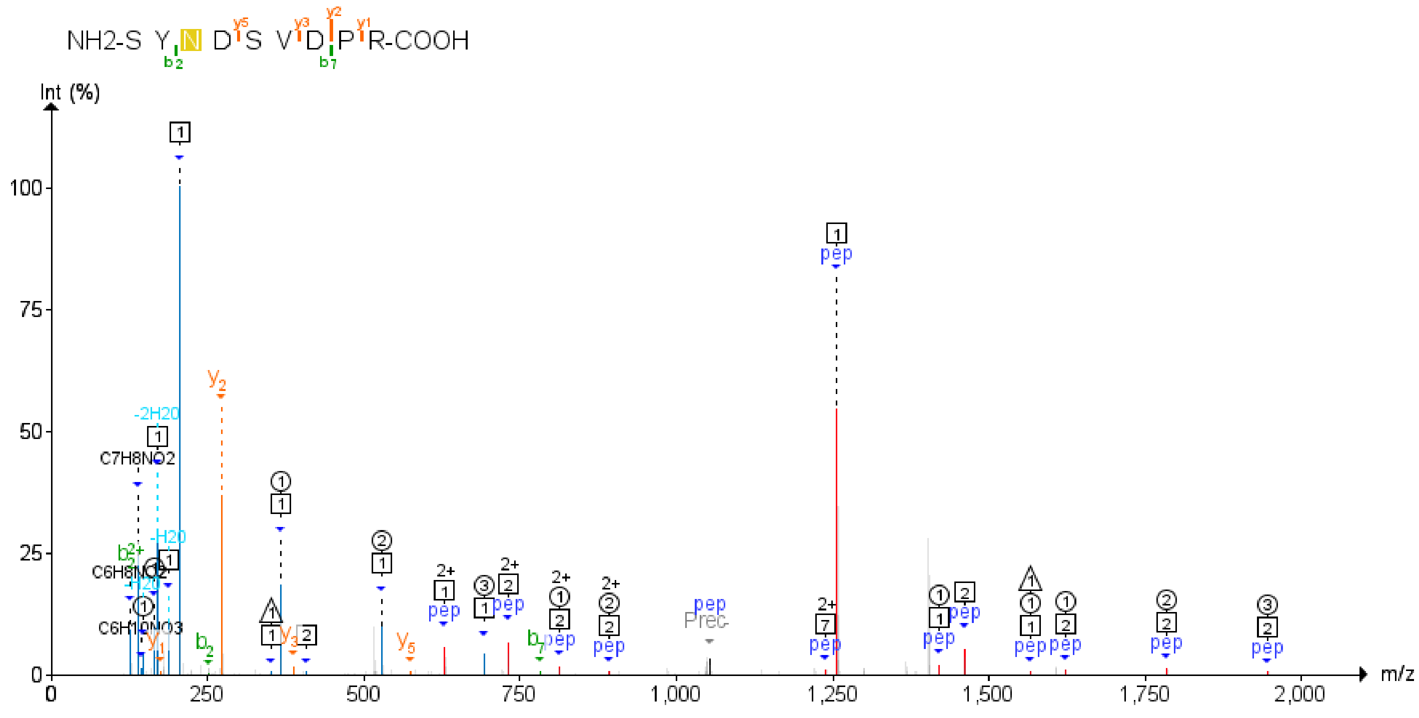

Myeloperoxidase

Sample: MI_2

m/z 1147.463

Glycan composition:

HexNAc(3)Hex(3)Fuc(1)

Glycosite Asn483

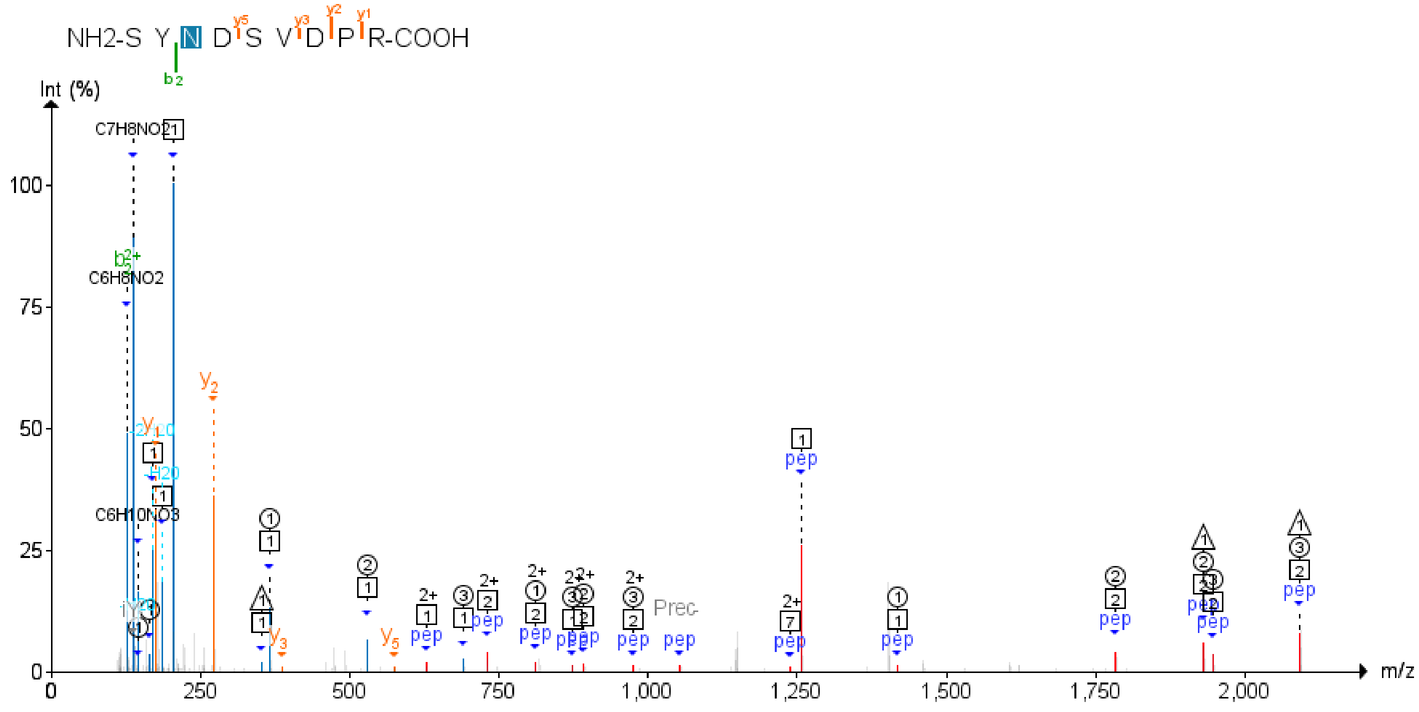

Myeloperoxidase

Sample: T1_1

m/z **575.29**

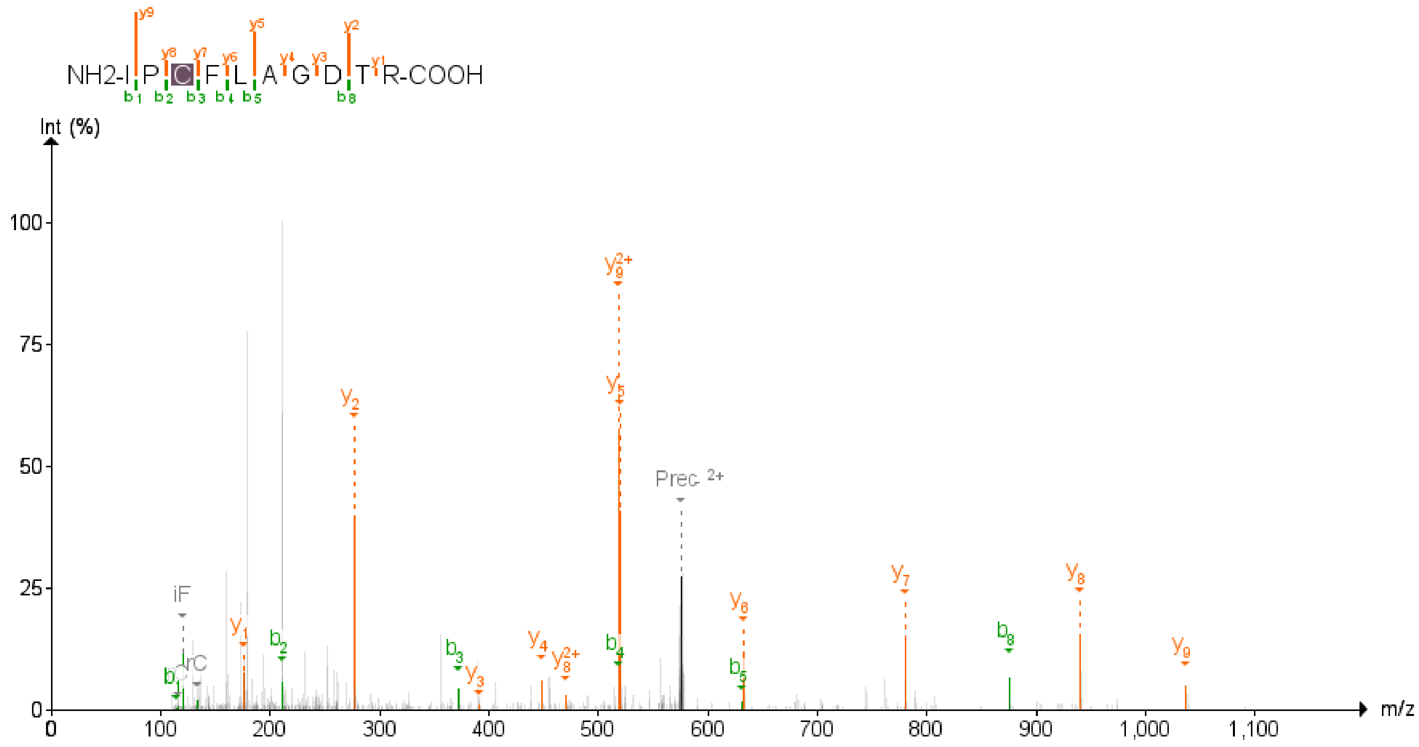

Myeloperoxidase

Sample: T1_2

m/z **575.29**

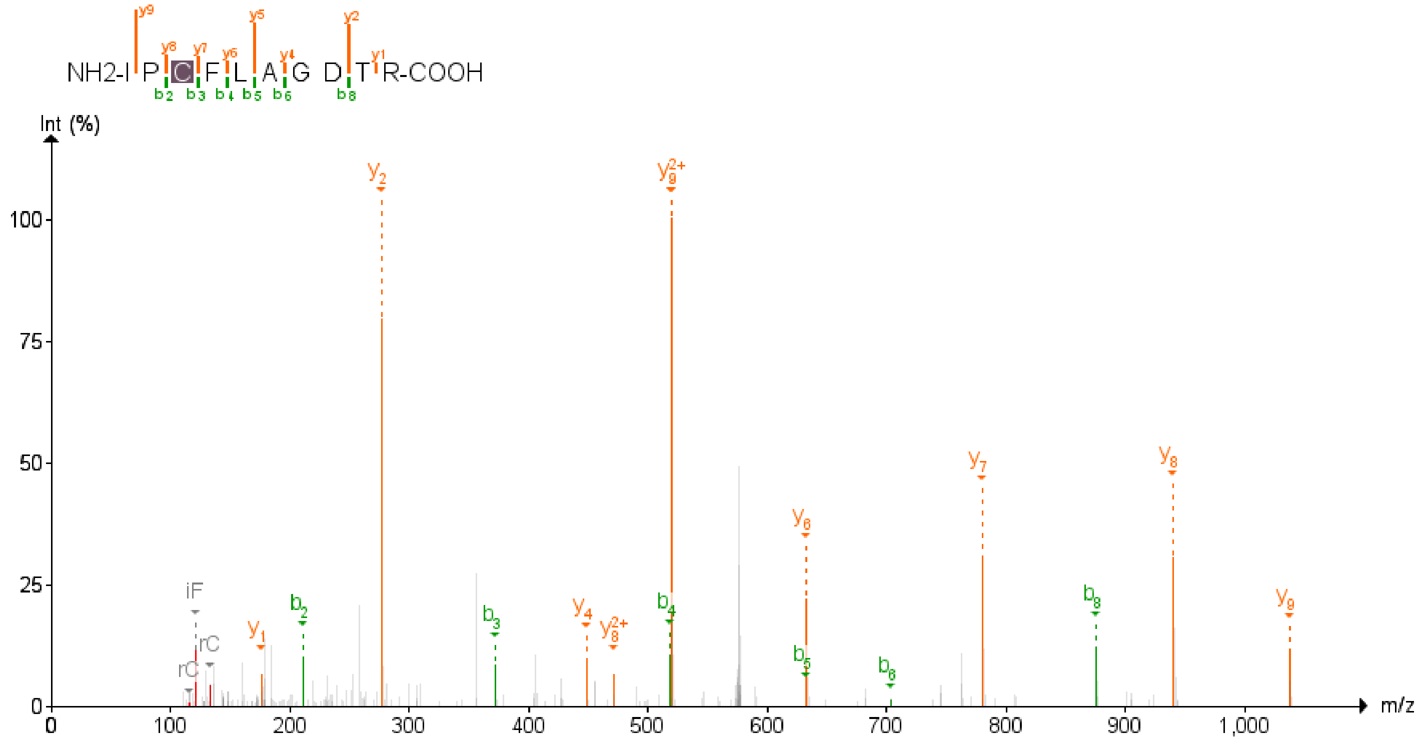

Myeloperoxidase

Sample: MI_1

m/z 723.847

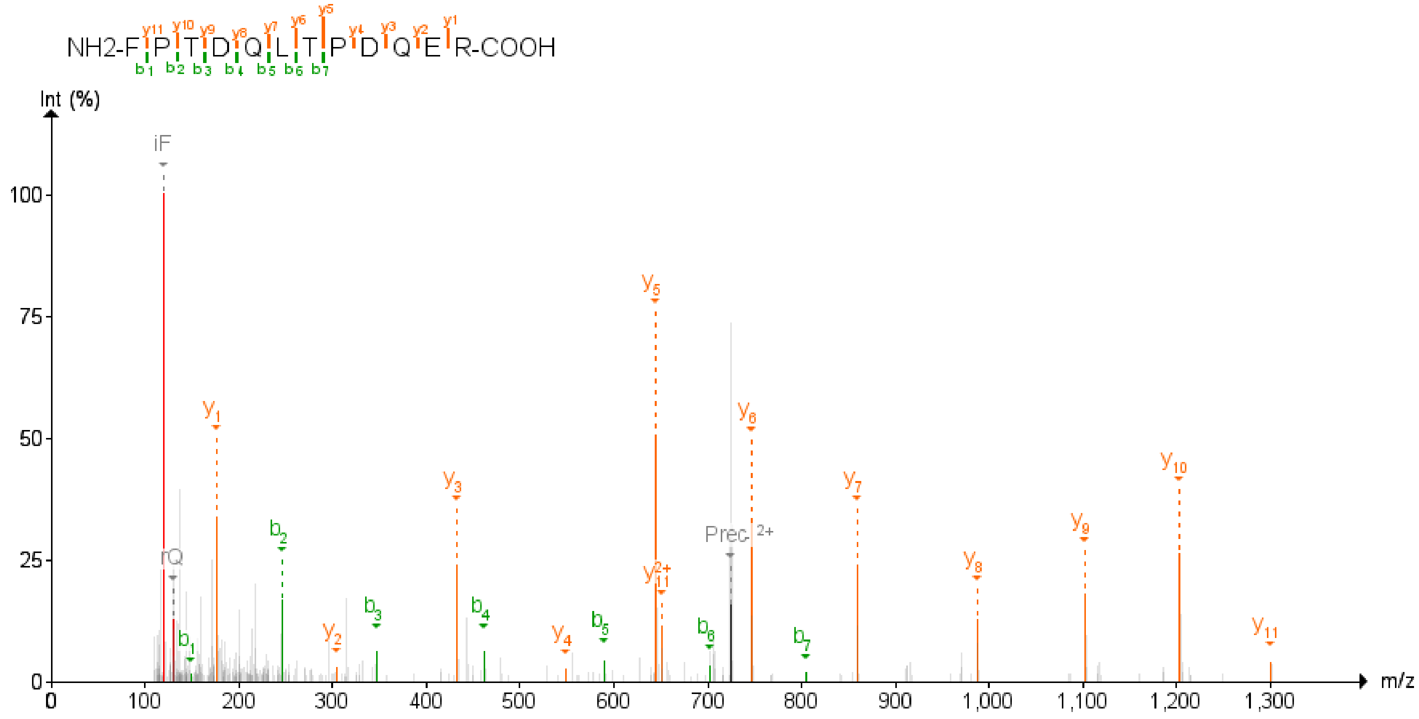

Myeloperoxidase

Sample: MI_2

m/z 723.847

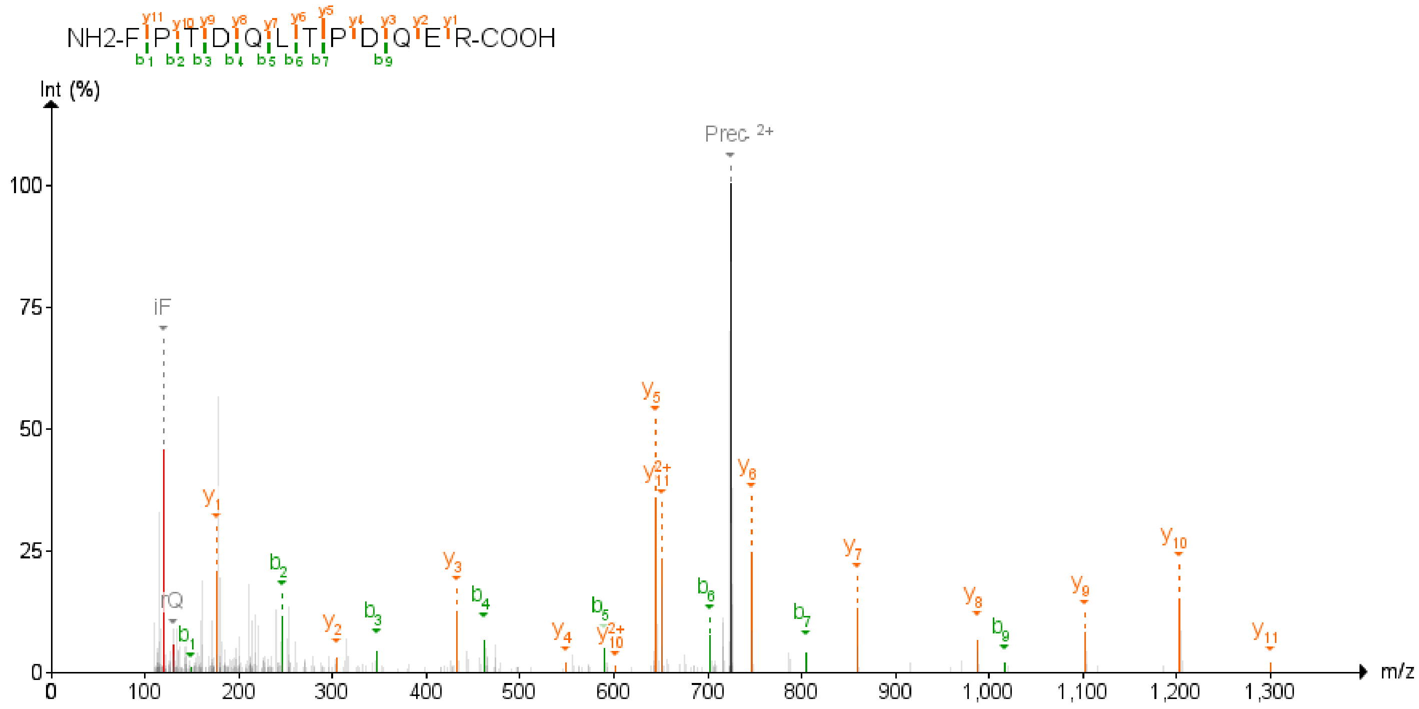

Myeloperoxidase

Sample: MI_4

m/z 451.738

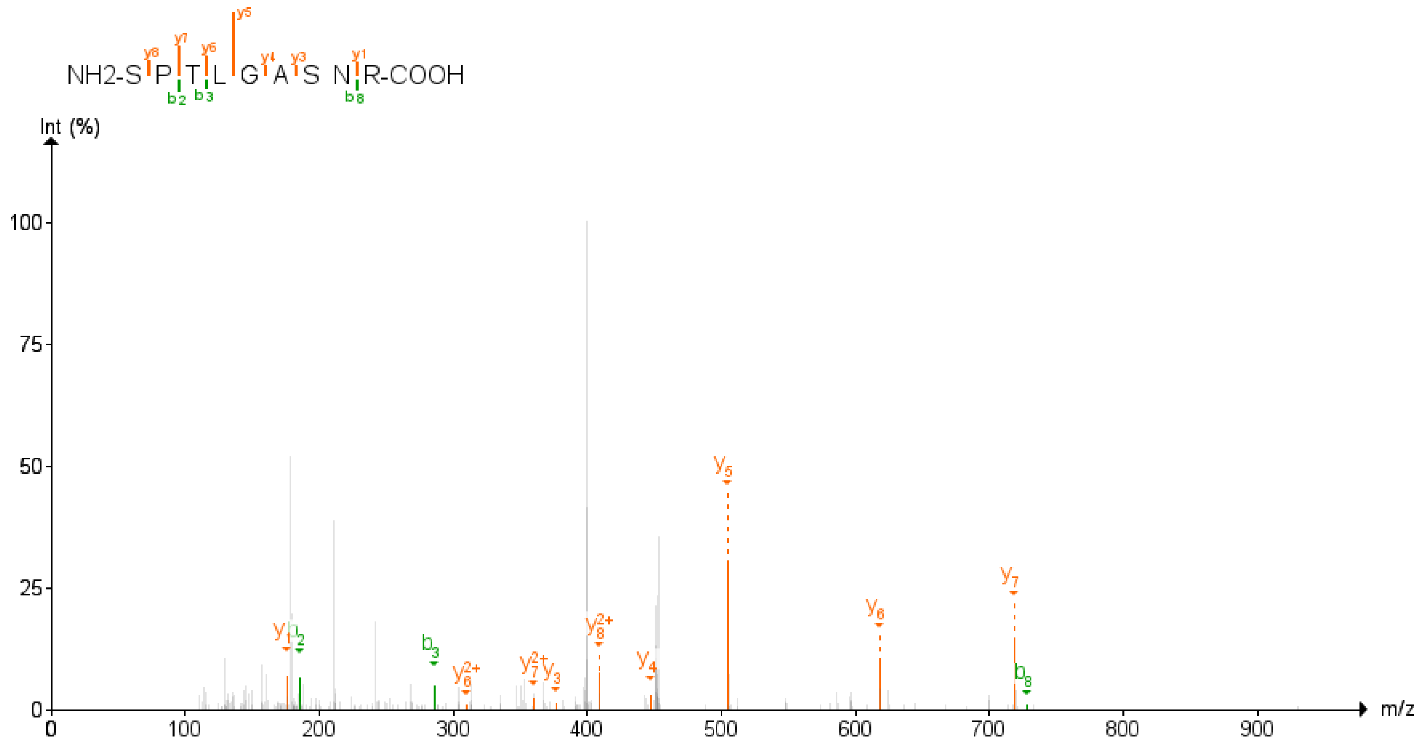

Supplement: Supplementary file 1 [file ijms-26-04891-s001.zip › Relvas-Santos et al. 2025 Sup. data.pdf]
