# Supplementary material for: Glycomics and Glycoproteomics Reveal Distinct Oligomannose Carriers Across Bladder Cancer Stages
Source: Int J Mol Sci. 2025 May 20;26(10):4891. doi: 10.3390/ijms26104891 (PMC12112682; doi:10.3390/ijms26104891)
Supplement: Supplementary file 1 [file ijms-26-04891-s001.zip › Relvas-Santos et al. IJMS_Supplementary Material.pdf]

Glycomics and Glycoproteomics Reveal Distinct Oligomannose Carriers Across Bladder Cancer Stages

Supplementary Materials

Marta Relvas-Santos [1,2,3], Dylan Ferreira [1,2,4], Andreia Brandão [5], Luis Pedro Afonso [1,6], Lúcio Lara Santos [1,4,7,8], André M. N. Silva [2,3,8] and José Alexandre Ferreira [1,2,8,*]

1 Experimental Pathology and Therapeutics Group, Research Center of IPO Porto (CI-IPOP)/CI-IPOP@RISE (Health Research Network), Portuguese Oncology Institute of Porto (IPO Porto)/Porto Comprehensive Cancer Center Raquel Seruca (Porto.CCC Raquel Seruca), 4200-072 Porto, Portugal; marta.relvas.santos@ipoporto.min-saude.pt (M.R.-S.); luis.afonso@ipoporto.min-saude.pt (L.P.A.); lucio.santos@ipoporto.min-saude.pt (L.L.S.)

2 ICBAS-School of Medicine and Biomedical Sciences, University of Porto, 4050-513 Porto, Portugal; andre.silva@fc.up.pt

3 LAQV-REQUIMTE, Department of Chemistry and Biochemistry, Faculty of Sciences, University of Porto, 4169-007 Porto, Portugal

4 School of Medicine and Biomedical Sciences, Fernando Pessoa University, 4420-096 Gondomar, Portugal

5 Cancer Genetics Group, Research Center of IPO Porto (CI-IPOP)/CI-IPOP@RISE (Health Research Network), Portuguese Oncology Institute of Porto (IPO Porto)/Porto Comprehensive Cancer Center Raquel Seruca (Porto.CCC Raquel Seruca), 4200-072 Porto, Portugal; andreia.aguiar.brandao@ipoporto.min-saude.pt

6 Department of Pathology, Portuguese Oncology Institute of Porto (IPO Porto)/Porto Comprehensive Cancer Center Raquel Seruca (Porto.CCC Raquel Seruca), 4200-072 Porto, Portugal

7 Department of Surgical Oncology, Portuguese Oncology Institute of Porto (IPO-Porto), 4200-072 Porto, Portugal

8 GlycoMatters Biotech, 4500-162 Espinho, Portugal

* Correspondence: jose.a.ferreira@ipoporto.min-saude.pt; Tel.: +351-225084000 (ext. 5111)

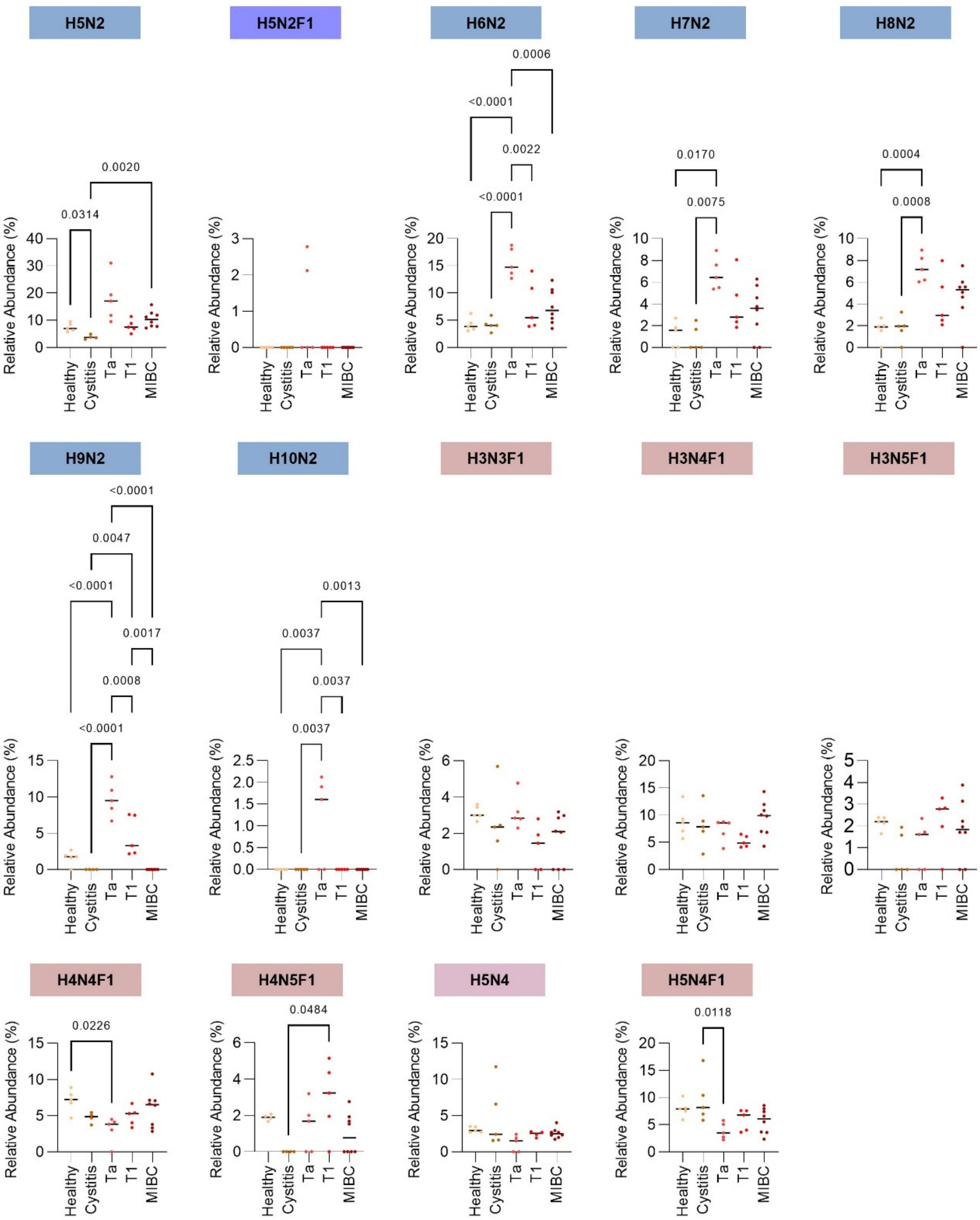
H5N2
0.0020
0.0314
Relative Abundance (%)
Healthy
Cystitis
Ta
T1
MIBC
H5N2F1
H6N2
0.0006
<0.0001
0.0022
<0.0001
H7N2
0.0170
0.0075
H8N2
0.0004
0.0008
H9N2
<0.0001
0.0047
<0.0001
0.0017
0.0008
<0.0001
H10N2
0.0013
0.0037
0.0037
0.0037
H3N3F1
H3N4F1
H3N5F1
H4N4F1
0.0226
H4N5F1
0.0484
H5N4
H5N4F1
0.0118

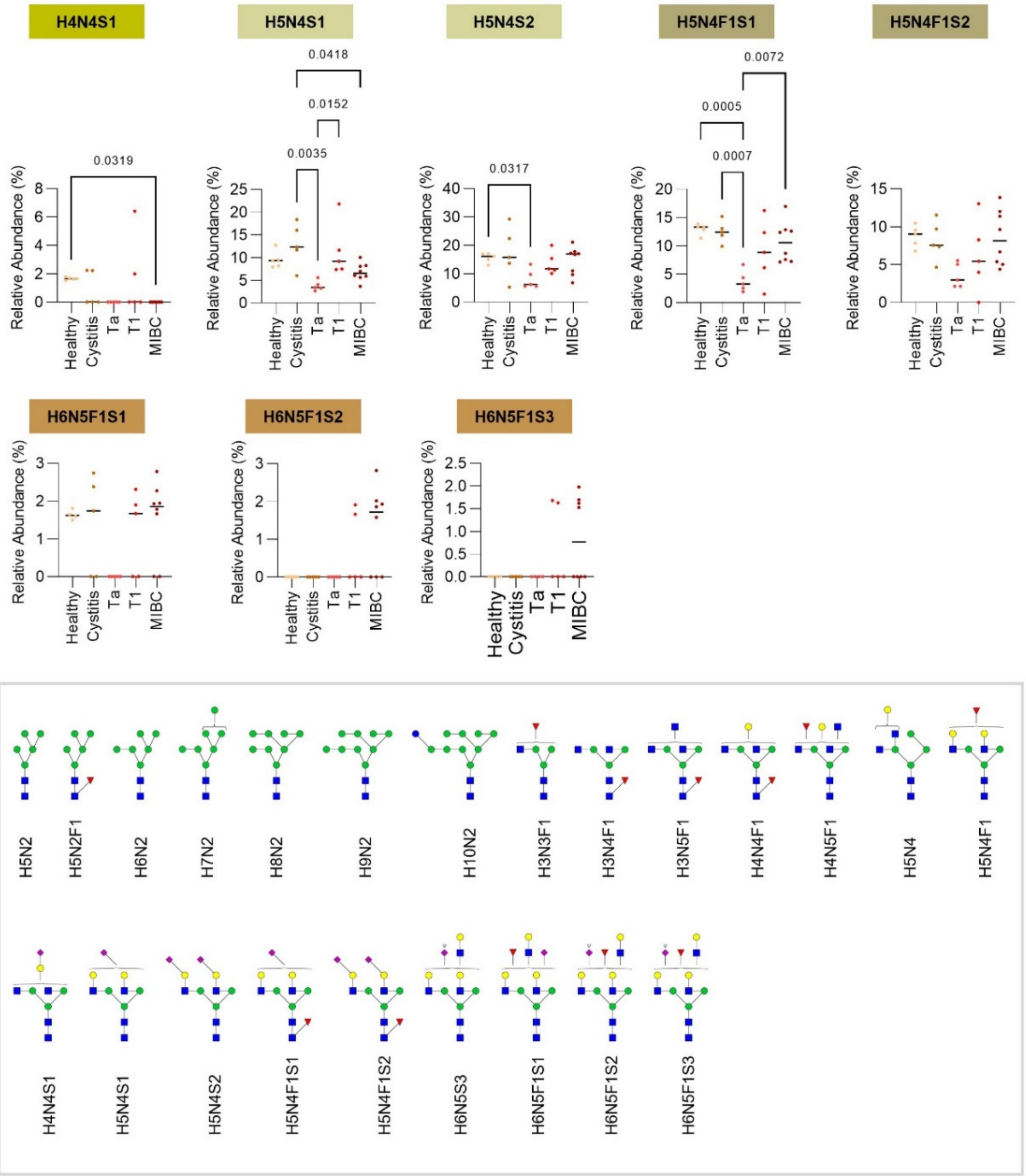

Figure S1. Relative abundance of individual *N*-glycan structures across the five study groups (healthy, cystitis, NMIBC tumors – Ta and T1, MIBC tumors – T2-T4 stages). Several oligomannosidic structures (highlighted in blue) showed statistically significant higher levels in Ta tumors when compared to healthy/inflammatory bladder and other tumor stages. The expression of neutral complex *N*-glycans (highlighted in pink) remained consistent through all bladder conditions. The relative abundance of sialylated complex *N*-glycans (highlighted in green-brown), specifically H5N4S1, H5N4S2, and H5N4F1S1, was significantly lower in Ta tumors when compared to the other bladder groups. H – Hex, N – HexNAc, F – Fuc, S – Neu5Ac. Tests used include One-way ANOVA with or without Welch correction and the Kruskal-Wallis' test. For all analyses, statistical significance was set at $p<0.05$.

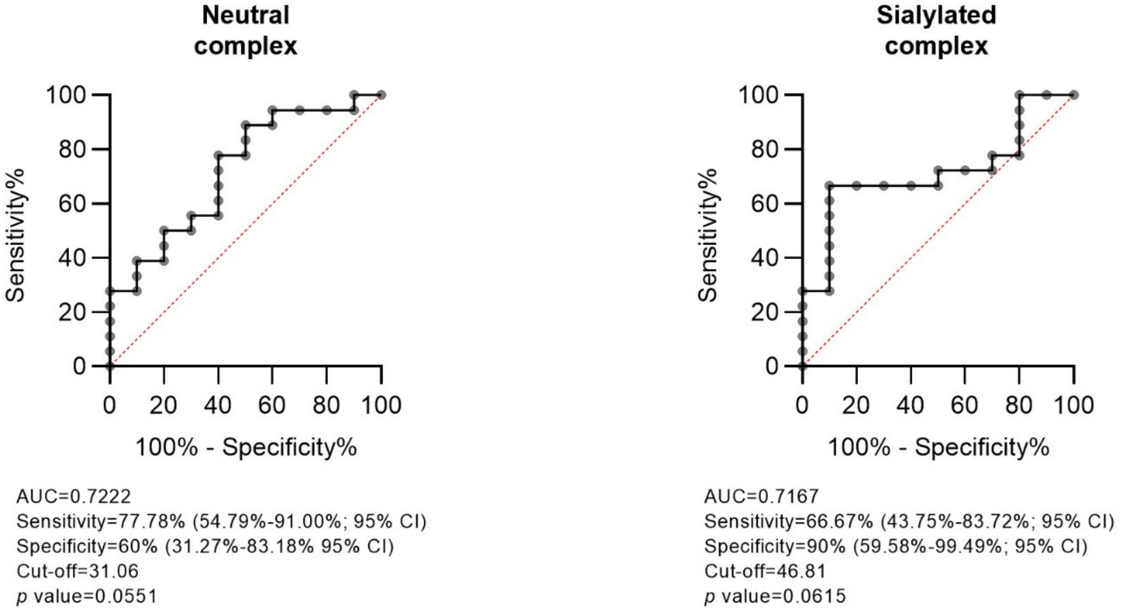

Figure S2. ROC curves evaluating the diagnostic potential of neutral complex and sialylated complex *N*-glycans in distinguishing cancerous (Ta, T1, and MIBC tumors) from healthy and inflammatory bladder. Statistical analysis showed that complex *N*-glycans (relative abundance) are not suitable as diagnostic biomarkers of bladder cancer. Youden Index was used to define the optimal threshold cutoff value and statistical significance was set at $p<0.05$.

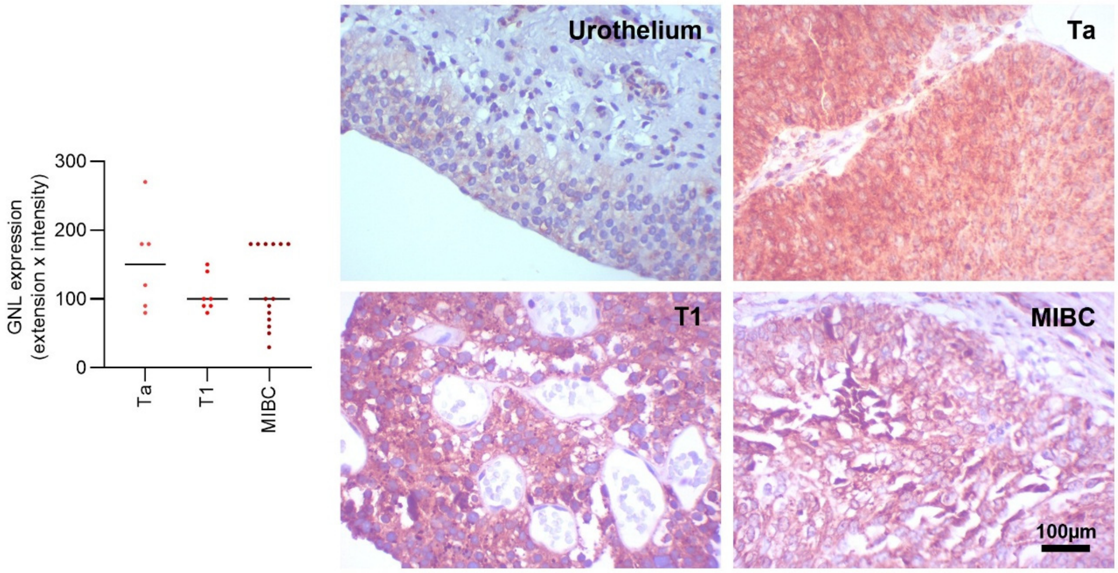

Figure S3. Immunohistochemistry analysis showing that *N*-glycans with affinity for GNL are highly expressed in Ta tumors. Moreover, more aggressive tumors (MIBC) seem to present two distinct groups regarding the expression of GNL ligands. Expression of GNL-binding glycans was observed in both cancer and immune cells, mainly in cytoplasm. One-way ANOVA with Kruskal-Wallis' test was used to evaluate the statistical relationships between GNL expression and disease stage.

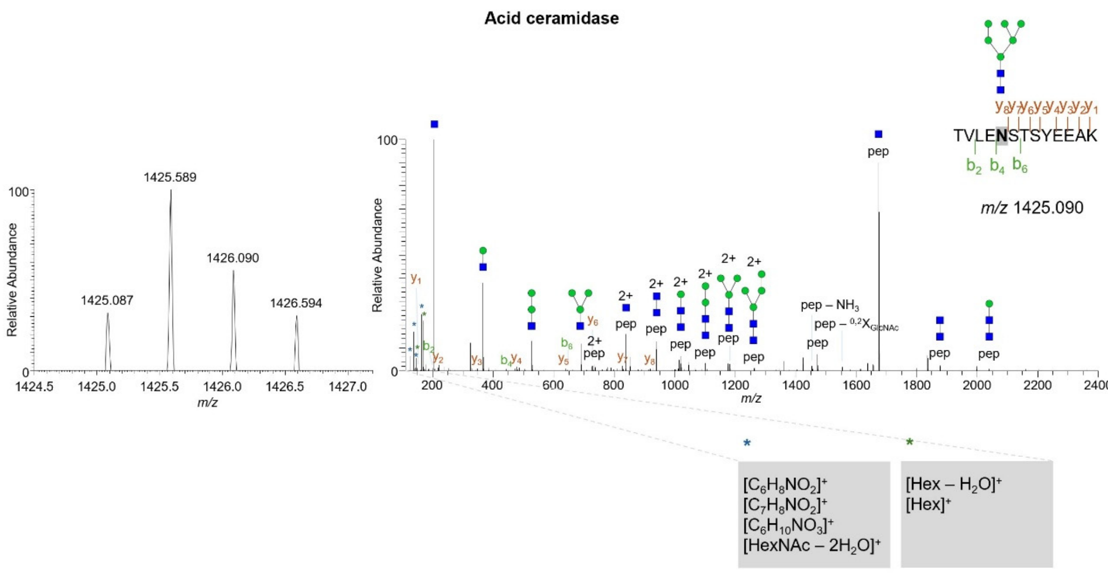

Figure S4. MS and MS/MS spectra for an acid ceramidase glycopeptide with an oligomannose *N*-glycan. MS/MS spectrum shows b/y peptide fragmentation, Y-type ions constituted by the peptide and a portion of glycan structure, and glycan oxonium ions (HexNAc; $HexNAc_1Hex_1$; $HexNAc_1Hex_2$; $HexNAc_1Hex_3$). Moreover, product ions from HexNAc and Hex fragmentation identified in the MS/MS spectrum appear with an asterisk and are highlighted in grey boxes.

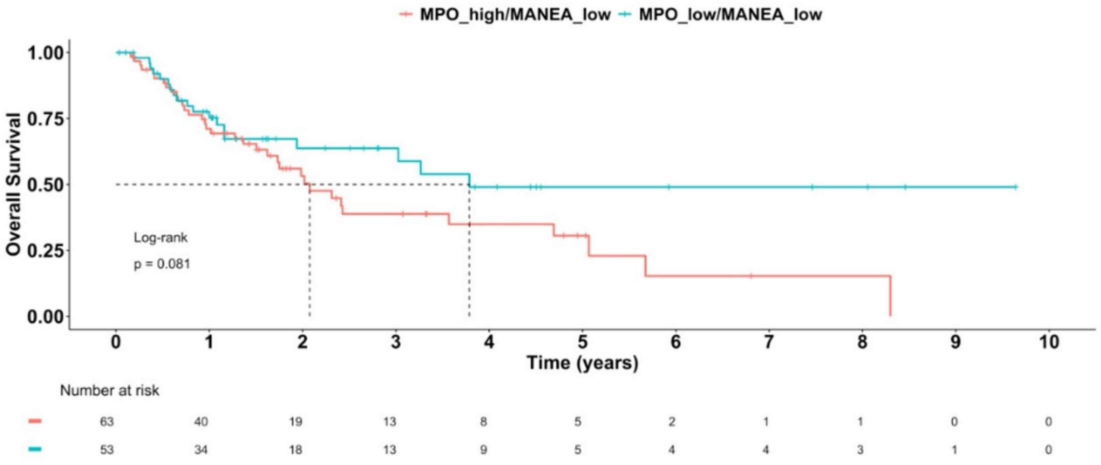

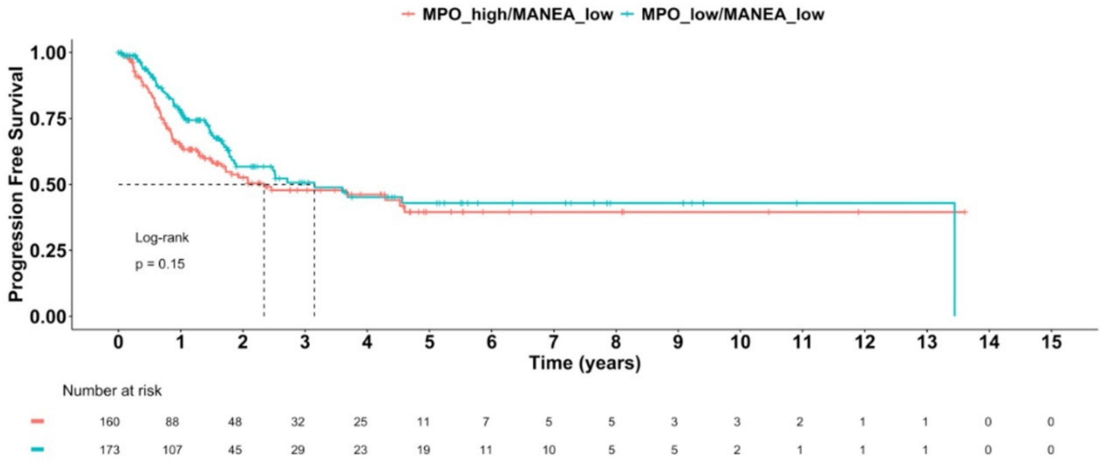

Figure S5. Overall survival and progression-free survival curves for TCGA dataset patients, showing that high expression of *MPO* and low expression of endo-α-mannosidase (*MANEA*) correlates with a worse prognosis. Tumors exhibiting high *MPO* transcript levels and low *MANEA* transcript levels presented a tendency for lower overall survival and progression-free survival compared with tumors presenting low expression of *MPO* and *MAN1A2* transcripts. The log-rank test was employed to compare survival curves, and univariate analysis was employed to evaluate individual prognostic factors.
